# *In vitro* reconstitution of α-pyrone ring formation in myxopyronin biosynthesis†
†Electronic supplementary information (ESI) available. See DOI: 10.1039/c5sc01013f


**DOI:** 10.1039/c5sc01013f

**Published:** 2015-05-18

**Authors:** H. Sucipto, J. H. Sahner, E. Prusov, S. C. Wenzel, R. W. Hartmann, J. Koehnke, R. Müller

**Affiliations:** a Department of Microbial Natural Products , Helmholtz Institute for Pharmaceutical Research Saarland , Building C2 3 , 66123 Saarbrücken , Germany . Email: rolf.mueller@helmholtz-hzi.de; b Department of Drug Design and Optimization , Helmholtz Institute for Pharmaceutical Research Saarland , Pharmaceutical and Medicinal Chemistry , Saarland University , Building C2 3 , 66123 Saarbrücken , Germany; c Helmholtz Centre for Infection Research , Inhoffenstrasse 7 , 38124 Braunschweig , Germany; d Workgroup Structural Biology of Biosynthetic Enzymes , Helmholtz Institute for Pharmaceutical Research Saarland , Building C2 2 , 66123 Saarbrücken , Germany . Email: jesko.koehnke@helmholtz-hzi.de

## Abstract

Myxopyronins are α-pyrone antibiotics produced by the terrestrial bacterium *Myxococcus fulvus* Mx f50 and possess antibacterial activity against Gram-positive and Gram-negative pathogens. They target the bacterial RNA polymerase (RNAP) “switch region” as non-competitive inhibitors and display no cross-resistance to the established RNAP inhibitor rifampicin. Recent analysis of the myxopyronin biosynthetic pathway led to the hypothesis that this secondary metabolite is produced from two separate polyketide parts, which are condensed by the stand-alone ketosynthase MxnB. Using *in vitro* assays we show that MxnB catalyzes a unique condensation reaction forming the α-pyrone ring of myxopyronins from two activated acyl chains in form of their β-keto intermediates. MxnB is able to accept thioester substrates coupled to either *N*-acetylcysteamine (NAC) or a specific carrier protein (CP). The turnover rate of MxnB for substrates bound to CP was 12-fold higher than for NAC substrates, demonstrating the importance of protein–protein interactions in polyketide synthase (PKS) systems. The crystal structure of MxnB reveals the enzyme to be an unusual member of the ketosynthase group capable of binding and condensing two long alkyl chains bound to carrier proteins. The geometry of the two binding tunnels supports the biochemical data and allows us to propose an order of reaction, which is supported by the identification of novel myxopyronin congeners in the extract of the producer strain. Insights into the mechanism of this unique condensation reaction do not only expand our knowledge regarding the thiolase enzyme family but also opens up opportunities for PKS bioengineering to achieve directed structural modifications.

## Abstract 2

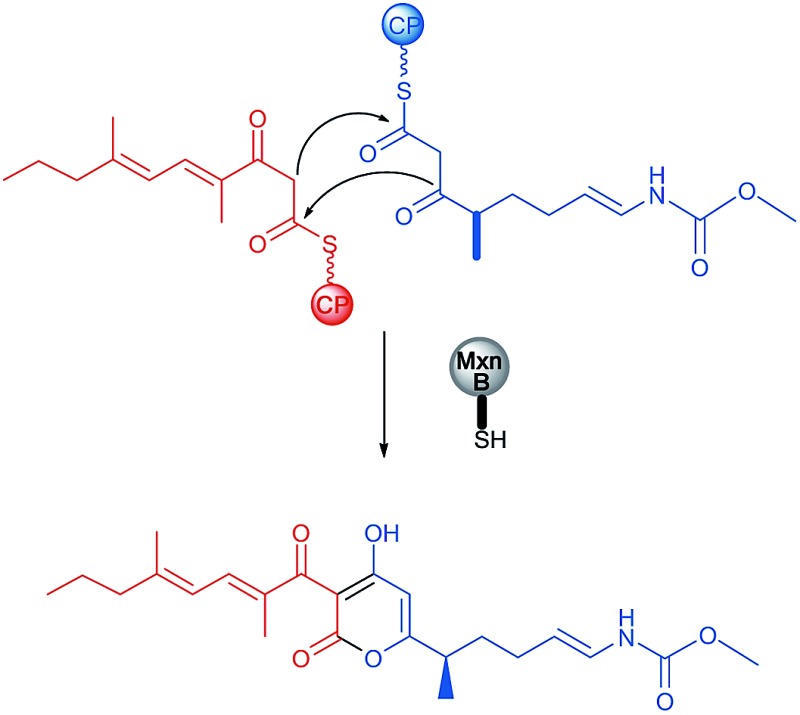
α-Pyrone rings exist in many polyketide synthase (PKS) derived natural products. We report the first *in vitro* reconstitution of α-pyrone ring formation by a type I PKS using chemically synthesized substrates.

## Introduction

Pathogens are becoming resistant to clinical antibiotics at an accelerating rate. Hence there is an urgent need for new antibacterials, which either act upon new antibiotic targets or exploit novel binding sites in established ones.^1^ Myxobacteria are a rich source of bioactive compounds, most of which are derived from polyketide synthase (PKS) and non-ribosomal peptide synthetase (NRPS) pathways.^2^ The underlying biosynthetic principles provide a fascinating modular platform to accomplish difficult chemical reactions and became a major source for current clinical drugs. PKS systems are classified into various subtypes according to their functions and architecture.^3^ To date, three types of bacterial PKSs are known; type I PKSs are composed of multifunctional enzymes organized in modules responsible for non-iterative catalytic steps of one cycle of polyketide chain elongation.^4^ Type II PKSs are multi-enzyme complexes that carry a single set of iteratively acting activities.^5^ Type III PKSs, or chalcone synthase-like PKSs, also act iteratively as condensing enzymes.^6^ One hallmark activity of all PKS pathways are ketosynthases (KSs), usually catalyzing the Claisen condensation of an acyl thioester and a malonyl thioester.

**Scheme 1:**
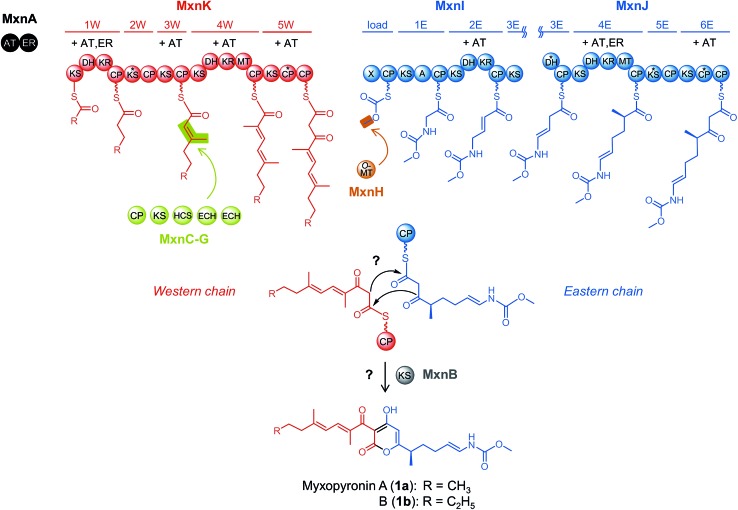
Model for myxopyronin biosynthesis based on analyses of the native producer strain *Myxococcus fulvus* Mx f50.^12^ Western and eastern chain biosynthesis are catalyzed by two separate assembly lines, MxnK shown in red and MxnI/J shown in blue. Abbreviations for assembly line domains: A: adenylation domain, CP: carrier protein domain, DH: dehydratase domain, KR: ketoreductase domain, KS: ketosynthase domain, MT: methyltransferase domain, X: putative inactive KR or truncated phosphoglucomutase/phosphomannomutase domain. Domains marked with an asterisk are assumed to be inactive; assembly line modules 2W, 3E and 5E do presumably not participate in chain elongation. Required acyltransferase (AT) and enoylreductase (ER) activities are indicated for each module and supplied in *trans* by MxnA. The module 3W intermediate is modified by a β-branching cassette shown in yellow-green consisting of a CP (MxnC), a KS (MxnD), a hydroxymethylglutaryl (HMG)-CoA synthase (HCS; MxnE) and two enoyl-CoA hydratases/isomerases (ECH; MxnF and MxnG). As highlighted in orange, the eastern chain starter unit is modified by an *O*-methyltransferase (*O*-MT; MxnH). After chain assembly, the KS MxnB is proposed to catalyze pyrone ring formation, which is investigated in the present study.

Myxopyronins (**1**) belong to the α-pyrone compound class and have been isolated from the Gram-negative soil bacterium *Myxococcus fulvus* Mx f50.^7^ Myxopyronins (together with corallopyronin (**2**) and ripostatin) bind to the “switch region” of bacterial RNA polymerase (RNAP), which represents a novel binding site for RNAP-binding antibiotics. They also display a different mode of inhibition compared to existing RNAP-targeting drugs. Since RNAP is a highly conserved protein, myxopyronins represent a very promising compound class for the development of broad spectrum antibacterial therapeutic agents.^8^,^9^ Feeding studies and the analysis of the biosynthetic gene cluster revealed that myxopyronins as well as corallopyronins are most likely derived from two linear polyketide chains, the eastern and western parts of the molecule. These chains were proposed to be produced by two distinct multimodular PKS/NRPS megasynthetases (Scheme 1).^10^–^12^ It has been postulated that in the last step of myxopyronin and corallopyronin biosynthesis the fully matured β-keto intermediates of the eastern and western chains undergo condensation catalyzed by the stand-alone ketosynthase (KS) enzyme MxnB and CorB, respectively, to form the characteristic α-pyrone ring.^11^,^12^ The α-pyrone ring structure proved to significantly contribute to the bioactivity of myxoypronin since the replacement of the pyrone ring with the more stable *N*-methyl pyridone or phenol led to the loss of its antibacterial activity.^13^ Furthermore, the crystal structure of RNAP with myxopyronin showed interactions of the oxygens of the α-pyrone ring with RNAP residues indicating the importance of this moiety towards its bioactivity.^8^

**Fig. 1:**
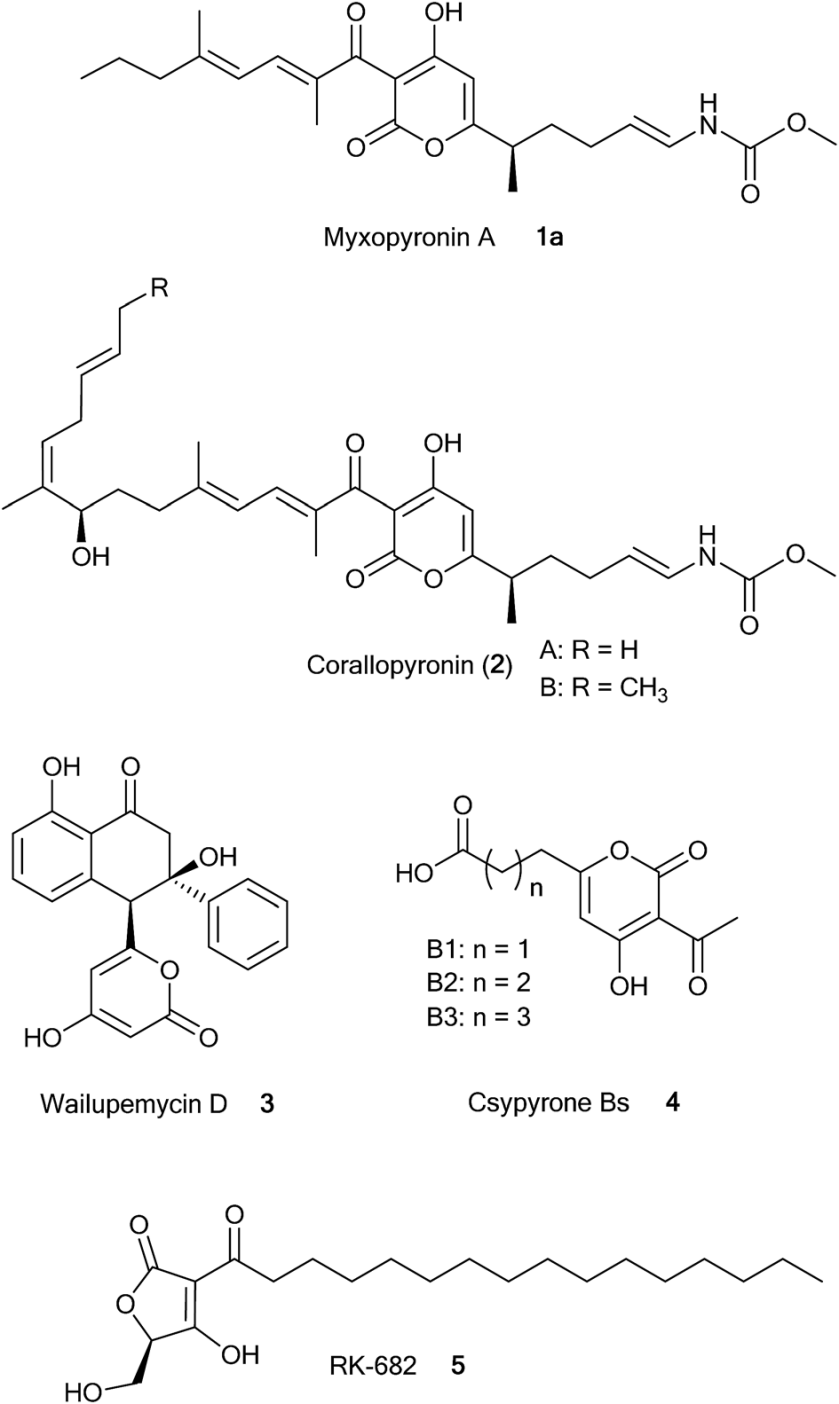
Structures of natural products, which are biosynthesized by type I, II, and III PKSs and contain α-pyrone rings (**1a** and **2**, **3**, and **4** respectively). Compound **5** is generated by a type I PKS and contains a tetronate ring.

In fact, there are a number of natural products containing pyrone rings derived from type II and type III PKS systems such as wailupemycin^14^ and csypyrone,^15^ respectively (Fig. 1). Recently, CsyB has been biochemically characterized as a type III PKS that couples two β-ketoacyl-CoAs, forming the α-pyrone ring in csypyrone.^16^ Only one example of 5-membered ring formation by a type I PKS has been reported so far; a stand-alone KS (RkD, 22% sequence identity to MxnB) condenses the carrier protein (CP)-bound substrate, forming the tetronate ring during the biosynthesis of the phosphatase inhibitor RK-682.^17^ To the best of our knowledge, to date 6-membered pyrone ring formation by a stand-alone KS has not been reported for a type I PKS pathway.

Here we demonstrate that MxnB is responsible for the condensation of the eastern and the western chains giving rise to myxopyronin. We show that MxnB exhibits substrate flexibility for β-ketoacyl intermediates linked through a thioester bond to either NAC or CP. In addition, the crystal structure of MxnB reveals an overall typical thiolase fold, which is altered to accommodate the binding and condensation of two long alkyl chains. Structural analysis supports the biochemical findings and allows us to propose an order of reaction for α-pyrone ring formation in myxopyronin biosynthesis.

## Results and discussion

### MxnB is responsible for α-pyrone ring formation in myxopyronins

MxnB belongs to the thiolase superfamily of proteins that catalyzes the formation of carbon–carbon bonds *via* Claisen condensation. BLAST search results identified MxnB as a homolog of FabH, a β-ketoacyl-[acyl carrier protein (ACP)] synthase III (KAS III), highlighted by the presence of the archetypical cysteine–histidine–asparagine (CHN) catalytic triad.^18^,^19^ The catalytic cysteine (C112 in FabH from *E. coli*) is responsible for the transacylation process, while the histidine and asparagine residues are required for the Claisen-like condensation reaction, interacting with the incoming malonylated CP to enable enolization of the substrate.^19^

We could not identify any protein exhibiting more than 40% sequence identity to MxnB which has been previously biochemically characterized (Table S2†). Based on the CHN catalytic triad, KAS III belongs to the same subgroup as chalcone synthase (CHS) enzymes in the thiolase superfamily.^4^ CHSs act as homodimeric iterative PKSs catalyzing a series of decarboxylation, condensation, and cyclization reactions.^14^,^15^ Phylogenetic analysis places MxnB between FabH-like and CHS-type enzymes (Fig. S1†). Therefore, characterization of MxnB can be seen as a valuable addition to widen the scope of biochemical mechanisms catalyzed within the KAS III family.

**Fig. 2:**
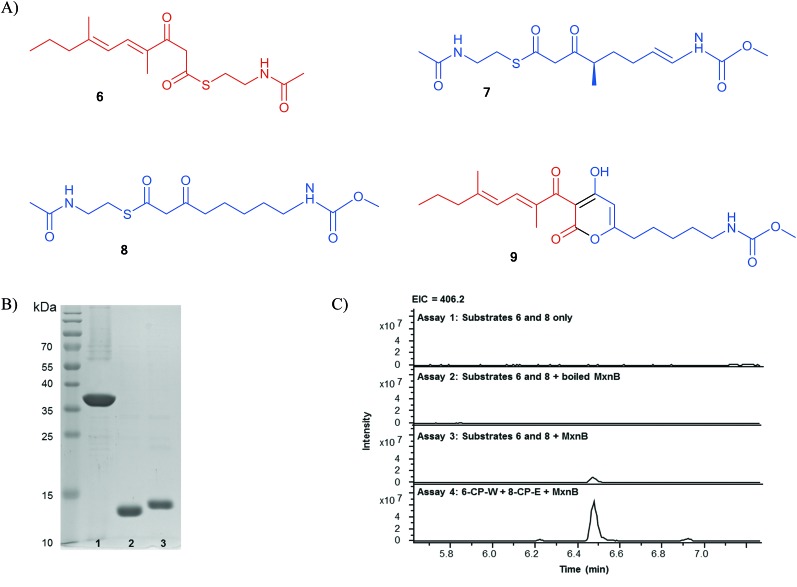
Substrates and myxopyronin analogue, SDS page and LC-MS analysis of *in vitro* assays. (A) NAC substrates as native western mimic (**6**), native eastern mimic (**7**), analogue mimic of native eastern (**8**), and myxopyronin derivative (**9**). (B) Lane 1: His-MxnB (38.7 kDa), lane 2: His-CP-E co-expressed with MtaA (14.7 kDa), and lane 3: His-CP-W co-expressed with MtaA (14.6 kDa) on a 15% SDS gel; (C) LC-MS analysis of *in vitro* assays showing extracted ion chromatograms (EIC) [M + H]^+^ = 406.2: assay 1 (**6** and **8** only); assay 2 (**6**, **8**, and boiled MxnB); assay 3 (**6**, **8**, and MxnB); assay 4 (**6**-CP-W, **8**-CP-E, and MxnB).

In order to provide first experimental evidence that MxnB is responsible for α-pyrone ring formation we aimed to establish the cyclization reaction *in vitro*. Soluble recombinant MxnB protein with an N-terminal His_6_-tag was overproduced in *E. coli* BL21 (DE3) and purified using one-step Ni-NTA affinity chromatography, to yield 0.3 mg of MxnB from 100 mL of liquid culture. The purified His-tagged MxnB protein had the expected molecular weight of approximately 38.7 kDa (Fig. 2B). The enzyme was incubated with NAC thioesters of the western (**6**) and eastern chains (**7**), serving as mimics of the CP's phosphopantetheine arm bound substrates (Fig. 2A). During biosynthesis the western and eastern chains are assumed to be bound to CP-W5 and CP-E6, respectively (Scheme 1). Analysis of the reaction products using LC-MS showed the formation of **1a** exhibiting an MS^2^ fragmentation pattern that was identical to authentic myxopyronin A (Fig. S2†).

The limited quantity of available native eastern NAC thioester forced us to use an analogous eastern chain lacking a methyl group and a double bond (**8**) for detailed *in vitro* studies (Fig. 2A). This eastern chain mimic (**8**) was found to be loaded onto MxnB equally well as the native eastern chain (**7**) (Fig. S2†). When the experiment was repeated using this modified eastern chain a new product corresponding to the expected myxopyronin derivative **9** (Fig. 2C, assay 3) was detected in the LC-MS analysis. High-resolution mass spectrometry (HRMS) of the compound displayed an ion with *m*/*z* 406.2221 (calc. for C_22_H_32_NO_6_, 406.2224), which was assigned as [M + H]^+^ of **9**. Its fragmentation was slightly different to standard myxopyronin A, which is due to the variance in the eastern part of the molecule (Fig. S3†). In order to confirm the structure of this novel derivative, it was isolated from an *in vitro* assay and subjected to NMR analysis (Table S3, Fig. S4†). To rule out a non-enzymatic condensation, the assay was repeated in the absence of MxnB (assay 1) and in the presence of heat-inactivated MxnB (assay 2). In both cases the reaction did not occur (Fig. 2C). These data provided clear evidence that MxnB is the enzyme responsible for pyrone ring formation.

The small amount of product formed when using NAC substrates suggested that the interaction between cognate CPs and MxnB might be an important factor for efficient condensation. It has been shown that CP-bound substrates were processed much faster than the corresponding NAC thioesters in a study of 6-deoxyerythronolide B synthase.^20^ To optimize the *in vitro* assay we attempted to mimic the *in vivo* conditions by overexpressing CPs from the last module of the western (CP-W5) and eastern (CP-E6) chain assembly lines (hereafter referred to as CP-W and CP-E, respectively). Soluble recombinant CP-W and CP-E proteins with N-terminal His_6_-tags were overproduced in *E. coli* BL21 (DE3) cells and purified by one-step Ni-NTA affinity chromatography, to yield 2.5 mg of each, CP-W or CP-E, from 100 mL of liquid culture. The purified His-tagged CP-W and CP-E proteins had molecular weights of 14.3 kDa and 14.4 kDa, respectively. To simplify the phosphopantetheinylation of the CPs, CP-W and CP-E were phosphopantetheinylated *in vivo* by co-expressing *mtaA*, a gene encoding a broad spectrum 4′-phospantetheinyl transferase from *Stigmatella aurantiaca*.^21^ We thereby obtained *holo*-CP-W and *holo*-CP-E exhibiting masses of 14.6 kDa and 14.7 kDa, respectively (Fig. 2B and S5 and S6†).

It is known that CPs are able to perform self-acylation using CoA or NAC esters.^22^ Thus, we analyzed the ability of *holo*-CP-W and *holo*-CP-E to conduct self-acylation with substrates **6** and **8**, respectively. LC-MS analysis of the product showed a mass shift of 192 Da for *holo*-CP-W indicating the formation of **6**-*S*-CP-W and a mass shift of 213 Da for *holo*-CP-E in agreement with the generation of **8**-*S*-CP-E (hereafter referred to as **6**-CP-W and **8**-CP-E, respectively) (Fig. S5 and S6†).

Incubation of MxnB, **6**-CP-W, **8**-CP-E resulted in a 12-fold increase of product formation compared to the reactions with NAC substrates (Fig. 2C, assay 4). This revealed the importance of CP-MxnB interactions for pyrone ring formation. It also highlights the effect of protein–protein interactions compared to substrate–protein interactions in PKS and especially carrier protein dependent biosynthetic systems.^23^

### Overall structure of MxnB

**Fig. 3:**
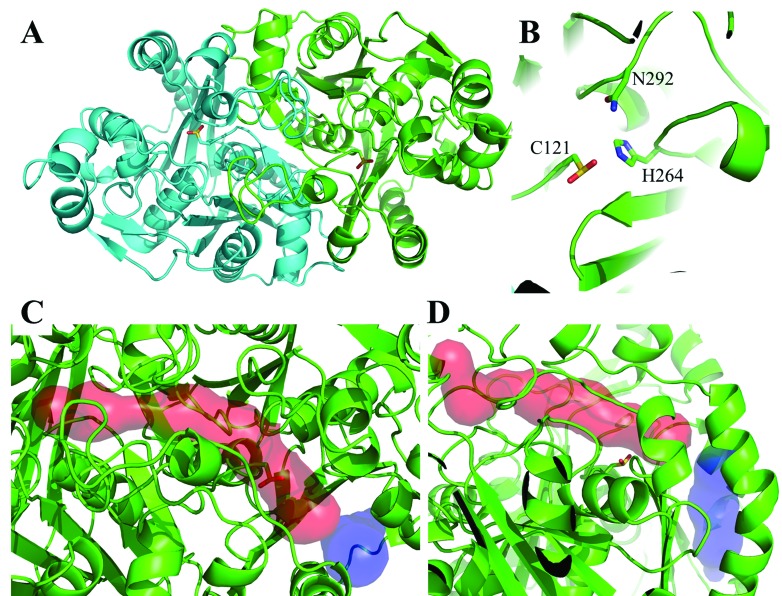
(A) Cartoon representation of the MxnB dimer structure, with monomers colored in cyan and green. The active-site cysteines (C121) are shown as sticks. (B) Cartoon representation of the MxnB active site. The residues comprising the catalytic triad are shown as sticks. (C) Top view and (D) side view of the two proposed substrate-binding tunnels in MxnB. Canonical tunnel (red) and additional tunnel (blue). Tunnels were predicted using Mole 2.0.^27^

Since the condensation of western and eastern chain requires MxnB to bind two long acyl chain substrates we intended to investigate if there is a structural rationale for this ability. Full-length MxnB (335 residues) with an N-terminal His_6_-tag was expressed and purified as described in the materials and methods section. MxnB crystals belonged to space group *P*1 and diffracted to 1.67 Å. The structure of MxnB (PDB ID ; 4V2P) was determined by molecular replacement using the *E. coli* FabH structure (PDB ID ; 1EBL) as a search model. The crystals contained one biological dimer in the asymmetric unit and the refined model includes residues 8–335 in chain A and 7–335 in chain B (Fig. 3A). The missing residues are presumed to be disordered. The MxnB monomer displays the overall typical ketosynthase fold consisting of a five-layered core structure, α-β-α-β-α, similar to that of thiolase I.^24^ Although the sequence identity of MxnB to *E. coli* FabH is low (29%), pairwise alignment of the structures gives a C_α_ rmsd of just 1.5 Å over 254 (monomer) and 2.1 Å over 508 (dimer) residues, respectively. The active site of MxnB contains the catalytic CHN triad (C121, H264 and N292, Fig. 3B). The active site C121 is oxidized to cysteine sulfinic acid, but this modification appears to be either transient or a result of crystallization/data collection, since LC-MS analysis of the intact protein showed the expected mass for MxnB with unmodified cysteines.

Unlike other ketosynthases such as FabH, MxnB is required to bind and condense two long alkyl chains bound to carrier proteins. The only ketosynthase catalyzing a similar reaction characterized structurally to date (although from a completely unrelated pathway) is OleA.^25^ This enzyme aids in synthesizing long chain olefins in *Xanthomonas campestris* and was proposed to possess a second substrate-binding tunnel. Although sequence conservation is low (25% over 301 residues) the structures are reasonably well conserved (C_α_ rmsd of 2.5 Å over 231 residues, PDB ID ; 3S23). It was argued that the two helices forming the new hydrophobic binding tunnel were spread apart further in OleA than in other FabH-like structures, where binding of a second long alkyl chain is not required (Fig. S7†).^25^

The enzyme CsyB was very recently reported to carry out a similar α-pyrone ring formation reaction.^26^ The sequence identity between MxnB and CsyB (PDB ID ; 3WXY) is 17% and pairwise alignment of the structures gives a C_α_ rmsd of 3.7 Å over 262 residues (monomer). The core of both proteins is quite similar on the level of secondary structure and the main structural differences can be found in the periphery, removed from the active site. The relative position of the catalytic triad forming residues (CHN) is conserved in both proteins. CsyB has an additional histidine (H377), which was described as essential for enzymatic activity. In MxnB, this position (CsyB H377) is occupied by a serine (S324). Compared to the structures of MxnB and OleA, the proposed second substrate-binding tunnel is blocked in CsyB.

In MxnB the catalytic C121 is located at a crossroads between two substrate-binding tunnels (Fig. 3C and D). The first, canonical, tunnel (red) and the second (blue) tunnel, positioned as proposed for OleA, have amphipathic character (based on electrostatic surface potential maps generated in ccp4mg^28^). The position of the catalytic C121 in relation to both tunnel entrances strongly suggests that binding of substrate in the blue tunnel and its subsequent transacylation from the CP to MxnB C121 results in the obstruction of the red tunnel entrance. Therefore, we propose that binding of substrate in the red tunnel would have to precede binding in the blue tunnel. Since only one major product is formed by MxnB, it is likely that each tunnel has a chain preference (“eastern chain tunnel” and “western chain tunnel”). Based on the above-mentioned geometry considerations, the substrate accepted by the tunnel marked in red is transferred first during the transacylation process.

### Initial biochemical characterization of MxnB

#### Catalytic site of MxnB

In general, condensation reactions catalyzed by KSs start by loading of the enzyme with the acyl chain. The chain is transferred from the CP and covalently attached to the active site cysteine residue *via* a thioester bond prior to condensation. We investigated the covalent attachment of **6** or **8** to MxnB and LC-MS analyses confirmed transfer of both substrates to the enzyme (Fig. S8†). We then expressed and purified the putative active site mutant C121A (MxnB^#^) to investigate if substrates could still be transferred. We did not observe any mass shift after incubation of MxnB^#^ with **6** or **8** and no myxopyronin derivative was formed employing this mutant (loss of catalytic function). These results confirmed that C121 is the catalytic cysteine playing a major role in the transacylation process.

#### Substrate preference for MxnB priming

**Fig. 4:**
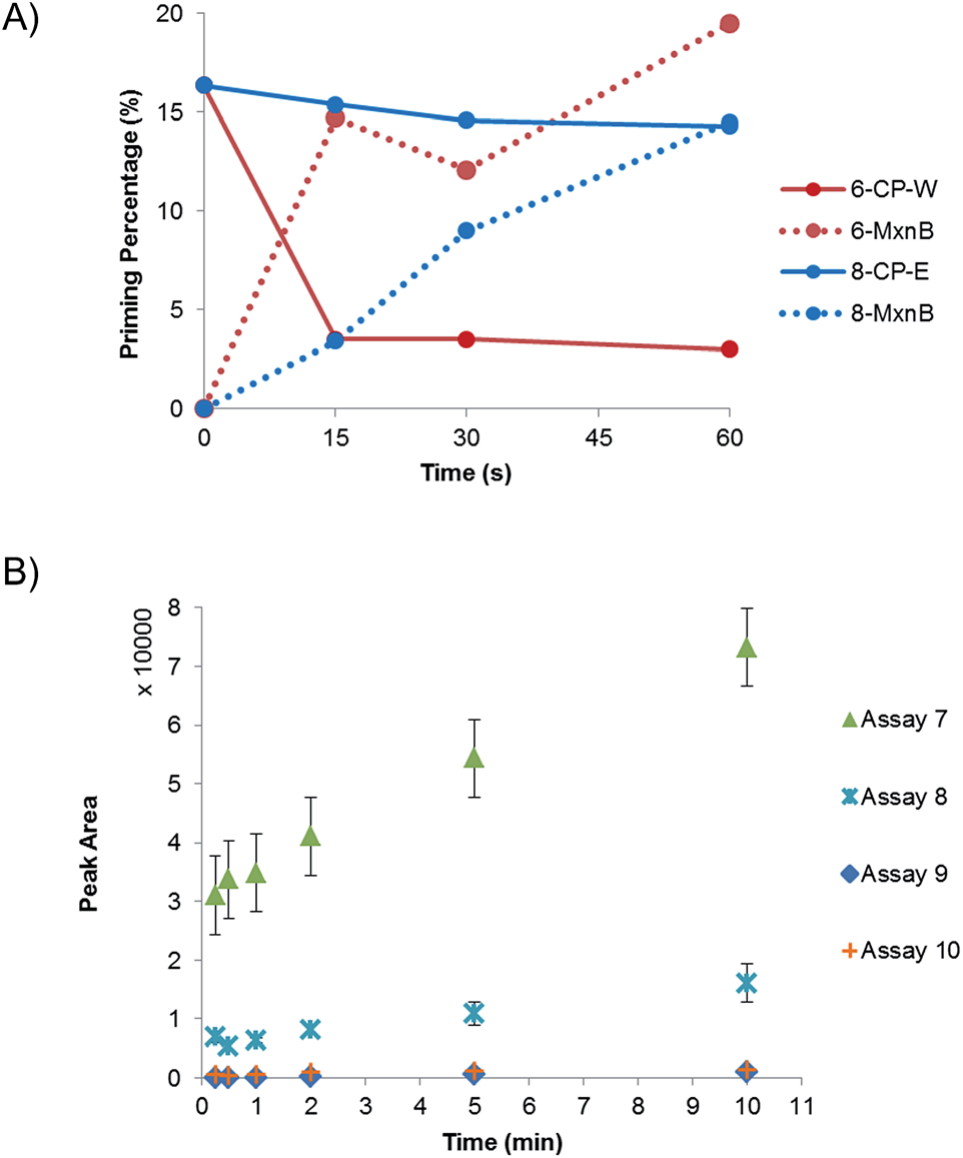
LC-MS analysis of substrate transfer and competition assays. (A) Percentage of primed MxnB and the remaining loaded-CP. (B) Time-course for the production of **9** from 15 s to 10 min. Peak area of extracted ion chromatograms for **8** were used for quantification. Assay 7–10: assay 7 (**6**-MxnB and **8**-CP-E), assay 8 (MxnB, **6**-CP-W and **8**-CP-E), assay 9 (**8**-MxnB and **6**-CP-W), and assay 10 (MxnB, **6**, and **8**). Values for assay 9 and 10 data points overlay. Error bars represent the standard error of the mean from three repeats.

To obtain deeper insights into the reaction mechanism we first addressed the question whether the eastern or western chain substrate is preferentially loaded onto MxnB prior to condensation with the second chain. We performed transacylation experiments with MxnB and primed CPs (**6**-CP-W and **8**-CP-E). Free substrate was completely removed before incubation with MxnB to avoid any interference in the assays. As complete CP priming could not be achieved *via* self-acylation, the percentages of substrate-loaded CPs were normalized using the deconvoluted mass peak heights in order to establish comparable assay conditions.^29^ After incubation of MxnB with a slight excess of either **6**-CP-W (assay 5) or **8**-CP-E (assay 6) the substrate transfer was analyzed at specific time-points using LC-MS. As shown in Fig. 4A, the percentage of **6**-CP-W (in relation to unprimed CP-W) decreased much faster than the amount of **8**-CP-E (in relation to CP-E). At the same time, the amount of primed MxnB increased faster in the western chain assay than in the eastern chain assay (**6**-MxnB *vs.***8**-MxnB). These results suggest that during myxopyronin biosynthesis MxnB is first loaded with the western chain and then condensed with the eastern chain to form the α-pyrone ring structure.

#### Substrate preference for the condensation reaction

To further investigate this finding we set up a series of *in vitro* condensation assays including experiments where MxnB was already preloaded with either **6** or **8** to give **6**-MxnB and **8**-MxnB, respectively. The percentages of substrate-loaded MxnB were calculated using the same method as applied to the CPs. The experiments were set up as follows: assay 7 (**6**-MxnB and **8**-CP-E), assay 8 (MxnB, **6**-CP-W and **8**-CP-E), assay 9 (**8**-MxnB and **6**-CP-W), and assay 10 (MxnB, **6**, and **8**). Formation of the myxopyronin derivative (**9**) was analyzed at specific time points as summarized in Fig. 4B. Most efficient production of **9** was observed in assay 7 containing **6**-MxnB and **8**-CP-E. Production yields in assay 9 (the reverse experiment) were significantly reduced and even lower than in assay 8 with non-primed MxnB. These results indicate that priming of MxnB with the western chain (**6**), which was the preferred scenario in the transacylation experiment (assay 5 *vs.* assay 6), is important for efficient product formation.

### Formation of myxopyronin side-products by MxnB

**Fig. 5:**
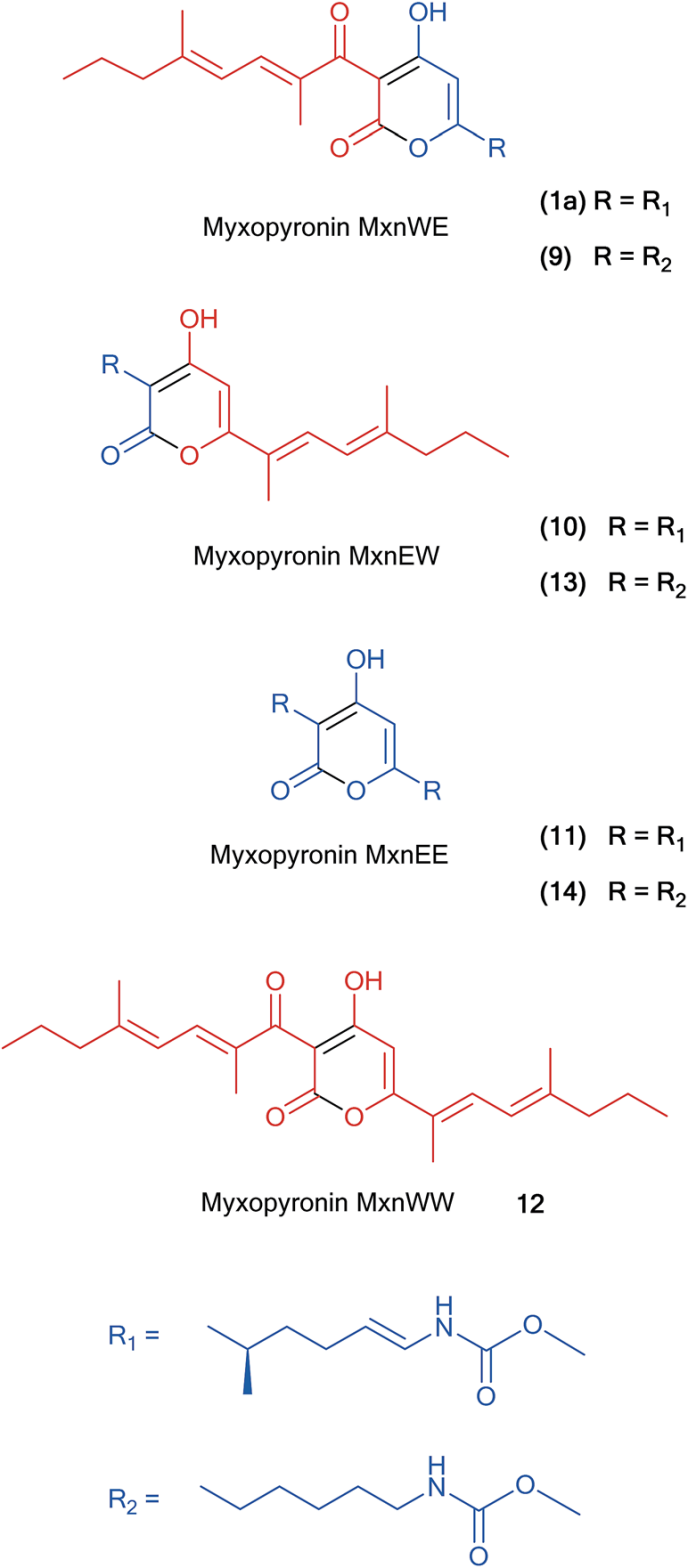
Compounds produced by the myxopyronin wild type producer and in the scope of *in vitro* assays. Red represents the western chain, blue represents the eastern chain and black represents the C–C bond formed during the condensation reaction.

Surprisingly, in addition to the major product **9** we also observed trace amounts of MxnEW (**13**) (reverse orientation), MxnEE (**14**) and MxnWW (**12**) (homo-condensation) in the *in vitro* assays (Fig. 5 and S3†). To date, myxopyronin A (**1a**) and myxopyronin B (**1b**) have been the only α-pyrone compounds reported from the native producer *M. fulvus* Mx f50.^7^ To investigate whether these side-products were an artifact of the *in vitro* assays or also produced *in vivo* we performed detailed LC-MS analysis of the *M. fulvus* Mx f50 culture extract. We detected a compound that eluted 0.8 min earlier than **1a** (MxnWE) with an *m*/*z* of 418.22 Da, suggesting the formation of **10** (MxnEW). Comparative MS^2^ fragmentation of **1a** and **10** showed three identical fragments: [M + H – H_2_O]^+^, [M + H – CH_3_OH]^+^, [M + H – H_2_N–CO_2_CH_3_]^+^ while no further related fragments were observed in **10** due to structural differences (Fig. 5 and S9†). HRMS of two additional peaks showed an [M + H]^+^ signal at *m*/*z* 451.2077 (calc. for C_22_H_30_N_2_O_8_, 451.2074) indicating the presence of MxnEE (**11**) and an [M + H]^+^ signal at *m*/*z* 385.2374 (calc. for C_24_H_33_O_4_, 385.2373), matching MxnWW (**12**) (Fig. 5 and S9†). The pathway is therefore permissive to the formation of MxnEW, -WW and -EE, even though MxnWE is the main product. Due to the small amounts of these derivatives full structure elucidation by NMR could not be performed.

The observed production of additional myxopyronin derivatives indicates that in general the biochemistry of α-pyrone ring formation tolerates a ‘switch’ of one (MxnW*W*, Mxn*E*E) or even both chains (Mxn*EW*). However, as MxnWE is clearly the dominant product the question arose whether the CPs, the substrates, or a combination of both confers specificity during the transacylation and condensation reaction.

### Biochemical insights into α-pyrone ring formation

**Fig. 6:**
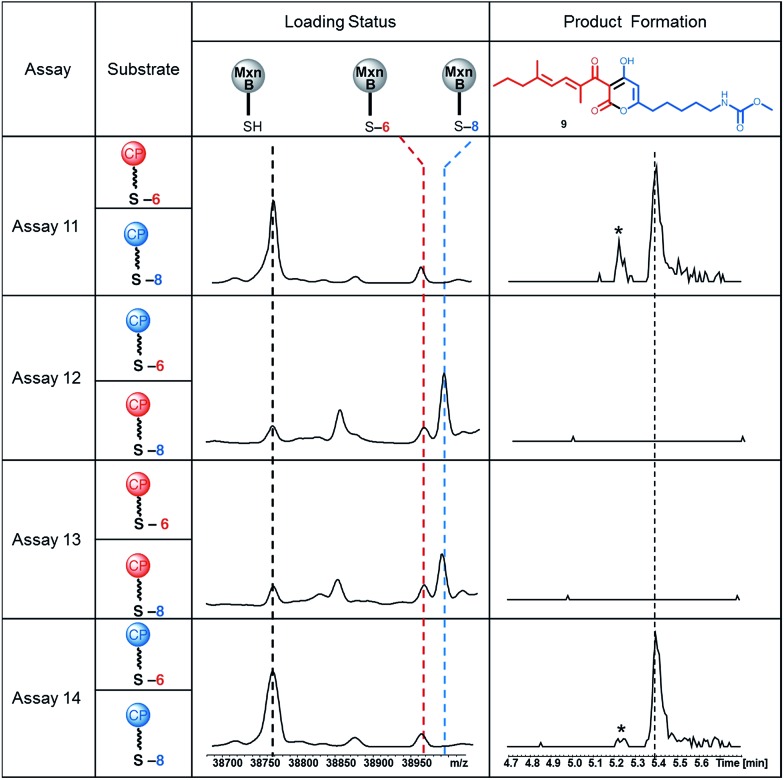
LC-MS analysis of competition assays at 1 min. Competition assay 11 (MxnB, **6**-CP-W, **8**-CP-E), assay 12 (MxnB, **8**-CP-W and **6**-CP-E), assay 13 (MxnB, **6**-CP-W, **8**-CP-W) and assay 14 (MxnB, **6**-CP-E and **8**-CP-E). Competition assays were performed in a time course of 15 s to 10 min observing transfer of substrate to MxnB (loading status). EIC of **9** at [M + H]^+^ = 406.22 for product formation are shown. Peak with asterisk (*) is the isomer of **9**.

To investigate possible routes to side-product formation we performed *in vitro* competition assays consisting of MxnB and equal amounts of CP-W and CP-E primed with either substrate **6** or **8** (Fig. S5 and S6†). Both CPs could be loaded with either substrate and displayed no obvious preference for their native cargo.^30^ As expected, rapid product formation (MxnWE) was observed in the presence of **6**-CP-W and **8**-CP-E (assay 11) (Fig. 6). We also detected a small amount of **6**-MxnB, which we assume to be the reaction intermediate. After substrate swapping to **8**-CP-W and **6**-CP-E (assay 12), MxnWE production decreased significantly (∼90-fold after 10 min) indicating that one or both CPs have an impact on the reaction sequence (Fig. 6 and S10†). In contrast to assay 11, we observed an additional, large peak corresponding to **8**-MxnB. This might be due to the formation of a ‘catalytically incompetent’ species (**8**-MxnB) as a result of fast eastern chain transfer by CP-W. A similar observation was made in assay 13 where both chains were loaded on CP-W (Fig. 6). Transacylation occurs, but only a very small amount of product is formed (∼20-fold decrease after 10 min). When both substrates are loaded onto CP-E (assay 14) we observed MxnWE production comparable to assay 11 (Fig. S10†).

Detection of side-product formation in the above assays proved difficult. Since we observed a much higher production rate of MxnWE in our preloading experiments (assay 7), we repeated them with two additional combinations. **6**-MxnB incubated with **8**-CP-E (assay 15, a repeat of assay 7) showed very fast formation of MxnWE (**9**) again. **6**-MxnB incubated with **6**-CP-W (assay 16) results in only trace amounts of MxnWW (**11**) after prolonged incubation. When **8**-MxnB is incubated with **6**-CP-W (assay 17, a repeat of assay 9), we could not observe product formation of the expected MxnEW (**13**). Finally, we tested **8**-MxnB with **8**-CP-E (assay 18) and observed rapid formation of MxnEE (**14**) (Fig. S11†).

Overall, these data indicate that substrate transfer (transacylation) by CP-W is rapid and highly specific. In contrast, the results demonstrate that CP-E is able to efficiently mediate both the transacylation and the condensation reaction, which may be the result of a less specific substrate transfer. On the substrate side the western chain appears to be more readily transferred to MxnB but we cannot exclude that use of the native eastern chain could alter this observation. We believe that a combination of both CP and substrate confers specificity for α-pyrone ring formation.

The complexity of the MxnB catalyzed condensation reaction involving CPs and small molecule specificities render a general analysis of substrate specificity *in vitro* difficult. However, we recently indirectly addressed this question *in vivo* in a mutasynthesis study employing a *M. fulvus* mutant unable to biosynthesize the western chain. This study revealed that MxnB is capable of condensing a wide variety of activated synthetic western chains with the CP bound native eastern chain.^31^ We used a set of substrate combinations from the mutasynthesis experiments *in vitro*, including those that did not give product *in vivo*. These additional *in vitro* assays (Fig. S12†) show a high tolerance of MxnB for non-native NAC substrates. Even substrates lacking the α-methyl group and the α,β-double bond of the western chain, which did not give product *in vivo*, were condensed *in vitro* to produce the expected analogues. It is not clear whether the differences between *in vivo* and *in vitro* experiments are due to difficulties in incorporating the non-native analogs into the biosynthetic pathway, or a result of relaxed enzyme specificity in the *in vitro* system. Due to the limited availability of substrates as well as the overall complexity of the reaction we did not attempt further analyses.

### Mechanism proposal

The MxnB structural data suggested that binding of substrate in the red tunnel precedes binding in the blue tunnel. In agreement with the biochemical data, we propose that the western chain is preferentially delivered to and/or bound in the red tunnel, while the eastern chain is preferentially supplied *via* the blue tunnel. We sought to investigate the existence of the blue tunnel by blocking it *via* mutagenesis. However, the half of the blue tunnel proximal to the catalytic cysteine is not trivial to block by mutagenesis. It consists of two helices engaged in complex hydrophobic interactions with each other and a central β-sheet. Only one residue (T322) offers a side-chain orientation suitable for a straightforward mutation (*i.e.* not contacting other residues). Unfortunately, the candidate residue T322 is close to the junction connecting both tunnels. Therefore mutations are likely to affect binding of chains in both tunnels. In line with this hypothesis mutation of T322 to Leu negatively affected loading of the eastern chain almost twice as much as that of the western chain, but also led to a 7-fold reduction in enzyme activity (Fig. S13, Table S7†).

**Scheme 2:**
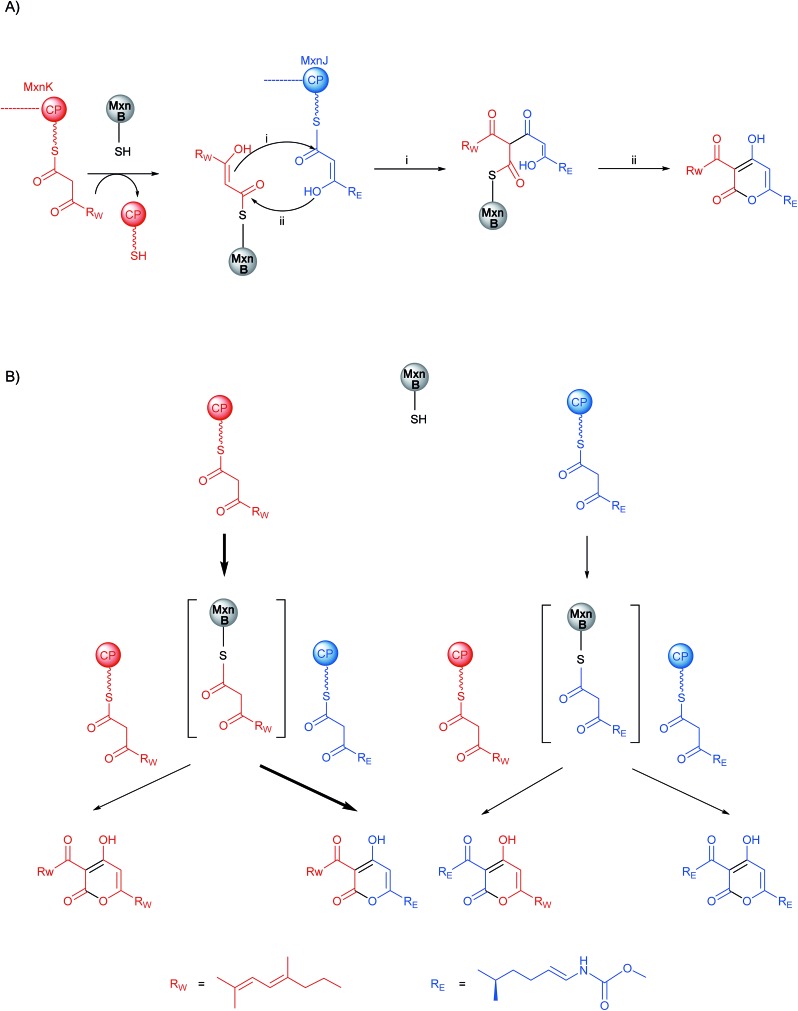
(A) Proposed mechanism for α-pyrone ring formation in myxopyronin biosynthesis; (B) a flow chart illustrating the reactions that occur during myxopyronin production *in vitro* and *in vivo*. Bold arrow represents the preferred product formation.

Based on the series of assays described above, we propose the mechanism of pyrone ring formation in myxopyronin as follows: first, CP-W interacts with MxnB and transfers its bound polyketide chain to the MxnB active-site cysteine (C121) (Scheme 2A). CP-E delivers/provides the condensation partner (eastern chain) to the western-chain primed MxnB. Pyrone ring formation results from nucleophilic attack of the α,β-enol-thioester of the KS-bound western chain on the thioester carbonyl of CP-E bound eastern chain to form a diketothioester *via* a C–C bond formation (i). Subsequently lactonization *via* an enolate intermediate of the eastern chain (ii) occurs to form a C–O bond (Scheme 2A). Although we have no experimental evidence for the timing of the two condensation reactions (i and ii) we consider it more likely that C–C bond formation happens first. Following the same proposed mechanism, all four products, MxnWE and MxnEW, as well as MxnWW and MxnEE could be formed (Scheme 2B). Due to the complexity of the reaction cascade kinetic parameters could not be determined in this study.

An intriguing mechanism proposal was very recently reported for CsyB.^26^ This enzyme is a fungal type III PKS involved in acylalkylpyrone (AcAP) formation. In the CsyB catalyzed reaction, acetoacetyl-CoA is loaded onto the active site cysteine C155 and the thioester subsequently undergoes hydrolysis by a water molecule activated through hydrogen bonding to C155 and H377, generating the β-keto acid intermediate. This intermediate was proposed to move to a position of CsyB corresponding to the entrance of the blue tunnel in MxnB. Once the β-keto acid intermediate is relocated, a second reaction cycle occurs: the enzyme is loaded with a fatty acid, which is elongated with one molecule of malonyl-CoA. This β-ketoacyl unit then reacts with the β-keto acid giving rise to AcAP (Fig. S14†). A series of *in vitro* assays using H_2_^18^O supported thioester bond cleavage, as this mechanism explains the enzymatic incorporation of one ^18^O atom into the product. A similarly positioned water molecule hydrogen bonded to C121 and S324 is observed in the MxnB structure. To investigate whether a similar mechanism exists in the MxnB catalyzed reaction, we performed *in vitro* assays in a similar manner to the work of Mori and coworkers.^26^ After quenching of the reaction with iodoacetamide, we could not observe significant incorporation of ^18^O into **9** (Table S9†). Thus, in myxopyronin biosynthesis two β-ketoacyl chains are condensed to form the α-pyrone ring without generating a β-ketoacid intermediate through hydrolysis. We therefore hypothesize that the MxnB catalyzed reaction proceeds *via* a different mechanism than described for CsyB.

## Conclusions

The insights gained from this study enable us to propose a biochemical mechanism for α-pyrone ring formation in myxopyronin biosynthesis. It should be noted that other biochemically characterized ketosynthases related to ring formation and condensation of two substrates such as RkD and CsyB significantly differ from MxnB. RkD is a type I PKS ketosynthase that condenses CP-bound substrates forming the tetronate ring in RK-682 biosynthesis.^17^ Furthermore, CsyB represents a type III PKS from *Aspergillus oryzae* that condenses two β-ketoacyl-CoAs to form an α-pyrone ring.^16^ Through biochemical and crystallographic studies, we elucidated the reaction mechanism of MxnB as catalyzing α-pyrone formation and thus established MxnB as a novel member of the ketosynthase family. The unique biochemical features of MxnB might also be used as a starting point to expand the variety of α-pyrone compounds formed by *in vitro* and *in vivo* reactions.

## Supplementary Material

Supplementary information
