# Supplementary material for: In vitro reconstitution of α-pyrone ring formation in myxopyronin biosynthesis
Source: Chem Sci. 2015 May 18;6(8):5076–85. doi: 10.1039/c5sc01013f (PMC5724707; doi:10.1039/c5sc01013f)
Supplement: Supplementary file 1 [file SC-006-C5SC01013F-s001.pdf]

Electronic Supplementary Material (ESI) for Chemical Science.
This journal is © The Royal Society of Chemistry 2015

Supporting Information

In Vitro Reconstitution of α-Pyrone Ring Formation in Myxopyronin Biosynthesis

Hilda Sucipto[a], Jan Hennning Sahner[b], Evgeny Prusov[c], Silke C. Wenzel[a], Rolf W. Hartmann[b], Jesko Koehnke[d], and Rolf Müller[a]

[a] Department of Microbial Natural Products, Helmholtz Institute for Pharmaceutical Research Saarland and Pharmaceutical Biotechnology, Saarland University, University Campus Building C2 3, 66123 Saarbrücken, Germany

[b] Department of Drug Design and Optimization, Helmholtz Institute for Pharmaceutical Research Saarland and Pharmaceutical and Medicinal Chemistry, Saarland University, University Campus Building C2 3, 66123 Saarbrücken, Germany

[c] Helmholtz Centre for Infection Research (HZI), Inhoffenstrasse 7, 38124 Braunschweig, Germany

[d] Workgroup Structural Biology of Biosynthetic Enzymes, Helmholtz Institute for Pharmaceutical Research Saarland, Saarland University, University Campus Building C2 2, 66123 Saarbrücken, Germany

Contents of Supporting Information

Experimental Procedures 3

Supplementary Results 3

Table S1. List of strains and plasmids used in this study 9

Table S2. Blast Search of MxnB 10

Table S3. NMR spectroscopic data for myxopyronin derivative **9** 14

Table S4. ESI-MS analysis of the recombinant proteins 20

Table S5. MS analysis of the loading of **6** or **8** onto MxnB, CP-E and CP-W 20

Table S6. Characterization of myxopyronin A and its analogues with representative MS^2 fragmentation 25

Table S7. MS analysis of the loading of **6** or **8** onto MxnB and MxnB mutant T322L 31

Table S8. Data collection and refinement statistics for the MxnB crystal structure 32

Table S9. ^{18}O incorporation into myxopyronin derivative **9** 33

Figure S1. Phylogram of ketosynthases from various pathways 11

Figure S2. ESI-MS analysis of myxopyronin A produced *in vitro* and LC-MS analysis for the in vitro formation of MxnB with **7** or **8** 13

Figure S3. ESI-MS Analysis of myxopyronin derivatives produced *in vitro* 15

Figure S4. NMR spectra for myxopyronin derivative **9** 16

Figure S5. LC-MS analysis of the formation of **6**-*S*-CP-W and **8**-*S*-CP-W 21

Figure S6. LC-MS analysis of the formation of **6**-*S*-CP-E and **8**-*S*-CP-E 22

Figure S7. Superposition of MxnB and OleA 23

Figure S8. LC-MS analysis of the formation of **6**-MxnB and **8**-MxnB 24

Figure S9. ESI-MS analysis of myxopyronin and its derivatives produced *in vivo* 26

Figure S10. LC-MS analysis for Assays 11-14 27

Figure S11. LC-MS analysis for Assays 15-18 28

Figure S12. ESI-MS analysis of other myxopyronin derivatives produced *in vitro* 29

Figure S13. LC-MS analysis of the *in vitro* Assay using MxnB mutant T322L 31

Figure S14. Comparison between proposed mechanisms of CsyB and MxnB 34

Supplementary Materials and Methods for Synthesis Procedures 35

References 57

Experimental Procedures

Materials. The PCR reactions were carried out using Phusion or Taq DNA polymerase (Fermentas) in a peqSTAR 96 Universal Gradient thermocycler (Peqlab): Initial denaturation (3 min, 95 °C); 30 cycles of denaturation (30 s, 95 °C), annealing (30 s, 53 or 57 °C) and elongation (varied based on PCR product length 1 kb/min, 72 °C); and final extension (10 min, 72 °C). DNA fragments were separated by agarose gel electrophoresis and isolated using the peqGold Gel Extraction (Peqlab). The PCR products were cloned into the pJET1.2 blunt (Thermo Fisher Scientific) vector and sequenced using the primers pJET1.2For/pJET1.2Rev.

Cloning of MxnB. *mxnB* was amplified from *M. fulvus* Mx f50 genomic DNA by PCR using primers mxn9: 5'-GTC AGA CAT ATG AAC AAC AGC GGT-3' (*Nde*I site is underlined) and mxn10: 5'- CGT CGT AAG CTT TCA GTA GGT GAA AAC CA-3' (*Hind*III site is underlined). The PCR products were subcloned into the pJET1.2 blunt vector resulting in pJET-HSU-mxn5 and sequenced. pJET-HSU-mxn5 was digested with *Nde*I and *Hind*III and the DNA fragment harboring the MxnB encoding region was cloned into the *Nde*I and *Hind*III site of pColdI resulting in pColdI-HSU-mxn5 encoding MxnB with an N-terminal hexahistidine tag. All strains and plasmids used in this study are listed in Table S1.

Mutagenesis of MxnB. MxnB mutants was generated by adapting the method from the QuikChange II XL Site-Directed Mutagenesis (Stratagene) and pColdI-HSU-mxn5 as the template. For the C121A mutation, primers mxn236: 5'-CCATCACCGTCAACGCCTCCGCCCTGAGTTTCTTCGTCGC-3' and mxn237: 5'-GCGACGAAGAAACTCAGGGCGGAGGCGTTGACGGTGATGG-3' (mutated nucleotides are underlined) were used. For the T322L mutation, primers mxn296: 5'-GCCGTGCTGACCGGCCTGGGCTCCGGAGTCTCCTTCGTGG-3' and mxn297:5'-CCACGAAGGAGACTCCGGAGCCCAGGCCGGTCAGCACGGC-3'. The primers were used to amplify the template to obtain pColdI-MxnB-C121A, pColdI-mxnB-T322L. The clones were verified by sequencing.

Cloning of CP-E6 and co-expression with MtaA. *CP-E6* was amplified from *M. fulvus* Mx f50 genomic DNA by PCR using the primers mxn214: 5'-GAT ACG CAT ATG GTC CCC TTC GAG TCG AGC-3' (*Nde*I site is underlined) and mxn215: 5'-TCT ATG AAG CTT TCA TGG CTT CGC TCC CGC-3' (*Hind*III site is underlined). The PCR products were subcloned into the pJET1.2 blunt vector resulting in pJET-HSU-mxn59 and sequenced. pJET-HSU-mxn59 was digested with

*Nde*I and *Hind*III and the DNA fragment harboring the CP-E6 encoding region was cloned into the *Nde*I and *Hind*III site of pColdI resulting in pColdI-HSU-mxn59 (CP-E6) encoding CP-E6 with an N-terminal hexahistidine tag. Consequently, pColdI-HSU-mxn59 and pSUMtaA were co-transformed. Subsequent co-expression enabled phosphopantetheinylation of apo-CP-E6 *in vivo* forming holo-CP-E6.

Cloning of CP-W5 and co-expression with MtaA. *CP-W5* was amplified from *M. fulvus* Mx f50 genomic DNA by PCR using the primers mxn230: 5'-CTG AGG CAT ATG CGT GCG TTC GAG TCG-3' (*Nde*I site is underlined) and mxn231: 5'- TCT ATG AAG CTT TCA GGC ACA CCA CTC ATT-3' (*Hind*III site is underlined). The PCR products were cloned into the pJET1.2 blunt vector resulting in pJET-HSU-mxn63 and sequenced. pJET-HSU-mxn63 was digested with *Nde*I and *Hind*III and the DNA fragment harboring the CP-W5 encoding region was cloned into the *Nde*I and *Hind*III site of pColdI resulting in pColdI-HSU-mxn63 (CP-W5) encoding CP-W5 with an N-terminal hexahistidine tag. Furthermore, pColdI-HSU-mxn63 was co-transformed with pSUMtaA to obtain a strain co-expressing pColdI-HSU-mxn63 and pSUMtaA. This co-expression enabled phosphopantetheinylation of apo-CP-W5 *in vivo* forming holo-CP-W5.

Expression and purification of proteins. For expression and purification of MxnB, MxnB[#] (C121A), Mxn104 (T322L), holo-CP-E6 and holo-CP-W5, the expression plasmids pHSU-mxn5, pHSU-mxnB[#], pHSU-mxn104, respectively, were introduced into *Escherichia coli* BL21(DE3). For co-expression of pColdI-HSU-mxn59 and pSUMtaA as well as coexpression of pColdI-HSU-mxn63 and pSUMtaA the respective plasmids were introduced into *Escherichia coli* BL21(DE3). The *E. coli* BL21(DE3) strains carrying the expression plasmids were cultured in LB medium containing 100 μg/mL of ampicillin or with additional 25 μg/mL chloroamphenicol for co-expression with MtaA at 37°C until they reached an OD_{600} = 0.5 – 0.6. Expression was induced by addition of isopropyl-β-D-thiogalactopyranoside (IPTG, 0.15 mM) and cultivation was continued at 16°C for 20 h. The cells were harvested by centrifugation (8,000 rpm; 20 min) and resuspended in lysis buffer (40 mM Tris-HCl buffer pH 7.5 containing glycerol 10%, 300 mM NaCl and 10 mM imidazole). Cells were lysed by sonication and a cell-free extract was prepared by removing cell debris by centrifugation at 8,000 rpm for 10 min at 4°C. The cell-free extract was purified by using Ni-NTA Superflow resin (Qiagen), washed sequentially with buffer containing imidazole (10 mM and 20 mM), and eluted with high imidazole (100 mM for CPs and 200 mM for MxnB; MxnB[#]) in the same buffer. The elution buffer was exchanged with buffer (40 mM Tris-HCl buffer pH 7.5 containing glycerol 10%, 300 mM NaCl) using Amicon Ultra 10-k or

30-k (Milipore) to remove imidazole and also to concentrate the proteins. Protein concentrations were estimated based on Bradford assays (Bio-Rad) employing bovine serum albumin as a standard.[1] The purified proteins were flash frozen in liquid nitrogen and stored at -80°C until further use. The recombinant protein masses are listed in Table S4.

Expression and purification for crystallography. Full-length *mxnB* with an N-terminal His_6-tag was cloned into the pCold1 plasmid. The protein was expressed in *Escherichia coli* BL21 (DE3) grown on Lysogeny broth. Cells were grown at 37 °C to an OD_{600} = 0.6, cultures subjected to a cold-shock for 30 min and expression induced by addition of 0.1 mM IPTG. Cells were harvested after 16 h growth at 16 °C by centrifugation at 4,000 x g, 4 °C for 15 min and re-suspended in lysis buffer (200 mM NaCl, 20 mM Tris pH 8.5, 20 mM imidazole, 10 % glycerol and 3 mM β-mercaptoethanol (BME)) with the addition of complete EDTA-free protease inhibitor tablets (Roche) and 0.4 mg DNase g^{-1} wet cells (Sigma). Cells were lysed by passage through a cell disruptor at 30 kPSI (Constant Systems Ltd) and the lysate was cleared by centrifugation at 40,000 x g, 4 °C for 20 min. The cleared lysate was applied to a Nickel Sepharose 6 Fast Flow (GE Healthcare) column pre-washed with lysis buffer and protein eluted with 250 mM imidazole in the same buffer. The protein was then subjected to size-exclusion chromatography (Superdex™ 200, GE Healthcare) in 200 mM NaCl, 10 mM Tris pH 8.5, 1 mM TCEP, 10 % glycerol and concentrated to 4.5 mg mL^{-1}.

Crystallization, data collection, and crystallographic analysis. Crystallization trials of MxnB were set up at 4.5 mg ml^{-1}. MxnB crystals were obtained in 0.2 M Ammonium phosphate, 0.1 M Tris pH 8.5 and 50 % MPD. The crystals did not need additional cryoprotection and were flash-cooled in liquid nitrogen. The crystals belonged to space group P1 with cell dimensions a = 50.19 Å b = 58.79 Å, c = 62.82 Å, α = 104.75, β = 105.48 and γ = 93.90. Diffraction data was collected at ESRF beamline ID29 at 100 K and processed with xia2. The structure of MxnB was determined by molecular replacement using Phaser[2,3] with the Crystal structure of 3-oxoacyl-[acyl-carrier-protein] synthase III from Aquifex aeolicus VF5 as a search model (PDB: 2EBD). Complete manual rebuilding was performed with COOT[4] and refinement was performed using CCP4 REFMAC5[5] and Phenix Refine.[6] The statistics of data collection and refinement are summarized in Table S4. All molecular graphics figures were generated with the program Pymol.[7]

***In vitro* reconstitution of myxopyronin (or derivatives) formation.** The one pot reaction mixture in 40 mM Tris-HCl buffer pH 7.5, glycerol 10%, 300 mM NaCl (100 μL) containing 100

μM CP-W5, 100 μM CP-E6, 30 μM MxnB and 1 mM substrates (**6** with **7** or **6** with **8** or **S1**-**S4** with **8**) was incubated at 37 °C for 2 h. Reaction mixtures were extracted with ethyl acetate and the organic layer was evaporated. The residue was dissolved in 100 μl methanol and a 5 μl aliquot of the extract was analyzed by HPLC-MS.

Isolation of *in vitro* product. Large scale of *in vitro* assay containing 100 μM CP-W5, 100 μM CP-E6, 30 μM MxnB and 0.5 mM **6** and **8** was incubated in 40 mM Tris-HCl buffer pH 7.5, glycerol 10%, 300 mM NaCl (total 9 mL) for 9 h at 37 °C. Reaction mixtures were extracted with ethyl acetate and the organic layer was concentrated *in vacuo*. The crude extract was subsequently purified by semi preparative reverse-phase HPLC (Phenomenex Jupiter® 4 μm Proteo 90 Å, C12, 250 x 10 mm, DAD at 220 and 310 nm). Separation was achieved by a linear gradient of 10% ACN + 0.1 % FA for 6 min, 10 % to 85 % ACN + 0.1 % FA in 4 min, 85% to 100% ACN + 0.1 % FA in 20 min at a flow rate of 2.5 ml min^{-1} and 30 °C of column temperature to afford semipure **9** (0.3 mg, t_R = 17.5 min). A full set of NMR spectra was recorded with the semipure fraction (due to low quantity) before final purification was achieved. NMR data of semipure and pure compound (0.1 mg) were acquired in CD_3OD on a 700 MHz *Avance III* (Ascend) spectrometer by *Bruker BioSpin GmbH*, equipped with a 5mm TXI cryoprobe, at 298 K.

***In vitro* self-acylation of the CPs and MxnB.** A reaction mixture containing 100 μM of CP-W5 or 100 μM of CP-E6 or 30 μM of MxnB with 1 mM of **6** (JHS486) or **8** (JHS274) was incubated at 37°C for 2 h in 40 mM Tris-HCl buffer pH 7.5, glycerol 10%, 300 mM NaCl to obtain **6**-CP-W, **8**-CP-W, **6**-CP-E, **8**-CP-E**,** **6**-MxnB, and **8**-MxnB. For the purpose of competition assays, the leftover substrates were removed by gel filtration using a PD-10 column and further concentrated with Amicon Ultra 10-K or 30-K concentrators as described above. Substrate transfer to CPs was confirmed by LC-ESI-MS and the percentage of loaded-substrate CPs were calculated using the deconvoluted mass peak heights.

Assays 5-6 and Assays 15-18. The reaction master mixture in the same buffer as above (225 μL) containing 3.76 μM of loaded CPs final concentration as produced in the assay above was aliquoted to 10 eppendorf tubes which were kept on ice. In these assays, we keep the ratio between the non-acylated and acylated CPs constant. When the experiment was started, the eppendorf tube was incubated at 30 °C for 1 min before addition of MxnB (2.3 μM a final concentration). The reaction was stopped by putting the tubes into liquid N_2 at time points: 15 s, 30 s, 1 min, 2 min, 5 min, 10 min. Liquid N_2 was chosen as a way to avoid MxnB precipitation.

One part of the sample was used for protein analysis and another part of the sample was quenched by the addition of ice-cold 40% MeOH for product analysis. All sample preparations were performed in the cold room and the autosampler of the HPLC was kept at 5 °C to avoid occurrence of any further reactions.

Competition Assays 7-10 and Assays 11-14. The reaction master mixture in the same buffer as above (200 μL) containing 4 μM of loaded CPs and 4 μM of loaded MxnB was incubated at 30 °C, and samples of 20 μL were taken at time points: 15 s, 30 s, 1 min, 2 min, 5 min, 10 min. In these assays, we keep the ratio between the non-acylated and acylated CPs constant.For assay 10, 14.81 μM of MxnB was and 0.5 mM of **6** or **8** was used. The reaction was quenched by the addition of 30 μL ice-cold 40% MeOH. The samples were then measured by HPLC-MS for product detection.

$H_2^{18}O$ water experiments. A 50 μl reaction mixture in 40 mM Tris-HCl buffer pH 7.5 containing 2 μM of loaded CPs and 7.5 μM of MxnB in 75% $H_2^{18}O$ was incubated at 37 °C for 2 h. The mixture was then quenched with iodoacetamide (final 1 mM). After centrifugation at 15,000 rpm for 2 min, the supernatant was divided into two aliquots. One volume of MeOH was added to the first aliquot for LCMS measurement. The second aliquot was dried using a Genevac evaporator and dissolved in $H_2^{16}O$ for 5 h further incubation. One volume of MeOH was added to the aliquot for LCMS measurement.

LC-ESI-MS measurements for protein analysis. All ESI-MS-measurements for intact proteins were performed on a Dionex Ultimate 3000 RSLC system using an Aeris Widepore XB-C8, 150 x 2.1 mm, 3.6 µm dp column (Phenomenex, USA). Separation of 1 µl sample was achieved using a linear gradient from (A) H_2O + 0.05 % FA to (B) ACN + 0.05 % FA at a flow rate of 300 µl min^{-1} and 45 °C. The gradient was initiated by a 1.0 min isocratic step at 2 % B, followed by an increase to 60 % B at 8 min to end up with a 3 min step at 60 % B before reequilibration in initial conditions. UV spectra were recorded with a DAD in the range from 200 to 600 nm. The LC flow was split to 75 µl/min before entering the maXis 4G hr-ToF mass spectrometer (Bruker Daltonics, Bremen, Germany) using the standard Bruker ESI source. In the source region, the temperature was set to 180 °C, the capillary voltage was 4000 V, the dry-gas flow was 6.0 l/min and the nebulizer was set to 1.1 bar. Mass spectra were acquired in positive ionization mode ranging from 600 – 1800 m/z at 2.5 Hz scan rate. Protein masses were deconvoluted by using the Maximum Entropy algorithm (Copyright 1991-2004 Spectrum Square Associates, Inc.).

HPLC-MS measurements to analyze the products of *in vitro* assays. High Performance Liquid Chromatography-Mass Spectrometry (HPLC-MS) (Thermo Ultimate 3000 RSLC, coupled to a Bruker Daltonics amaZon Electrospray Ionization (ESI)-MS ion trap instrument) was operated in positive ionization mode. Compounds were separated on a Waters Acquity BEH C18 column (50 x 2.1 mm; 1.7 µm particle diameter) at a flow rate of 600 µl min^{-1} and 45 °C by a linear gradient with (A) H_2O + 0.1 % formic acid (FA) to (B) acetonitrile (ACN) + 0.1 % FA at a flow rate of 600 µl min^{-1} and 45 °C. The gradient was initiated by a 0.33 min isocratic step at 5 % B, followed by an increase to 95 % B in 9 min to end up with a 1 min flush step at 95 % B before re-equilibration with initial conditions. Detection was carried out by both diode array (DAD) and ESI-MS. For high-resolution mass spectrometry analysis, measurements were performed on a Dionex Ultimate 3000 RSLC system using a Waters BEH C18 column (50 x 2.1 mm, 1.7 µm dp) by injecting 5 µl of the methanolic extract. Separation was achieved using the same gradient as above with a 0.33 min isocratic step at 5 % B. UV and MS detection were performed simultaneously. Coupling the HPLC to the MS was supported by an Advion Triversa Nanomate nano-ESI system attached to a Thermo Fisher Orbitrap. Mass spectra were acquired in centroid mode ranging from 200-2000 m/z at a resolution of R = 30000.

HPLC-MS measurements to analyze the products of *in vitro* competition assays. All measurements were performed on a Dionex Ultimate 3000 RSLC system using a Waters BEH C18, 50 x 2.1 mm, 1.7 µm dp column. Separation of 10-15 µl sample was achieved by a linear gradient with (A) H_2O + 0.1 % FA to (B) ACN + 0.1 % FA at a flow rate of 600 µl/min and 45 °C. The gradient was initiated by a 1 min isocratic step at 5 % B, followed by an increase to 95 % B in 6 min to end up with a 1.5 min step at 95 % B before reequilibration with initial conditions. UV spectra were recorded by a DAD in the range from 200 to 600 nm. The LC flow was split to 75 µl/min before entering the maXis 4G hr-ToF mass spectrometer (Bruker Daltonics, Bremen, Germany) using the standard ESI source. Mass spectra were acquired in centroid mode ranging from 50 – 1000 m/z at 2 Hz scan speed.

Table S1. List of strains and plasmids used in this study

Strain/Plasmid	Characteristics	Reference
***E.coli* strains**		
HS996	Host for general cloning	Invitrogen
BL21 (DE3)	Host for protein expression	Invitrogen
***Myxococcus fulvus* strains**		
Mx f50	Myxopyronin producing wild-type strain	[7]
**Plasmids**		
pColdI	E. coli protein expression vector	Takara
pSUMtaA	containing a 4'-phosphopantetheinyl transferase *sfp* gene	[8]
pHSU-mxn5 (pColdI-MxnB)	MxnB protein expression construct with N-terminal His-tag based on pColdI	this study
pHSU-mxn5# (pColdI-MxnB#)	MxnB mutant (C121A) protein expression construct with N-terminal His-tag based on pColdI	this study
pHSU-mxn104 (pColdI-MxnB-T322L)	MxnB mutant (T322L) protein expression construct with N-terminal His-tag based on pColdI	this study
pHSU-mxn68 (pColdI-CP-W5-MtaA)	CP-E6 protein expression construct with N-terminal His-tag based on pColdI co-expressed with pSUMtaA	this study
pHSU-mxn69 (pColdI-CP-E6-MtaA)	CP-W5 protein expression construct with N-terminal His-tag based on pColdI co-expressed with pSUMtaA	this study

Supplementary Results

Table S2. Blast search of MxnB. First 20 hits of the BLAST search of MxnB from the NCBI database (non-redundant) sorted by their E-values (as per 09 March 2015).

Accession Number	Description	E Value
AGS77282.1	ketosynthase [Myxococcus fulvus]	0.0
ADI59524.1	CorB [Corallococcus coralloides]	0.0
WP_012507654.1	3-oxoacyl-ACP synthase [Pelodictyon phaeoclathratiforme]	8e-97
WP_006366404.1	3-oxoacyl-ACP synthase [Chlorobium ferrooxidans]	2e-91
WP_036102127.1	3-oxoacyl-ACP synthase [Lysobacter capsici]	1e-90
WP_025696271.1	3-oxoacyl-ACP synthase [Paenibacillus forsythiae]	2e-85
WP_026559228.1	3-oxoacyl-ACP synthase [Bacillus sp. J37]	5e-84
WP_000833196.1	3-oxoacyl-ACP synthase [Bacillus cereus]	7e-83
WP_000833203.1	3-oxoacyl-ACP synthase [Bacillus cereus]	3e-82
WP_020062449.1	3-oxoacyl-ACP synthase [Bacillus sp. 123MFChir2]	3e-82
WP_001989055.1	3-oxoacyl-ACP synthase [Bacillus cereus]	4e-82
WP_000833206.1	3-oxoacyl-ACP synthase [Bacillus cereus]	4e-82
NP_845551.1	putative 3-oxoacyl-ACP synthase III [Bacillus anthracis str. Ames]	4e-82
WP_000833198.1	MULTISPECIES: 3-oxoacyl-ACP synthase [Bacillus cereus group]	6e-82
WP_029438921.1	3-oxoacyl-ACP synthase [Bacillus thuringiensis]	7e-82
YP_037326.1	3-oxoacyl-ACP synthase [Bacillus thuringiensis serovar konkukian str.97-27]	7e-82
WP_016083203.1	3-oxoacyl-[acyl-carrier-protein] synthase 3 [Bacillus cereus]	1e-81
WP_000833202.1	3-oxoacyl-ACP synthase [Bacillus thuringiensis]	2e-81
KGZ76433.1	3-oxoacyl-ACP synthase [Bacillus anthracis]	2e-81
WP_000833204.1	3-oxoacyl-[acyl-carrier-protein] synthase 3 [Bacillus cereus]	2e-81

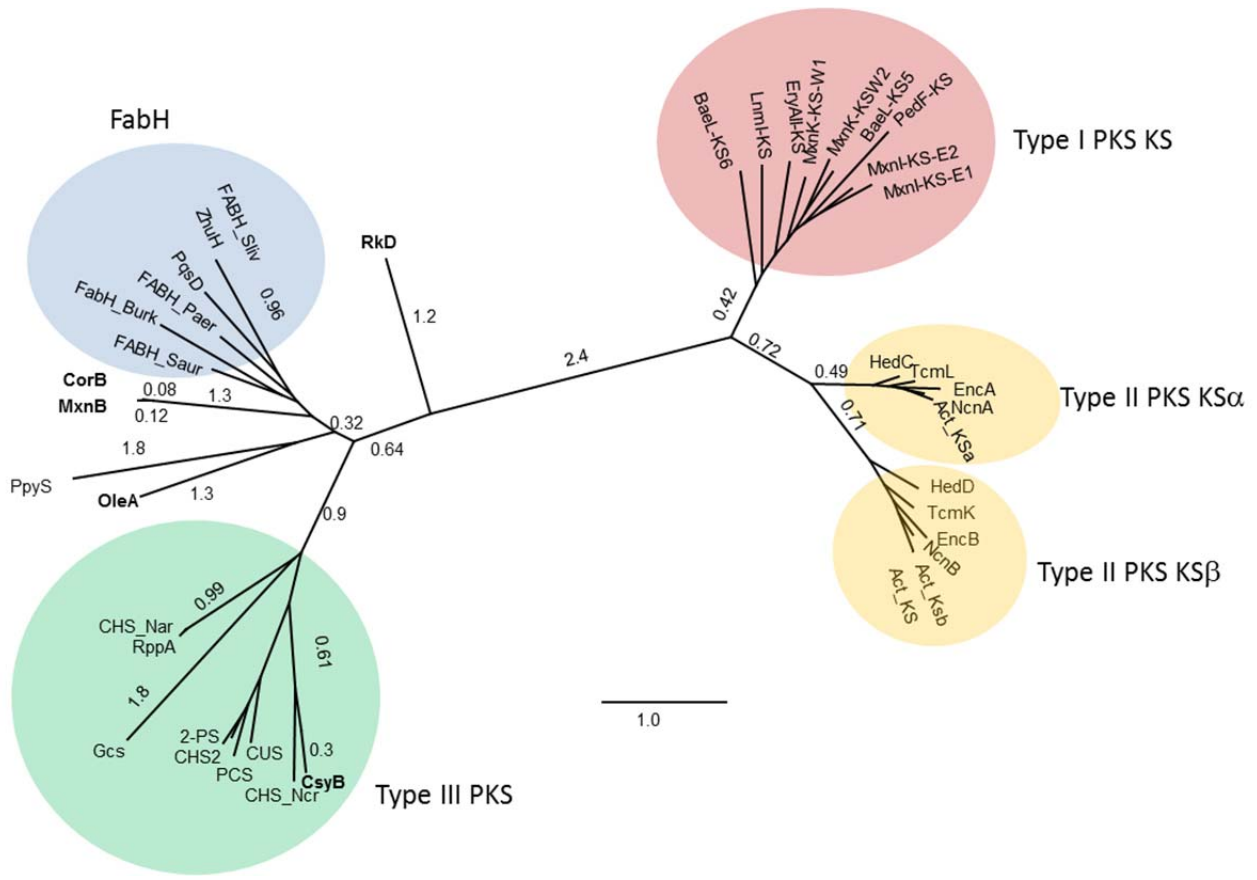

Figure S1. Phylogram of ketosynthases from various pathways. Protein sequences were aligned using ClustalW. Phylogenetic analysis was performed with a maximum-likelihood estimation approach (Whelan-and-Goldman 2001) in Geneious 8.1.4. Tip labels reflect the protein names. The proteins explained in the main text are marked bold. The scale bar indicates the degree of divergence as substitutions per sequence position. The following list comprises the locus name, the organism, and the access number of all given proteins: 2-PS (*Gerbera hybrida*, CAA86219), Act_KS (*Streptomyces lividans*, WP_003973890), Act_KSa (*S. coelicolor* A3(2),CAC44200), Act_KSb (*S. coelicolor* A3(2),CAC44201), BaeL-KS5 (*Bacillus amyloliquefaciens* subsp. plantarum AS43.3, WP_040238120.1), BaeL-KS6 (*Bacillus amyloliquefaciens* subsp. plantarum AS43.3, WP_040238120.1), CHS2 (*Medicago sativa*, P30074), CHS_Nar (*S. fulvissimus* DSM 40593, AGK75504), CHS_Ncr (*Neurospora crassa* OR74A, XP_960427), CorB (*Corallococcus coralloides*, ADI59524), CsyB (*Aspergillus oryzae*, BAD97391), CUS (*O. sativa* Japonica, Q8LIL0), EncA (*S. maritimus*, AAF81728), EncA (*S. maritimus*, AAF81729), EryAII-KS (*Sacchropolyspora erythraea* NRRL 2338, CAA44448.1), FabH_Burk (*Burkholderia xenovorans,* WP_011490245), FabH_Paer (Pseudomonas aeruginosa PAO1, Q9HYR2), FabH_Saur (*Staphylococcus aureus*, Q8NXE2), FABH_Sliv (*S. lividans*, Q9F6D4), Gcs (*S. coelicolor*, WP_011031521), HedC (*S. griseoruber*, AAP85362), HedD (*S. griseoruber*, AAP85361), LnmI-KS (*S. atroolivaceus*, AAN85522.1), MxnB (*Myxococcus fulvus* Mx f50, AGS77282.1), MxnI-KS-E1 (*M. fulvus* Mx f50, AGS77289.1), MxnI-KS-E2 (*M. fulvus* Mx f50, AGS77289.1), MxnK-KS-W1 (*M. fulvus* Mx f50, AGS77291.1), MxnK-

KS-W2 (*M. fulvus* Mx f50, AGS77291.1), NcnA (*S. arenae*, AAD20267), NcnB (*S. arenae*, AAD20268), OleA (*Xanthomonas campestris*, WP_011035468), PCS (*Aloe arborescens*, Q58VP7), PedF-KS (symbiont bacterium of *Paederus fuscipes*, AAS47564.1), PpyS (*Photorhabdus luminescens*, AGO97060), PqsD (*P. aeruginosa*, P20582), RkD (*Streptomyces* sp. 88-682, ACZ65477), RppA (*S. griseus*, BAA33495), TcmK (*S. davawensis*, WP_015657287), TcmL (*S. davawensis*, WP_015657288), ZhuH (*Streptomyces* sp. R1128, AAG30195).

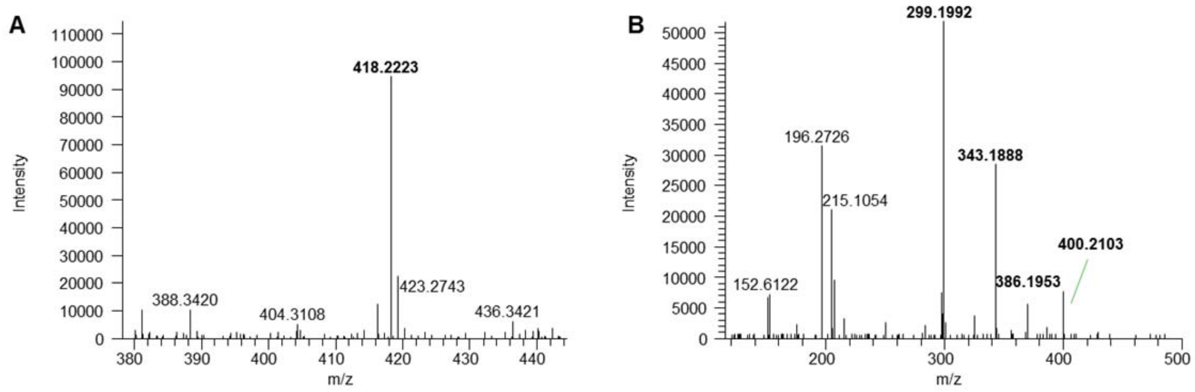

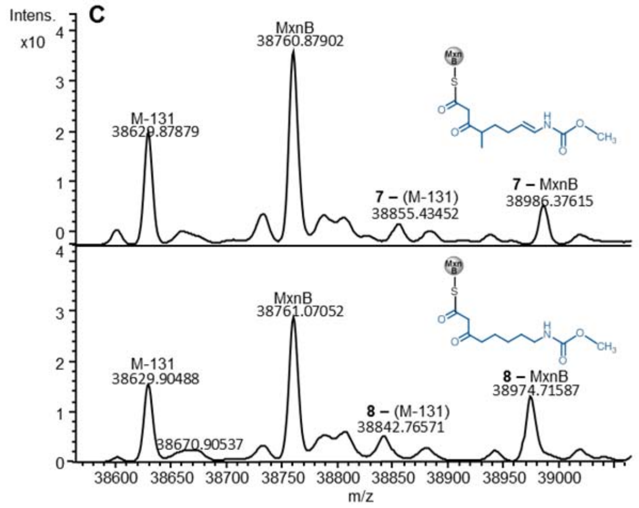

D

Protein	Substrate	Product	
MxnB	**7**	**7**-S-MxnB	11.9 %
	**8**	**8**-S-MxnB	8.9 %

Figure S2. ESI-MS analysis of myxopyronin A produced *in vitro* using substrates **6** and **7** and LC-MS analysis for the *in vitro* formation of MxnB with **7** or **8**. (A) MS spectrum in positive mode of myxopyronin A (**1a**). (B) Fragment spectrum of the parent ion $[M+H]^+$ *m/z* = 418.2224 corresponds to **1a** (calc. for $C_{23}H_{31}NO_6$, 418.2224). The related MS^2 fragments are marked in bold. (C) LC-MS of the reaction mixtures containing MxnB and **7** forming **7**-*S*-MxnB (38,986 Da, top) and MxnB and **8** forming **8**-*S*-MxnB (38,974 Da, bottom). (D) MS analysis of the loading of **7** or **8** (0.025 mM) onto MxnB (7.5 µM). Percentages were calculated using the deconvoluted mass peak heights and they are relative to the total amount of protein species present.

Table S3. NMR Spectroscopic Data for Myxopyronin Derivative (**9**)

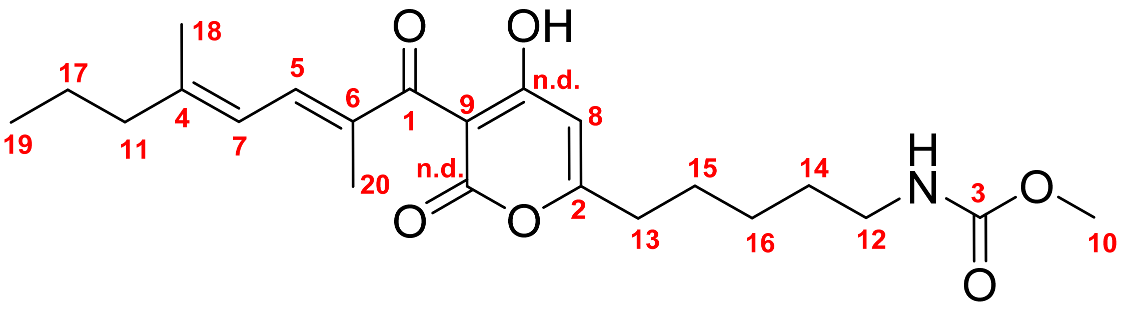

Chemical shifts measured in CD_3OD:

No	δ_C[a]	δ_H(J in Hz)[b]	COSY[c]	HMBC[d]
**1**	199.4	-	-	-
**2**	164.2	-	-	-
**3**	158	-	-	-
**4**	147.9	-	-	-
**5**	136.5	7.32	7	-
**6**	134.5	-	-	-
**7**	121.2	6.26	5	-
**8**	106	5.75	-	2
**9**	101	-	-	-
**10**	50.7	3.61 (s)	-	3
**11**	42.1	2.17 (t, *J*=7.4)	17	4,7,17
**12**	40.1	3.09	14	3,14,15
**13**	32.8	2.42 (t, *J*=7.3)	15	2,15
**14**	29.5	1.51 (m)	12,16	12
**15**	25.9	1.68 (sxt, *J*=7.3)	13,16	13,14
**16**	22.5	1.38 (m)	14,15	-
**17**	20.4	1.53 (m)	11,19	4,11,19
**18**	15.5	1.79 (s)	-	4,7,11
**19**	12.2	0.92 (*J*=7.3)	17	11,17
**20**	9.9	1.92 (s)	-	1,5,6

[a]Recorded at 176 MHz; referenced to residual CD_3OD at δ 49.1. [b]Recorded at 700 MHz; referenced to residual CD_3OD at δ 3.30. [a]Proton showing COSY correlation to indicated proton. [d]Proton showing HMBC key correlations to the indicated carbon. Signals corresponding to quarternary carbon atoms C-1, C-2 and C-9 were assigned from HMBC spectra of an impure fraction (see Experimental Procedures: Isolation of *In Vitro* Product).

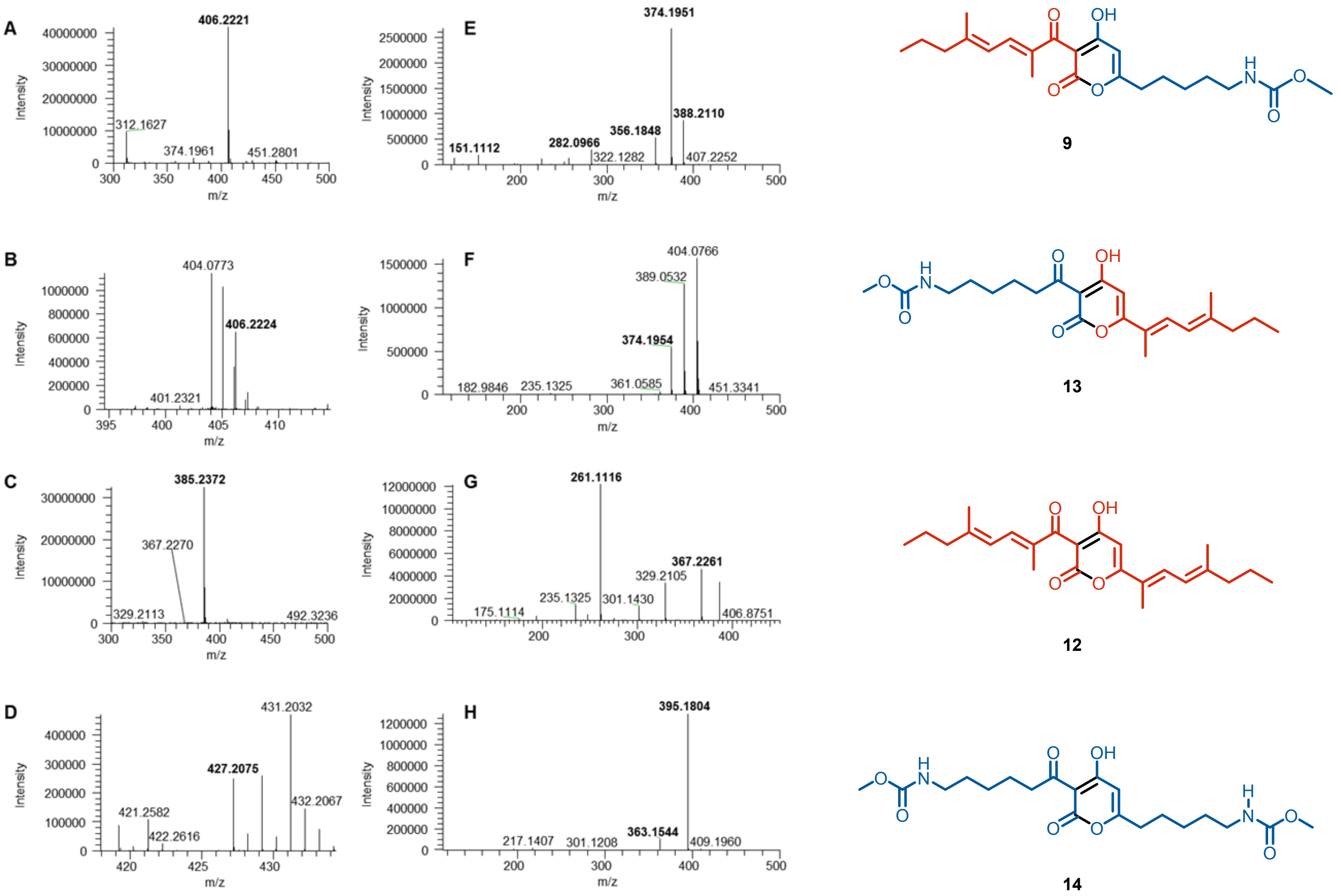

Figure S3. ESI-MS analysis of myxopyronin derivative produced *in vitro* by using substrate **6** and **8**. (A-D) MS spectrum of myxopyronin derivatives (**9**, **13**, **12**, **14**) was recorded in positive mode. (E-H) Fragment spectrum of its corresponding parent ion, respectively. The related MS^2 fragments are marked with bold. The mass at $[M+H]^+$ *m/z* = 406.2221 or 406.2224 corresponds to **9** and the mass at $[M+H]^+$ *m/z* = 406.2224 (calc. for $C_{22}H_{32}NO_6$, 406.2224) to **13**. The mass at $[M+H]^+$ *m/z* = 385.2372 Da corresponds to **12** (calc. for $C_{24}H_{33}O_4$, 385.2373) and the mass at $[M+H]^+$ *m/z* = 427.2075 to **14** (calc. for $C_{20}H_{30}N_2O_8$, 427.2074). (F-H) MS^2 analyses for MxnWE (**9**), MxnEW (**13**), MxnWW (**12**), and MxnEE (**14**) (also see Table S6).

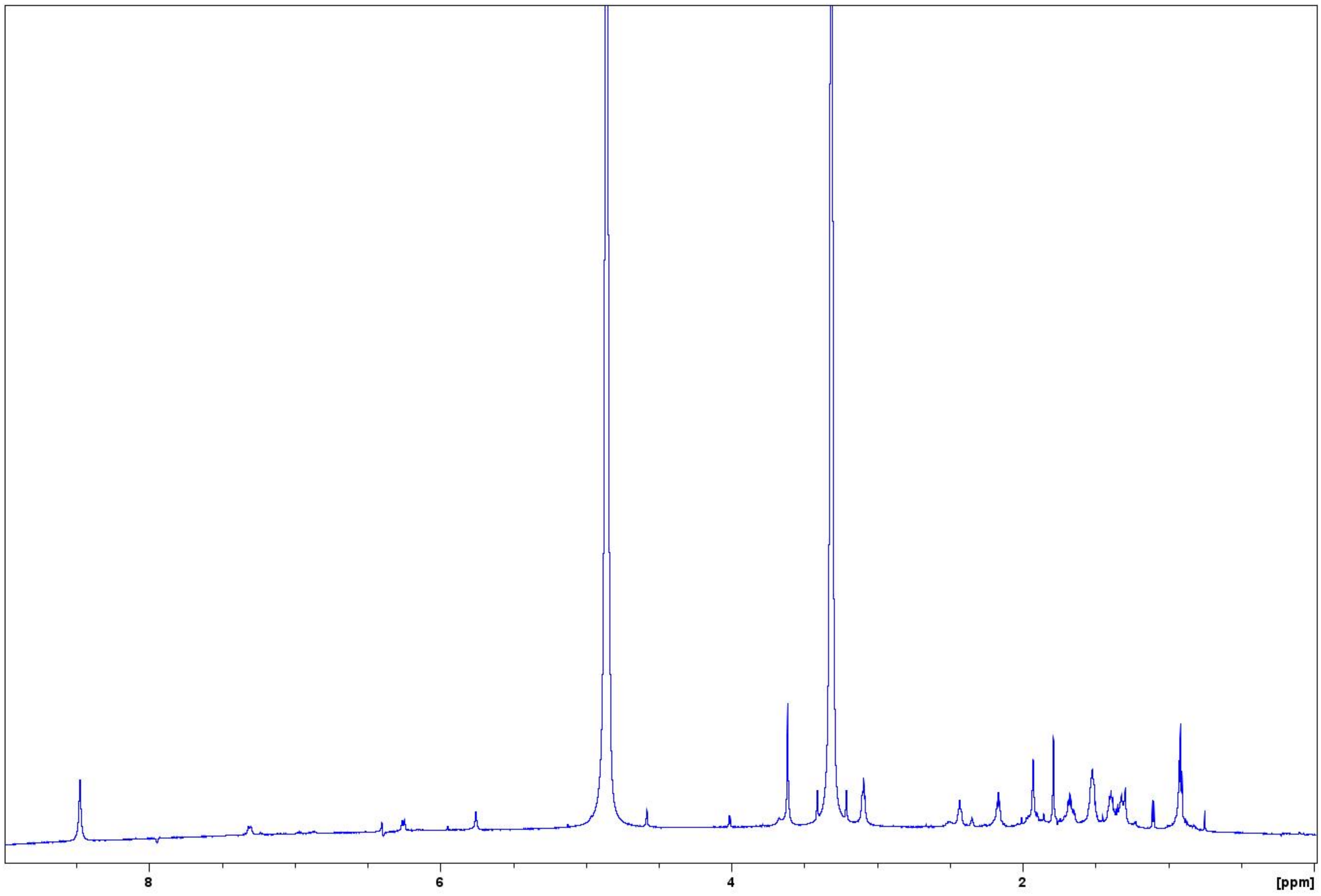

Figure S4a. ^{1}H NMR Spectrum of **9** in CD_3OD at 700 MHz

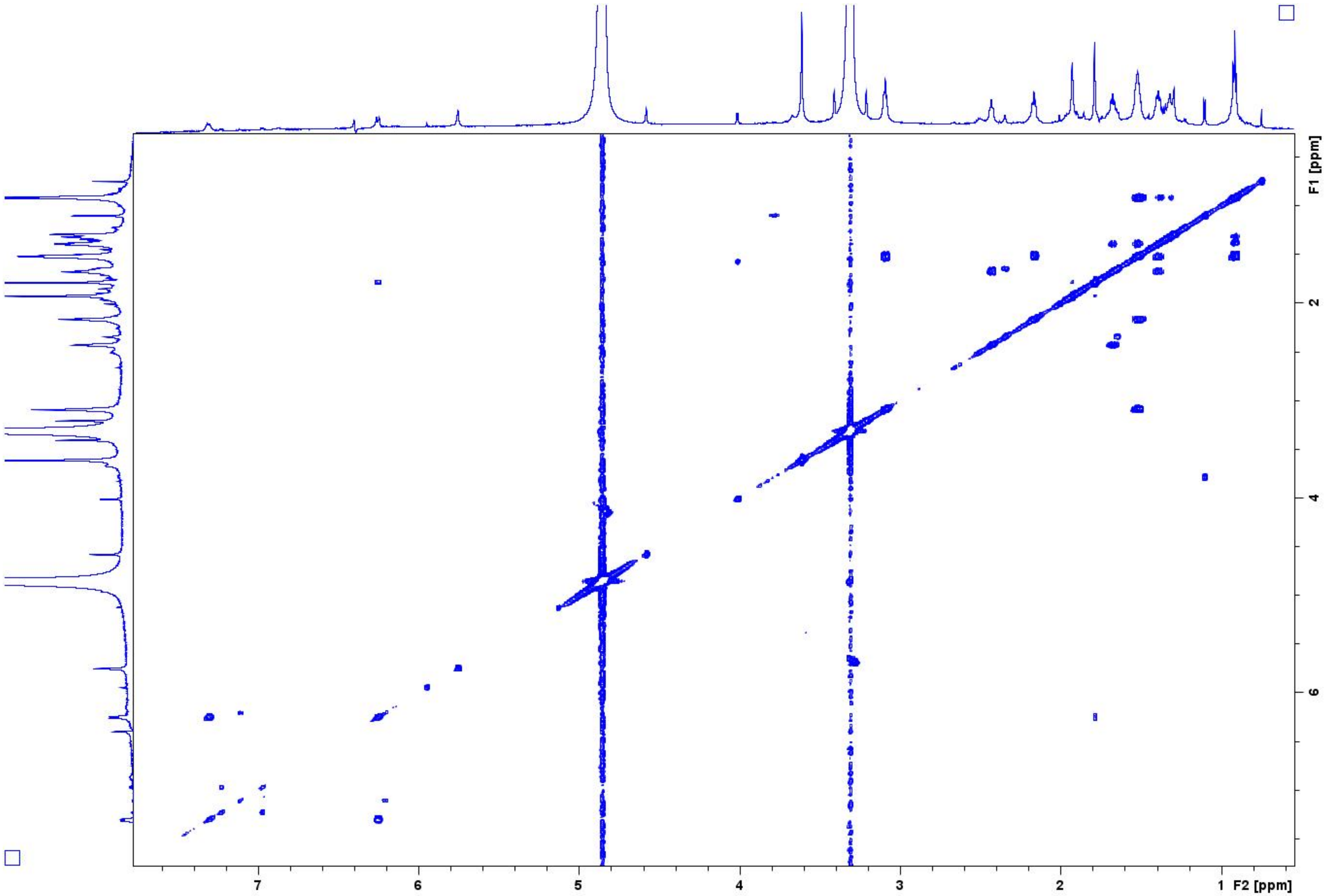

Figure S4b. COSY NMR Spectrum of **9** in CD_3OD at 700 MHz

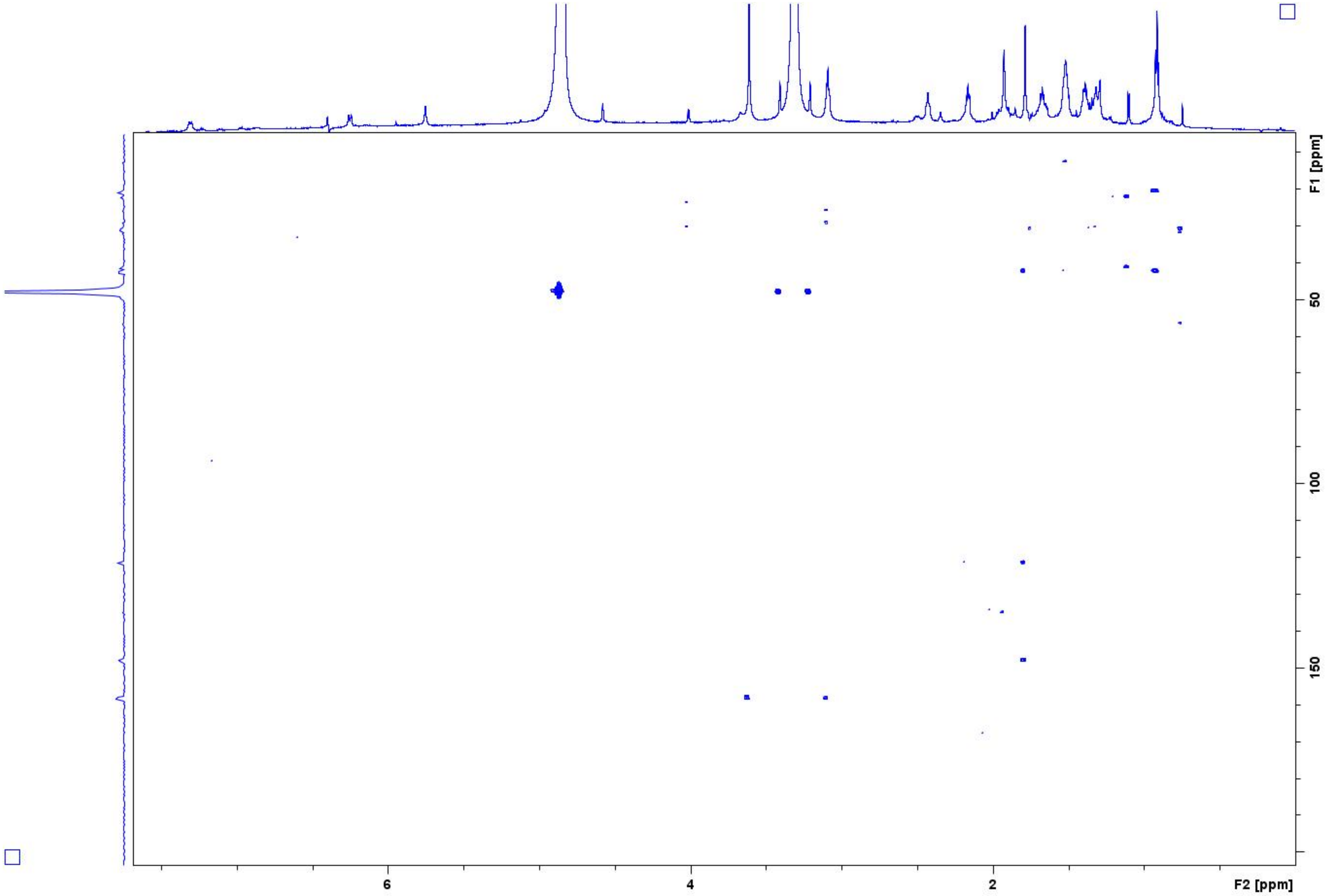

Figure S4c. HMBC NMR Spectrum of **9** in CD_3OD at 700 MHz

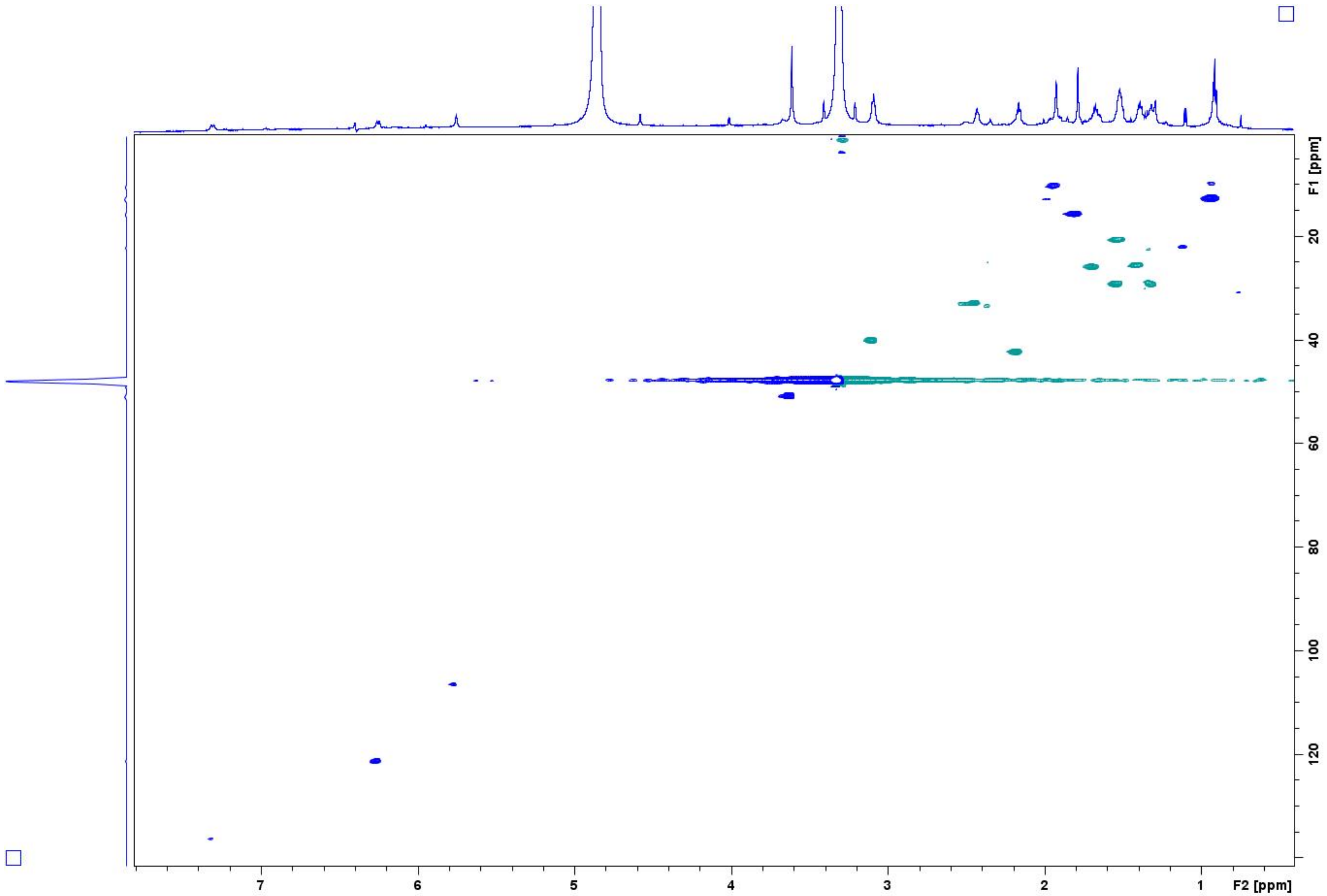

Figure S4d. HSQC NMR Spectrum of **9** in CD_3OD at 700 MHz

Table S4. ESI-MS Analysis of the Recombinant Proteins

Protein	Calculated average mass (Da)		Observed mass (Da)
	Intact protein	Protein – Methionine	
MxnB	38761	38630	38761 and 38629
MxnB# (C121A)	38729	38598	38729 and 35898
MxnB* (H264A)	38695	38564	38695 and 38564
MxnB (T322L)	38773	38642	38772.7 and 38642
MtaA/ACP-W5	14628	14497	14627.7 and 14496.7
MtaA/ACP-E6	14771	14640	14771 and 14640

All proteins were expressed with an N-terminal His_6-tag

Table S5. MS analysis of the loading of **6** or **8** onto MxnB, CP-E and CP-W, which were used in the competition assays.

**Protein**	**Substrate**	**Product**	
MxnB	**6**	**6**-*S*-MxnB	46.45%
	**8**	**8**-*S*-MxnB	27%
CP-E	**6**	**6**-*S*-CP-E	50.05%
	**8**	**8**-*S*-CP-E	16.35%
CP-W	**6**	**6**-*S*-CP-W	37.57%
	**8**	**8**-*S*-CP-W	19.94%

Percentages are calculated using the deconvoluted mass peak heights and they are relative to the total amount of protein species present.

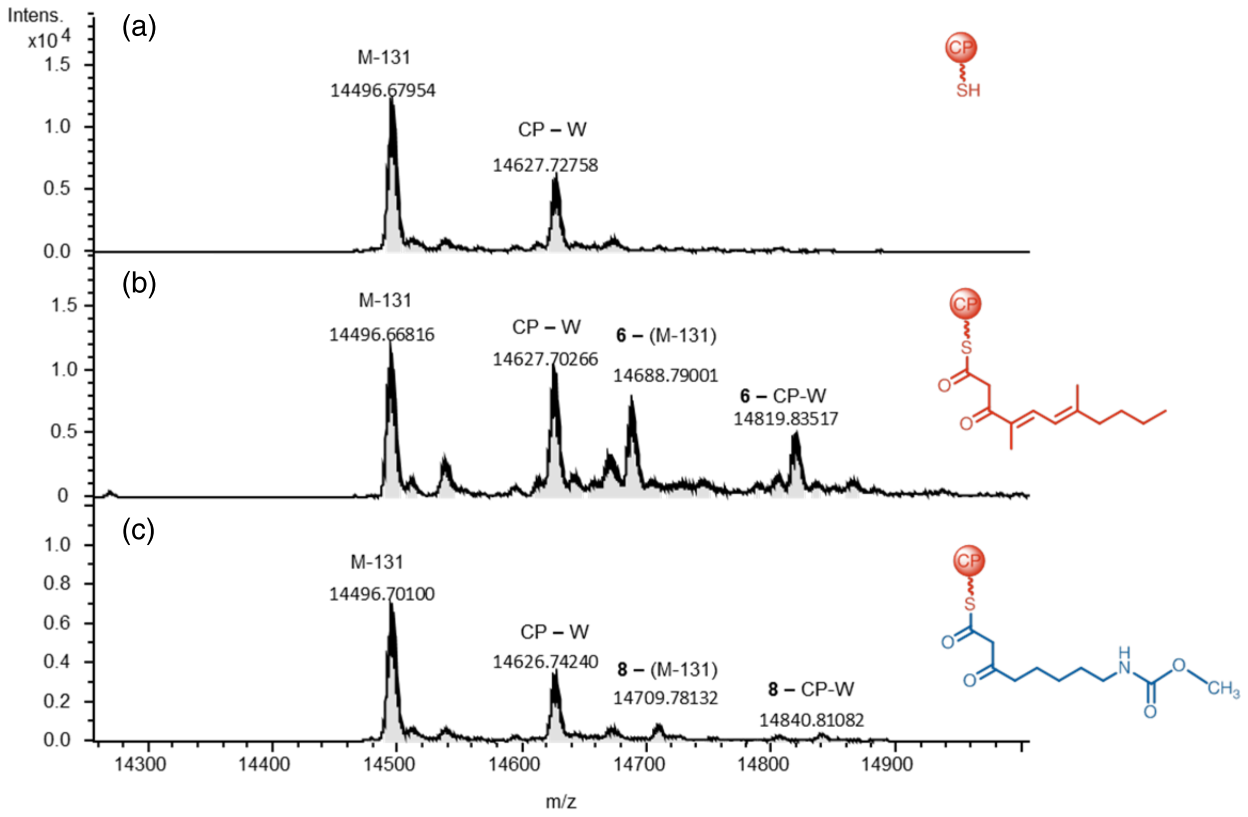

Figure S5. LC-MS analysis of the formation of **6**-*S*-CP-W and **8**-*S*-CP-W. The deconvoluted spectra correspond to (a) holo-CP-W (CP-W), (b) the reaction mixture containing CP-W and **6** forming **6**-*S*-CP-W (14820 Da) and (c) the reaction mixture containing CP-W and **8** forming **8**-*S*-CP-W (14841 Da). The additional peak at M-131 represents the loss of the N-terminal methionine during expression of the recombinant protein in *E. coli*.

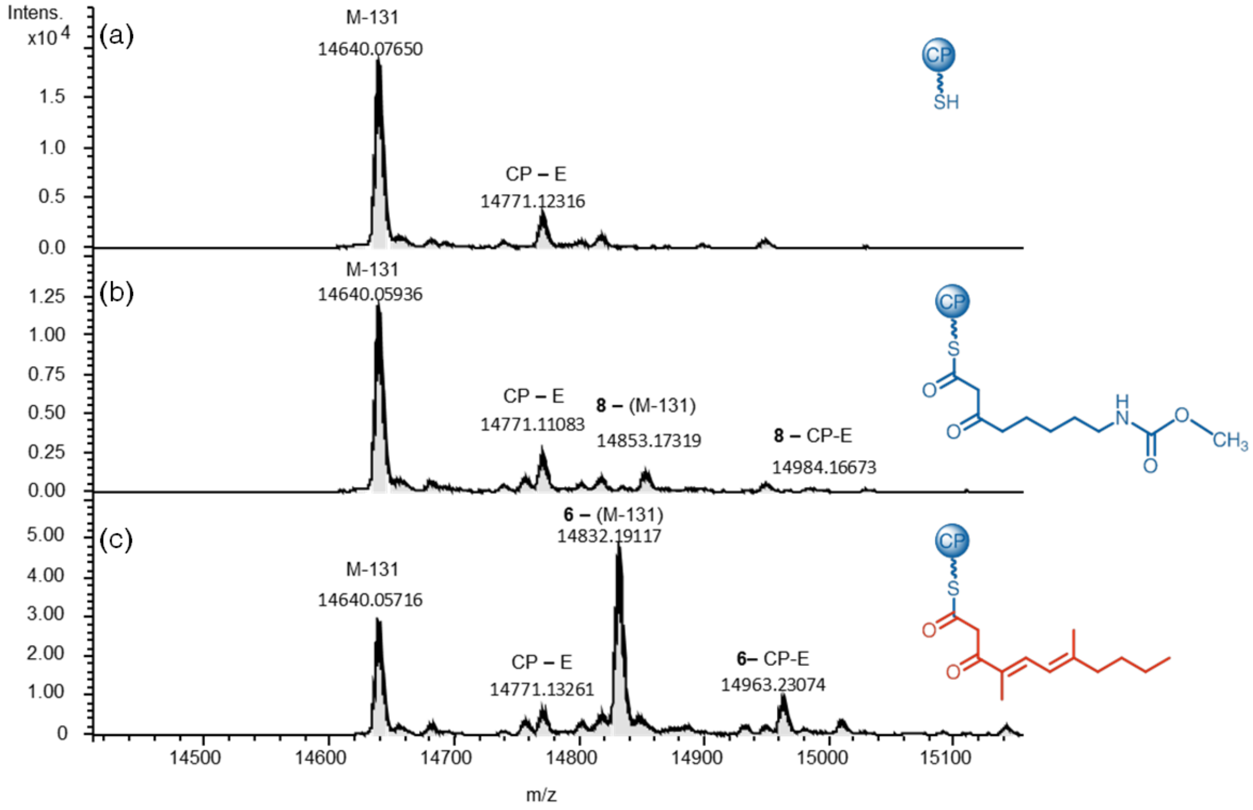

Figure S6. LC-MS analysis for formation of **8**-*S*-CP-E and **6**-*S*-CP-E. The deconvoluted spectra correspond to (a) holo-CP-E (CP-E), (b) the reaction mixture containing CP-E and **8** forming **8**-*S*-CP-E (14984 Da) and (c) the reaction mixture containing CP-E and **6** forming **6**-*S*-CP-E (14963 Da). The additional peak at M-131 represents the loss of the N-terminal methionine during expression of the recombinant protein in *E. coli*.

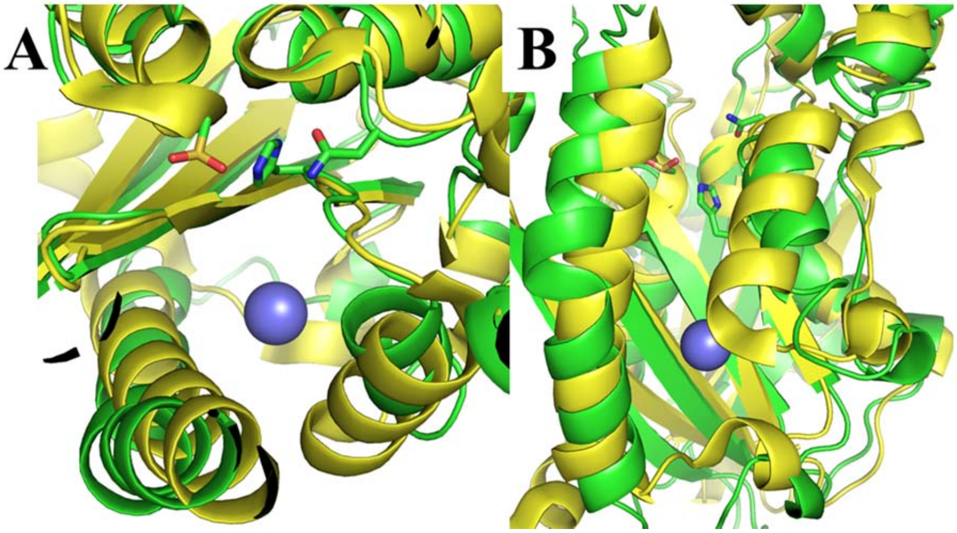

Figure S7. A and B: Superposition of MxnB (green) (PDB ID 4V2P) and OleA (yellow) (PDB ID 3S23). Active site residues of MxnB are shown as sticks. The Xe atom used to identify the second long-chain fatty acid binding channel in OleA is shown as a purple sphere.

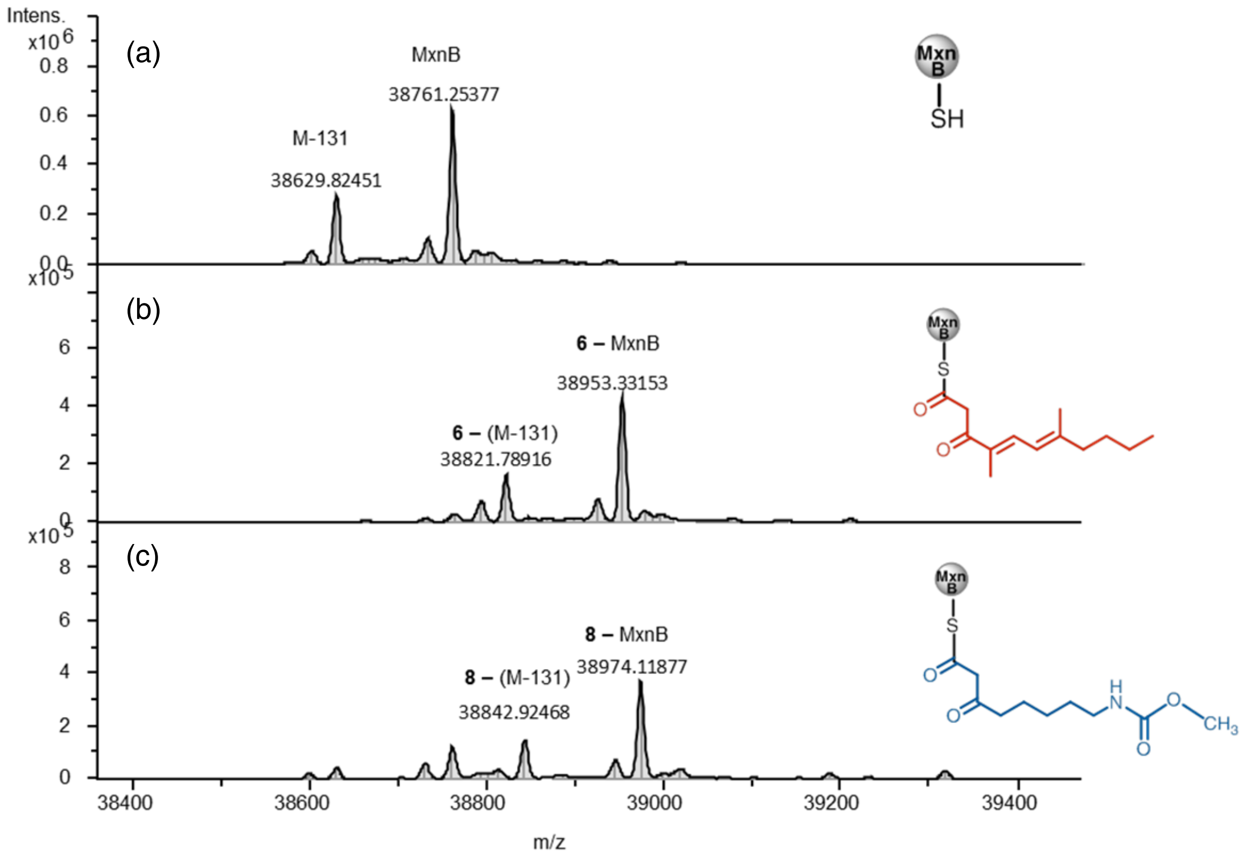

Figure S8. LC-MS analysis for the *in vitro* formation of MxnB. LC-MS analysis for the formation of **6**-S-MxnB and **8**-S-MxnB. The deconvoluted spectra correspond to (a) MxnB, (b) the reaction mixture containing MxnB and **6** forming **6**-*S*-MxnB (38953 Da) and (c) the reaction mixture containing MxnB and **8** forming **8**-*S*-MxnB (38974 Da). The additional peak at M-131 represents the loss of the N-terminal methionine during expression of the recombinant protein in *E. coli*.

Table S6. Characterization of Myxopyronin A and Its Analogues with Representative Fragmentation Patterns Observed

	Chemical Formula	Calculated mass *m/z* [M+H]$^+$	Observed *m/z* [M+H]$^+$	Fragmentation due to losses of				
				H_2O (*m/z* = 18)	CH_3OH (*m/z* = 32)	H_2N-CO_2CH_3 (*m/z* = 75)	H_2N-CO_2CH_3, CO_2 (*m/z* = 119)	Other fragment
MxnA (**1a**)	$C_{23}H_{32}NO_6$	418.2224	418.2224	400.2114	386.1958	343.1899	299.2002	
MxnA (**1a**) from *in vitro* assay	$C_{23}H_{32}NO_6$	418.2224	418.2223	400.2103	386.1953	343.1888	299.1992	
**10**	$C_{23}H_{32}NO_6$	418.2224	418.2224	400.2118	386.1950	343.1895	n.o.	
**11**	$C_{22}H_{30}N_2O_8$	451.2074	451.2076	n.o.	n.o.	n.o.	n.o.	
**12**	$C_{24}H_{33}O_4$	385.2373	385.2375	367.2255	N/A	N/A	N/A	C_9H_{16} (m/z = 124) 261.1155
	Chemical Formula	Calculated mass *m/z* [M+H]$^+$	Observed *m/z* [M+H]$^+$	Fragmentation due to losses of				
				H_2O (*m/z* = 18)	CH_3OH (*m/z* = 32)	C_9H_{16} (m/z = 124)	$C_{12}H_{16}NO_5$ (m/z = 255)	Other fragment 2CH_3OH (*m/z* = 64)
**9**	$C_{22}H_{31}NO_6$	406.2224	406.2219	388.2110	374.1957	282.0966	151.1112	N/A
**13**	$C_{22}H_{31}NO_6$	406.2224	406.2224	n.o.	374.1954	n.o.	n.o.	N/A
**14**	$C_{20}H_{30}N_2O_8$	427.2074	427.2075	n.o.	395.1804	n.o.	n.o.	363.1544
**15**	$C_{21}H_{29}NO_6$	392.2067	392.2065	374.1953	360.1797	282.0962	137.0952	N/A
**16**	$C_{20}H_{27}NO_6$	378.1911	378.1910	360.1794	346.1637	282.0961	123.0796	N/A
**17**	$C_{21}H_{31}NO_6$	394.2224	394.2222	376.2099	362.1949	n.o.	139.1107	N/A
**18**	$C_{21}H_{33}NO_6$	396.2380	396.2377	378.2262	264.2104	n.o.	n.o.	N/A

n.o. = not observed, N/A = not applicable

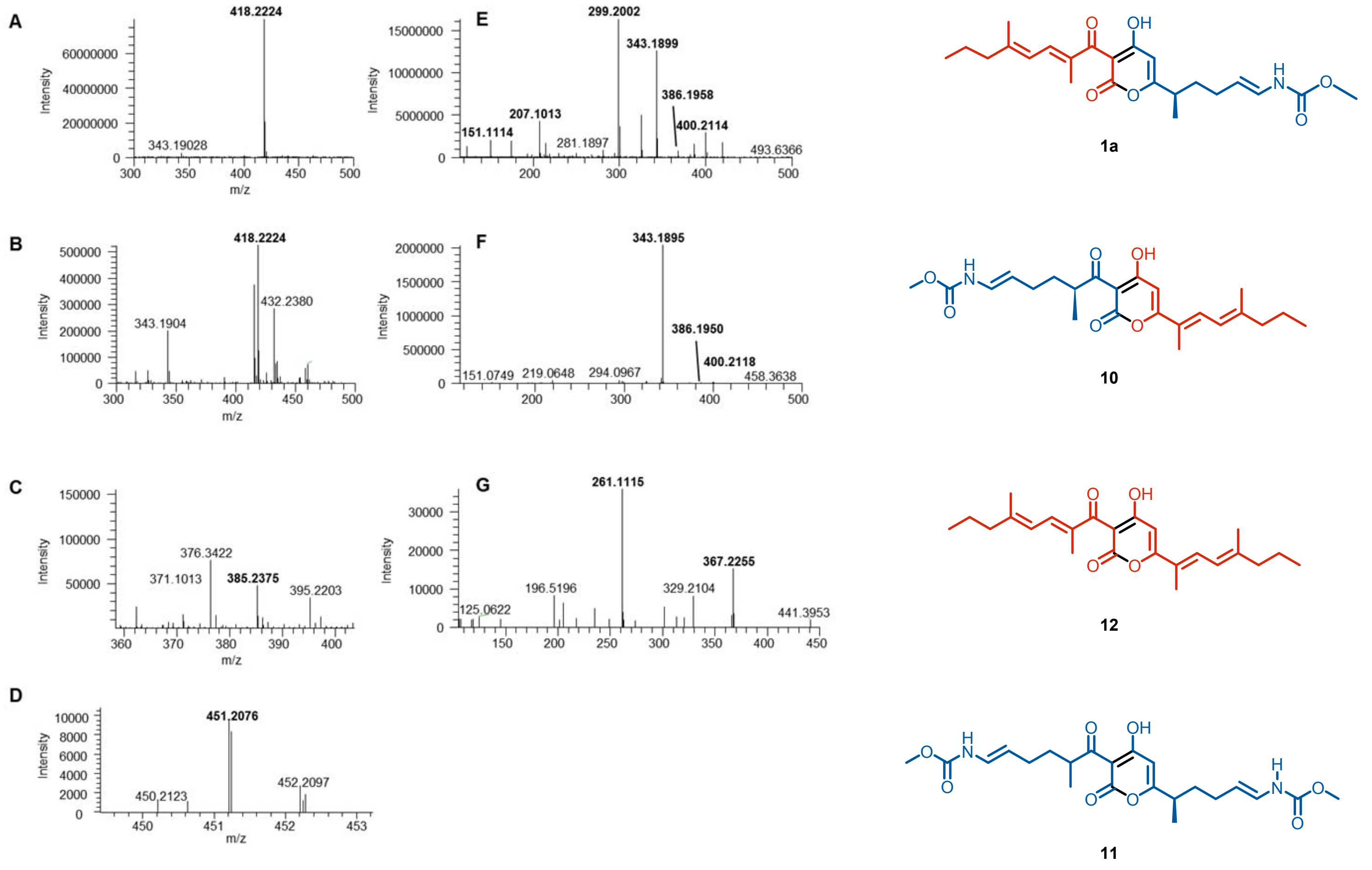

Figure S9. ESI-MS Analysis of myxopyronin and its derivatives produced *in vivo*. (A-D) ESI-MS spectra of myxopyronin A (**1a**) and its derivatives **10**, **12** and **11** in positive mode. (E-G) Fragment spectra of the corresponding parent ion, respectively. The related MS^2 fragments are labelled in bold. The mass at $[M+H]^+$ m/z = 418.2224 correspond to **1a** and **10** (calc. for $C_{23}H_{31}NO_6$, 418.2224). The mass at $[M+H]^+$ m/z = 385.2375 Da correspond to **12** (calc. for $C_{24}H_{33}O_4$, 385.2373) and the mass at $[M+H]^+$ m/z = 451.2076 correspond to **11** (calc. for $C_{22}H_{30}N_2O_8$, 451.2074). (E-G) MS^2 analyses for MxnWE (**1a**), MxnEW (**10**), and MxnWW (**12**) (also see Table S6), while only high-resolution mass spectrometry (HRMS) could be obtained for MxnEE (**11**).

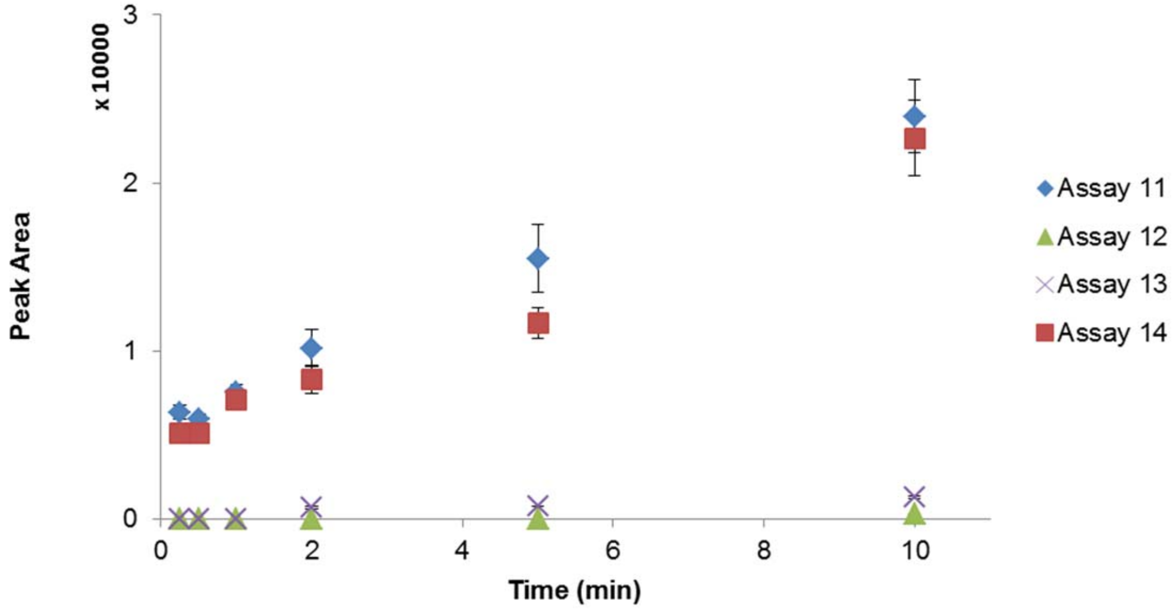

Figure S10. LC-MS analysis of Assays 11-14. Time-course of the production of **9** from 15 s to 10 min. The peak area of extracted ion chromatograms (EIC) for **9** was used for quantification. Assay 11-14: Assay 11 (MxnB, **6**-CP-W and **8-**CP-E), Assay 12 (MxnB, **8**-CP-W, and **6-**CP-E), Assay 13 (MxnB, **6**-CP-W and **8**-CP-W), and Assay 14 (MxnB, **6**-CP-E and **8**-CP-E).

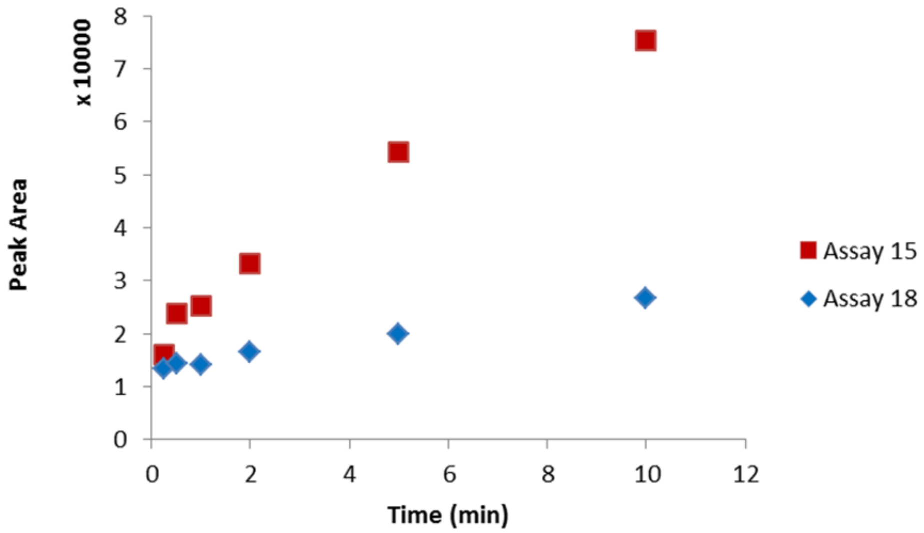

Figure S11. LC-MS analysis of Assays 15-18. The peak area of the EIC for **9** at $[M+H]^+$= 406.22 was used for Assay 15 (**6**-MxnB and **8**-CP-E) and the EIC for **14** at $[M+H]^+$= 427.20 was used for Assay 18 (**8**-MxnB with **8**-CP-E). Assay 16 (**6**-MxnB incubated with **6**-CP-W) and Assay 17 (**8**-MxnB is incubated with **6**-CP-W) are not shown due to the very low amount observed. Assays were performed as a time course between 15 s to 10 min.

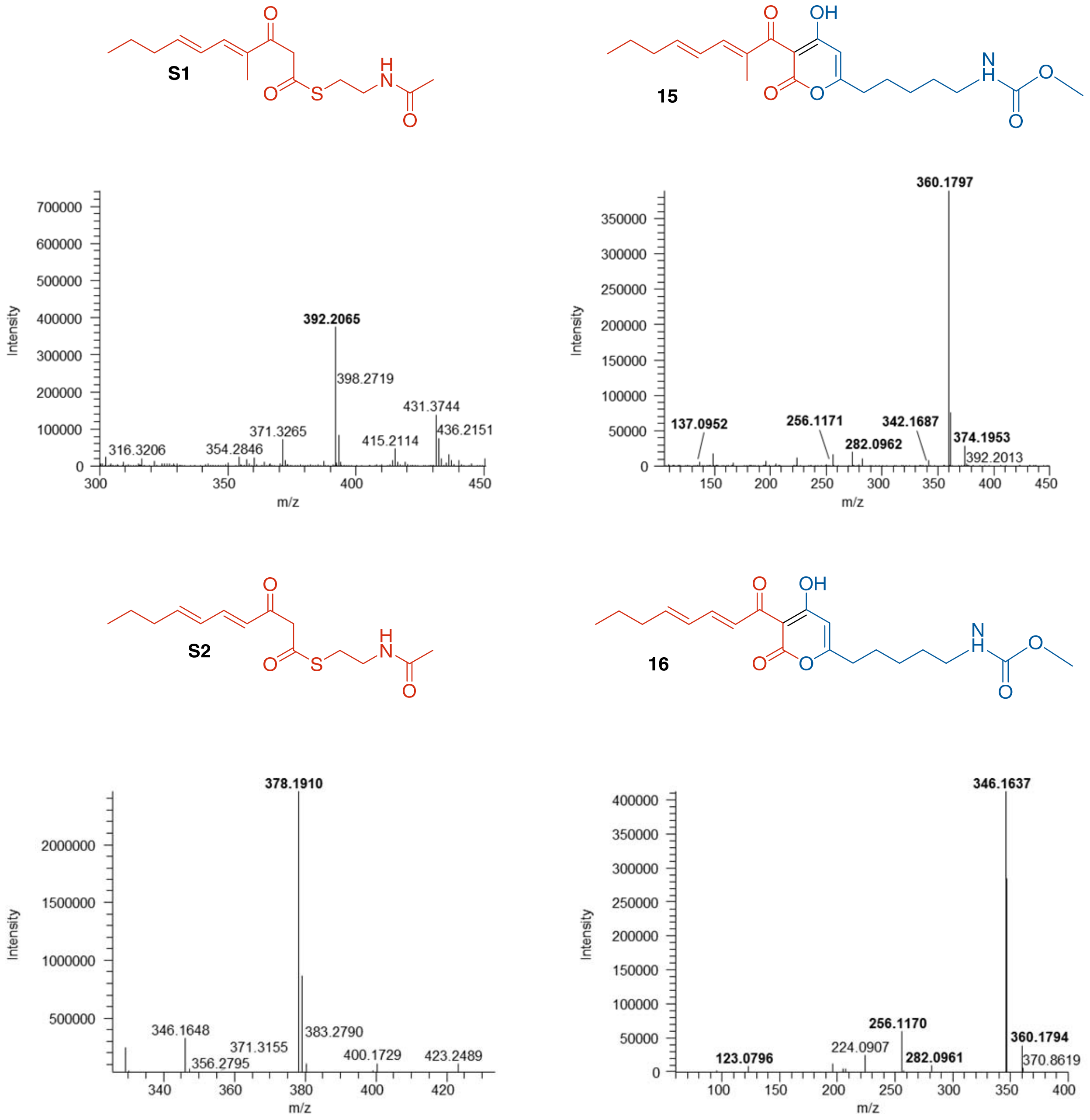

Figure S12. ESI-MS analysis of other myxopyronin derivatives produced *in vitro* in positive mode. The in vitro assays were performed using MxnB, CP-W, CP-E and western SNAC (S1-S4) independently with **8**, resulting in **15**-**18**, respectively. Left panel: MS spectra; right panel: Fragment spectra of the parent ion of **15** $[M+H]^+$ *m/z* = 392.2065, **16** $[M+H]^+$ *m/z* = 378.1910, **17** $[M+H]^+$ *m/z* = 394.2377, **18** $[M+H]^+$ *m/z* = 396.2377 (see also Table S6)

S3

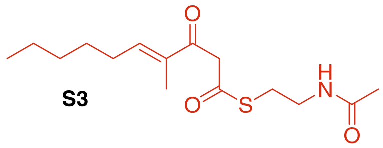

17

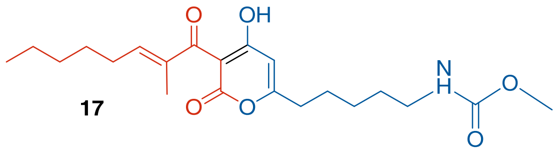

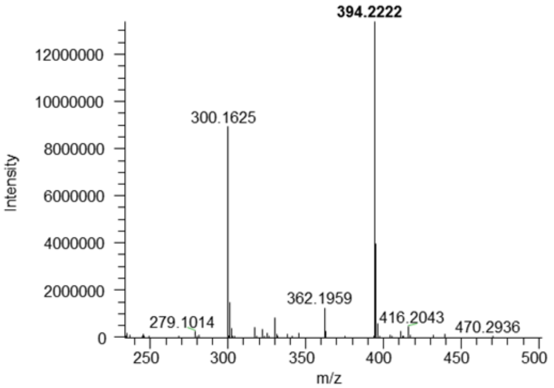

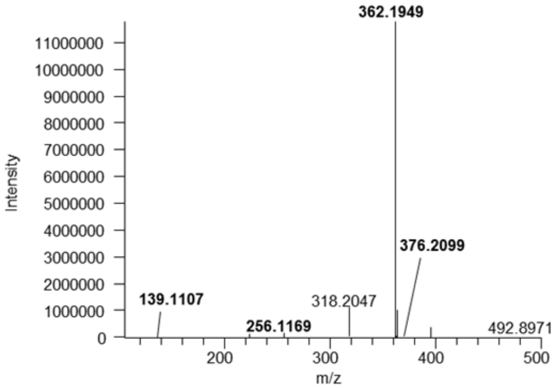

S4

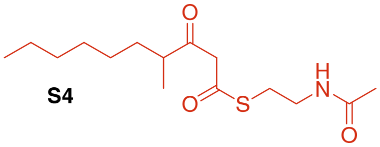

18

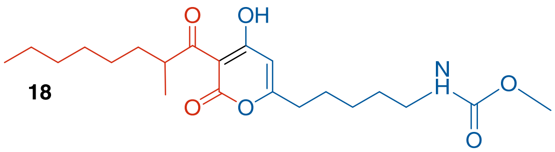

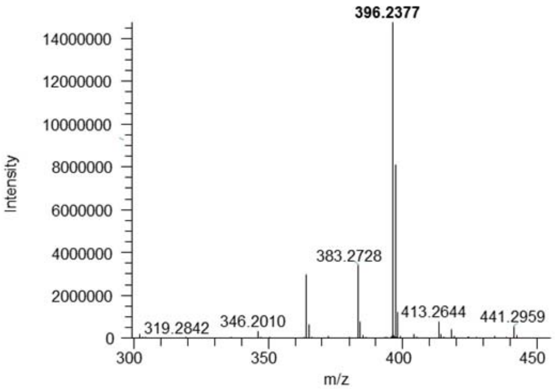

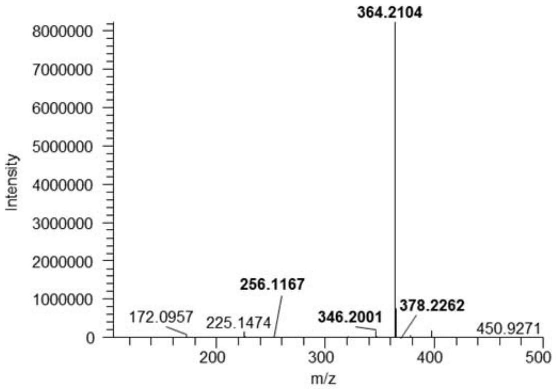

Figure S12 continued. ESI-MS analysis of other myxopyronin derivatives produced *in vitro* in positive mode. The in vitro assays were performed using MxnB, CP-W, CP-E, western SNAC (S1-S4) independently with **8**, resulting in **15**-**18**, respectively. Left panel: MS spectrum; right panel: Fragment spectrum of the parent ion of **15** $[M+H]^+$ *m/z* = 392.2065, **16** $[M+H]^+$ *m/z* = 378.1910, **17** $[M+H]^+$ *m/z* = 394.2377, **18** $[M+H]^+$ *m/z* = 396.2377 (see also Table S6)

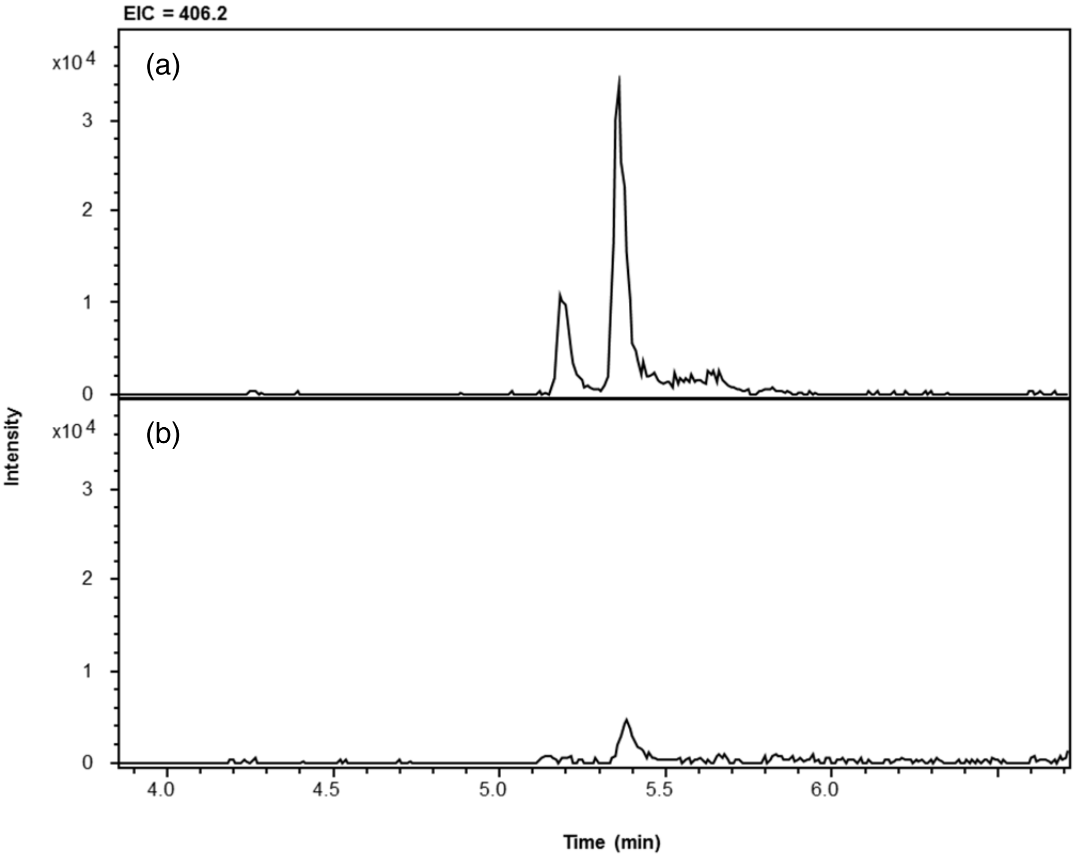

Figure S13. LC-MS analysis of the *in vitro* assay using MxnB mutant T322L. LC-MS analysis in positive mode of *in vitro* assays showing extracted ion chromatograms (EIC) $[M+H]^+$ = 406.2 : a) MxnB, **6**-CP-W and **8**-CP-E and b) MxnB mutant T322L, **6**-CP-W and **8**-CP-E.

Table S7. MS analysis of the loading of **6** or **8** onto MxnB and MxnB mutant T322L

Protein	Substrate	Product	
MxnB	**6**	**6**-*S*-MxnB	87.9%
	**8**	**8**-*S*-MxnB	77.2%
MxnB mutant T322L	**6**	**6**-*S*-MxnB T322L	78.5%
	**8**	**8**-*S*-MxnB T322L	61.5%

Percentages are calculated using the deconvoluted mass peak heights and they are relative to the total amount of protein species present.

Table S8. Data Collection and Refinement Statistics of MxnB Crystal

	MxnB
**Data collection**	
Space group	P1
Cell dimensions	
a, *b*, *c* (Å)	50.19, 58.79, 62.82
α, β, γ (°)	104.75, 105.48, 93.9
Resolution (Å)	1.67 (1.71–1.67)
R_{sym} or R_{merge}	7.7 (41.3)
$I / \sigma I$	9.1 (2.0)
Completeness (%)	92.6 (92.1)
Redundancy	2.2 (2.3)
**Refinement**	
Resolution (Å)	58.05-1.67
No. reflections	71,183
R_{work} / R_{free}	0.146 / 0.177
No. atoms	5,884
Protein	5,130
Ligand/ion	70
Water	684
B-factors	22.20
Protein	20.40
Ligand/ion	54.60
Water	32.60
R.m.s. deviations	
Bond lengths (Å)	0.008
Bond angles (°)	1.24

One crystal was used per dataset. Values in parentheses are for the highest resolution shell. $R_{merge} = \sum_{hkl} \sum_i |I(hkl) - \langle I(hkl) \rangle| / \sum_{hkl} \sum_i I(hkl)$, where $I(hkl)$ is the intensity of an individual measurement and $\langle I(hkl) \rangle$ is the average intensity from multiple observations. $R_{work} = \sum_{hkl} \big| |F_{obs}| - k|F_{calcd}| \big| / \sum_{hkl} |F_{obs}|$ (R_{free} calculated in the same manner by using 5% of the reflection data chosen randomly and omitted from the start of refinement.).

Table S9. ^{18}O incorporation to myxopyronin derivative **9**

Quenching method	*m/z*	Enzyme reaction In $H_2{}^{16}O$	Enzyme reaction In $H_2{}^{18}O$	Incubation of non-labeled **9** product in $H_2{}^{18}O$	Incubation of ^{18}O-labeled **9** product in $H_2{}^{16}O$
Iodoacetamide (Final 1 mM)	406 (0 ^{18}O)	95.2%	92.4%	96.2%	93.8%
	408 (1 ^{18}O)	4.8%	7.6%	3.8%	6.2%
	410 (2 ^{18}O)	0	0	0	0

Note: The percentage of *m/z* of 408 corresponds to the natural abundance of C, H, N, O atoms.

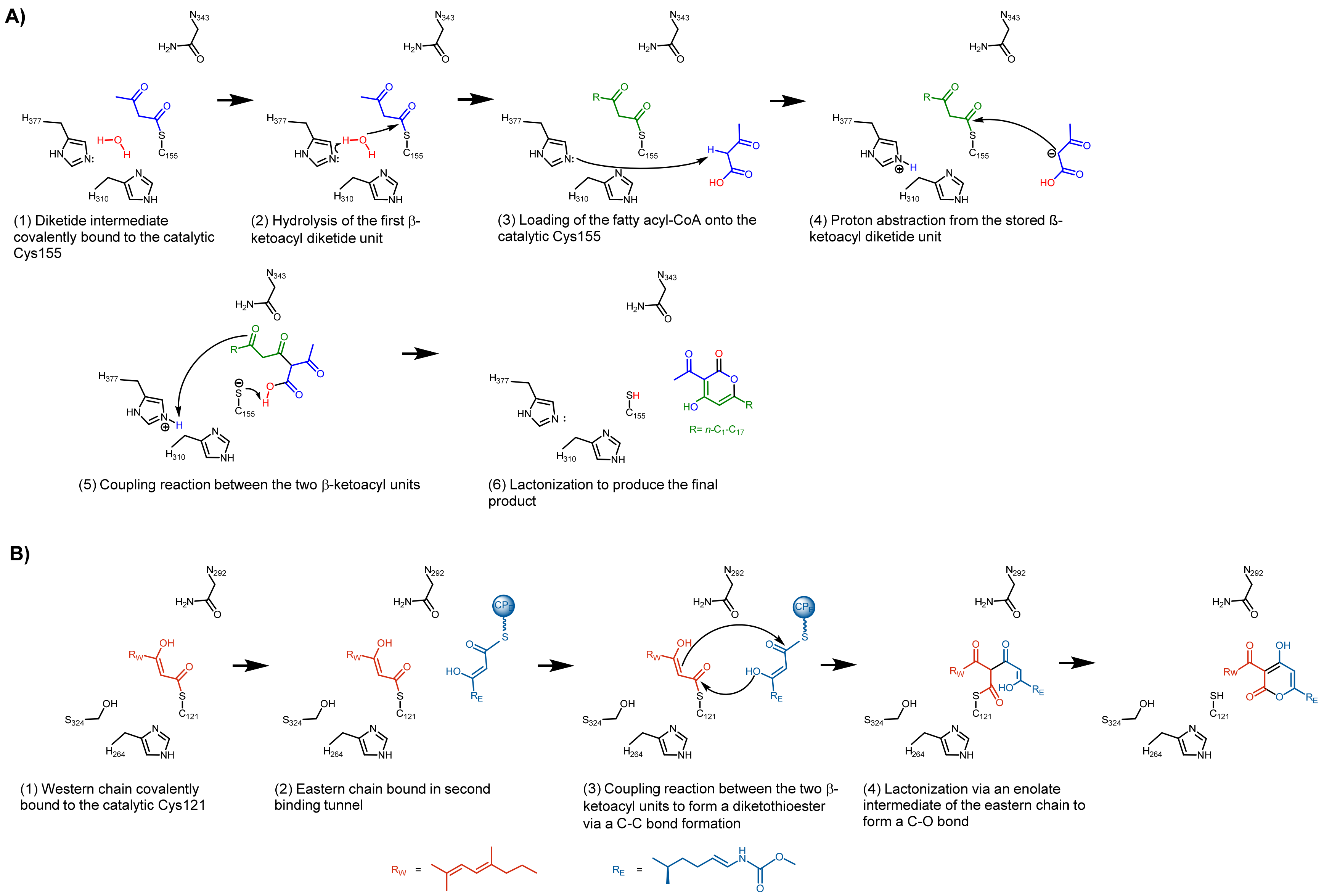

Figure S14. Comparison between proposed mechanisms of A) CsyB and B) MxnB. While CsyB proceeds via a hydrolysis mechanism, no indication for such a mechanism was found for MxnB.

Supplementary Material and Methods for Synthesis Procedures

Synthesis Schemes

Scheme 1: General Synthesis of unsaturated β-keto-*N*-acetylcysteamine (SNAC)-esters

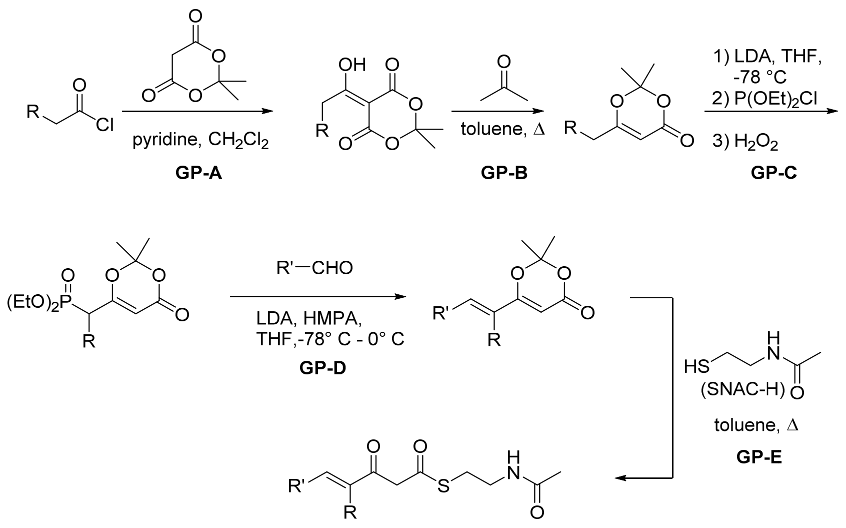

Scheme 2: General synthesis of β-keto-*N*-acetylcysteamine (SNAC)-esters

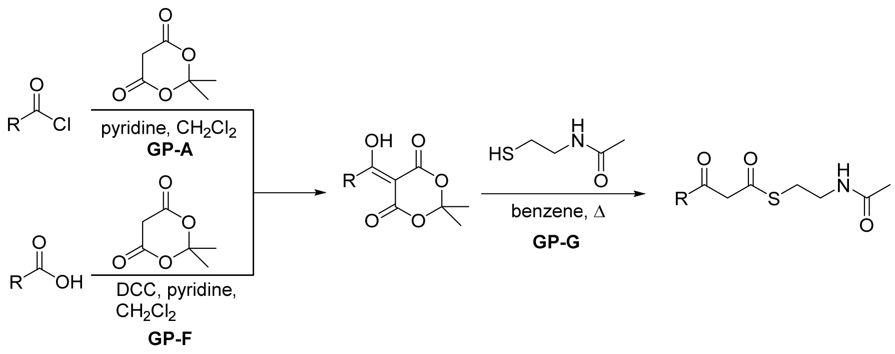

General Procedures

General Procedure for synthesis of acylmeldrums acids from acyl chlorides: General procedure A

To a solution of meldrums acid (10.8 g, 75.0 mmol) and pyridine (12.0 g, 0.15 mol) in anhydrous CH_2Cl_2 (50 mL) at 0 °C was added dropwise a solution of the appropriate acyl chloride (80.0 mmol) in anhydrous CH_2Cl_2 (20 mL). The resulting orange mixture was stirred for 1 h at 0 °C and then for 1 h at room temperature. The reaction mixture was diluted with CH_2Cl_2 (50 mL) and washed with aqueous 2M HCl (3 x 50 mL), brine (2 x 50 mL), dried ($MgSO_4$) and concentrated under reduced pressure. The crude material was filtered over a short pad of SiO_2 eluting with *n*-hexane/ EtOAc 2:1.[10]

General Procedure for synthesis of oxinones from acylmeldrums acids: Genreral procedure B

The appropriate acylmeldrums acid (20.0 mmol) was dissolved in toluene (25 mL) and acetone (2.0 mL). The solution was heated under reflux for 2 h. The solvents were removed under reduced pressure and the crude material was purified by flash chromatography (SiO_2, *n*-hexane/EtOAc) or vacuum distillation.[9]

General Procedure for synthesis of dioxinon phosphonates: General procedure C

To a solution of diisopropylamine (7.18 mL, 51.1 mmol) in THF (30 mL) was added dropwise a solution of *n*-BuLi (2.5M in *n*-hexane, 20.5 mL, 51.1 mmol) at 0 °C. After stirring for 30 minutes, the reaction mixture was cooled to –78 °C and the appropriate 2,2-dimethyl-4*H*-1,3-dioxin-4-one (36.5 mmol) was added over 5 min. After 40 min chlorodiethylphosphite (7.62 mL, 53.0 mmol) was added and the cooling bath was removed. When the mixture reached room temperature, it was diluted with Et_2O (200 mL), washed with half-saturated brine (100 mL), dried ($MgSO_4$) and concentrated *in vacuo*. The resulting yellow oil was dissolved in CH_2Cl_2 (60 mL) and aqueous H_2O_2 (30%, 13 mL) was carefully added at 0 °C. The solution was stirred for 1 h and diluted with EtOAc (200 mL). After separation, the organic layer was washed with brine (2 x 100 mL), dried ($MgSO_4$) and concentrated *in vacuo*. The crude material was purified by flash chromatography (SiO_2, EtOAc). [10]

General Procedure for the Horner-Wadsworth-Emmons olefination for the preparation of unsaturated dioxinones: General procedure D

To a solution of diisopropylamine (1.04 mL, 7.13 mmol) in THF (20 mL) was added a solution of *n*-BuLi (2.5M in *n*-hexane, 2.85 mL, 7.13 mmol) at 0 °C. After stirring for 30 min, the reaction

mixture was cooled to –78 ºC. A solution of the appropriate phosphonate (6.84 mmol) in THF (10 mL) was added to the reaction mixture, and the resulting mixture was warmed to 0 ºC and stirred for 15 min. After cooling to –78 ºC, HMPA (2.10 mL, 12.1 mmol) was added and stirring was continued for 30 min. A solution of the appropriate aldehyde (6.84 mmol) in THF (10 mL) was added slowly. The reaction mixture was allowed to warm slowly to 0 ºC and stirred for 1 h. After diluting with EtOAc (80 mL), the mixture was washed with saturated aqueous NH_4Cl (3 x 40 mL) and brine (50 mL). The organic layer was dried ($MgSO_4$) and concentrated. The crude material was purified by flash chromatography (SiO_2, *n*-hexane/EtOAc). [11]

General Procedure for synthesis of β-keto-*N*-acetylcysteamine (SNAC)-esters from dioxinones: General procedure E

A degassed solution of the appropriate dioxinone (0.95 mmol), *N*-acetylcysteamine (SNAC-H, 0.15 g, 1.25 mmol) in toluene (6 mL) was refluxed for 16 h under a nitrogen atmosphere. After cooling to room temperature the solvent was evaporated under reduced pressure and the crude product was purified by flash column chromatography (SiO_2, *n*-hexane/EtOAc = 1:1 → EtOAc) to afford the desired SNAC ester. [12]

General Procedure for synthesis of acylmeldrums acids from carboxylic acids via DCC coupling: Genreral procedure F

A solution of the appropriate acid (10.6 mmol) and DCC (2.19 g, 10.6 mmol) in anhydrous CH_2Cl_2 (20 mL) was stirred for 30 min at 0°C. A white solid precipitated. DMAP (1.94 g, 15.9 mmol) and meldrums acid (1.52 g, 10.6 mmol) were subsequently added. The cooling bath was removed and the reaction mixture was stirred for 2 h at room temperature. The insoluble urea was filtered off and the filter-cake was washed with ice-cold CH_2Cl_2 (10 mL). The combined filtrates were washed with 1M HCl (2 x 50 mL), H_2O (2 x 50 mL) and brine (75 mL), dried ($MgSO_4$) and concentrated. To remove last residues of urea, the crude material was filtered over cotton. [13]

General Procedure for synthesis of β-keto-*N*-acetylcysteamine (SNAC)-esters from acylmeldrums acids: General procedure G

A degassed solution of the appropriate acylmeldrums acid (0.80 mmol), *N*-acetylcysteamine (SNAC-H, 0.12 g, 1.00 mmol) in toluene (6 mL) was refluxed for 16 h under a nitrogen atmosphere. After cooling to room temperature the solvent was evaporated under reduced pressure and the crude product was purified by flash column chromatography (SiO_2, *n*-hexane/EtOAc = 1:1 → EtOAc) to afford the desired β-keto-SNAC ester.[12]

1 (JHS406)

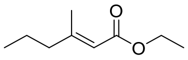

ethyl (E)-3-methylhex-2-enoate

The title compound was prepared according to the following procedure:

To a solution of NaH (1.46 g, 60.9 mmol) in THF (250 mL) was added dropwise a solution of triethyl phosphonoacetate (13.0 g, 58.1 mmol) in THF (50 mL) at 0 °C. The resulting mixture was allowed to warm to room temperature and stirred for 30 minutes. A solution of 2-pentanone (5.00 g, 58.1 mmol) in THF (50 mL) was slowly added and the reaction was heated to 80 °C overnight. The reaction was quenched by addition of a saturated NH_4Cl solution (200 mL). The resulting mixture was extracted with Et_2O (3 x 100 mL). The combined organic layers were washed with H_2O (100 mL) and brine (100 mL) and dried over $MgSO_4$. The solvents were evaporated *in vacuo* to afford a yellow liquid. The crude material was purified by flash column chromatography (SiO_2, *n*-hexane/EtOAc = 20:1) to afford the title compound as a colorless liquid.

Yield: 88%, colorless liquid. *E*/*Z* = 3.2:1, *E*-*i*somer: ^{1}H NMR (300 MHz, $CDCl_3$): δ = 5.66 (q, *J* = 1.20 Hz, 1H), 4.14 (q, *J* = 7.1 Hz, 2H), 2.15 (d, *J* = 1.3 Hz, 3H), 2.08 - 2.14 (m, 2H), 1.51 (sxt, *J* = 7.4, 2H), 1.28 (t, *J* = 7.1 Hz, 3H), 0.91 (t, *J* = 7.4 Hz, 3H) ppm. ^{13}C NMR (75 MHz, $CDCl_3$): δ = 166.9, 159.9, 115.6, 59.4, 42.9, 20.5, 18.6, 14.3, 13.6 ppm.

2 (JHS411)

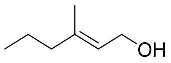

(*E*)-3-methylhex-2-en-1-ol

The title compound was prepared from **1** according to the following procedure:

A suspension of $LiAlH_4$ in anhydrous Et_2O (60 mL) was cooled to 0 °C. $AlCl_3$ was added and the resulting mixture was stirred for 15 minutes. A solution of IM-43 (5.00 g, 32.0 mmol) in Et_2O (15 mL) was added and stirring was continued for 1.5 h at 0 °C. Addition of an aqueous solution of NaOH (5% m/m, 20 mL) lead to precipitation of aluminium hydroxides. The reaction mixture was

filtered over a short bed of $MgSO_4$ which was rinsed with Et_2O (50 mL). Removal of the solvent *in vacuo* afforded the title compound as a colorless liquid.

Yield: 44%, colorless oil. *E*/*Z* = 4:1, *E*-*i*somer: ^{1}H NMR (300 MHz, $CDCl_3$): δ = 5.41 (tq, *J* = 6.9, 1.3 Hz, 1 H), 4.07−4-20, 2.00 (t, *J* = 7.5 Hz, 2 H), 1.66 (d, *J* = 1.3 Hz, 3 H) 1.51−1.39 (m, 2 H) 0.89 (t, *J* = 7.4 Hz, 3 H) ppm. ^{13}C NMR (75 MHz, $CDCl_3$): δ = 139.9, 123.3, 59.4, 41.6, 20.7, 16.1, 13.7 ppm.

3 (JHS412)

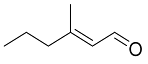

(E)-3-methylhex-2-enal

The title compound was prepared from **2** according to the following procedure:

To a stirred suspension of PCC (4.28 g, 19.9 mmol) and NaOAc (200 mg, 3.98 mmol) in CH_2Cl_2 (100 mL) a solution of the alcohol **IM-40** (1.60 g, 14.0 mmol) in CH_2Cl_2 (100 mL) was added. After 7 h the reaction mixture was diluted with Et_2O (100 mL) and the mixture was filtered over short pad of celite. The solvents were evaporated *in vacuo* and the residue was purified by flash chromatography (SiO_2, *n*-hexane/EtOAc = 20:1 → 10:1).

Yield: 46%, colorless oil. *E*/*Z* = 2:1, *E*-isomer only: ^{1}H NMR (300 MHz, $CDCl_3$): δ = 9.99 (d, *J* = 8.0 Hz, 1 H), 5.87 (dq, *J* = 8.0, 1.2 Hz, 1 H), 2.19 (t, *J* = 7.4 Hz, 2 H), 2.15 (d, *J* = 1.2 Hz, 3 H), 1.54 (q, *J* = 7.4 Hz, 2 H), 0.93 (t, *J* = 7.4 Hz, 3 H) ppm. ^{13}C NMR (75 MHz, $CDCl_3$): δ = 191.3, 164.1, 127.4, 42.6, 20.3, 17.4, 13.6 ppm.

4 (JHS271)

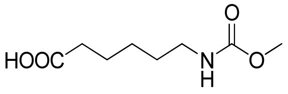

6-(methoxycarbonylamino)hexanoic acid

The title compound was prepared according to the following procedure:

To a stirred solution of γ-amino butyric acid (5.785 g, 56.1 mmol) and KOH (3.209 g, 57.2 mmol) in H_2O (20 mL) were added portionwise, alternately methylchlorofomate (4.82 mL, 62.3 mmol) and a solution of KOH (57.6 mmol) in H_2O (5 mL). After 2 h the reaction mixture was basified by adding a solution of KOH (4.50 g, 80.2 mmol) in H_2O (10 mL). The solution was washed with CH_2Cl_2 (2 x 75 mL). The aqueous layer was acidified with 30% H_2SO_4 causing the precipitation of a white solid. The mixture was extracted with CH_2Cl_2 (4 x 200 mL) and dried ($MgSO_4$). The solvent was evaporated *in vacuo*.

Yield: 88%, colorless oil. ^{1}H NMR (300 MHz, $CDCl_3$): δ = 10.33 (s_{br}, 1 H), 5.88 (s_{br}, 0.30 H, NH), 4.81 (s_{br}, 0.70 H, NH), 3.66 (s, 3 H), 3.20−3.08 (m, 2 H), 2.35 (t, *J* = 7.4 Hz, 2 H), 1.70−1.60 (m, 2 H), 1.56−1.47 (m, 2 H), 1.41−1.31 (m, 2 H) ppm. ^{13}C NMR (75 MHz, $CDCl_3$): δ = 178.9, 157.1, 52.0 (br), 40.8 (br), 33.8, 29.6, 26.0, 24.2 ppm.

5 (JHS273)

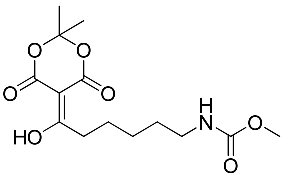

Methyl 6-(2,2-dimethyl-4,6-dioxo-1,3-dioxan-5-ylidene)-6-hydroxyhexylcarbamate

The title compound was prepared from **4** according to the following procedure **F**:

Yield: 99 %, orange oil. ^{1}H NMR (300 MHz, $CDCl_3$): δ = 15.31 (br. s, 1 H), 4.70 (br. s, 1 H), 3.66 (s, 3 H), 3.12−3.25 (m, 2 H), 3.07 (t, *J* = 7.6 Hz, 2 H), 1.30−1.75 (m, 6 H), 1.73 (s, 6 H) ppm. ^{13}C NMR (75 MHz, $CDCl_3$): δ = 197.8, 171.1, 157.1, 104.8, 91.3, 60.3, 52.0, 40.7, 29.6, 26.8, 26.3, 14.2 ppm.

6 (JHS 274) = SNAC Substrate **8**

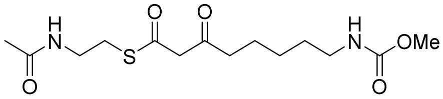

S-2-Acetamidoethyl 8-(methoxycarbonylamino)-3-oxooctanethioate

The title compound was prepared from **5** according to the following procedure **G**:

Yield 56 %, white solid. Keto/enol = 5:1 ^{1}H NMR (300 MHz, $CDCl_3$) keto only: δ = 6.28 (br. s, 1H), 4.94 (br. s, 1H), 3.66 (s, 2H), 3.62 (s, 3H), 3.41 (q, *J* = 6.2 Hz, 2H), 3.13 (q, *J* = 6.4 Hz, 2H), 3.06 (t, *J* = 6.4 Hz, 2H), 2.52 (t, *J* = 7.2 Hz, 2H), 1.97 (s, 3H), 1.63—1.42 (m, 4H), 1.35—1.21 (m, 2H) ppm. ^{13}C NMR (75 MHz, $CDCl_3$) keto only: δ = 201.9, 192.2, 170.5, 157.1, 57.2, 51.9, 43.0, 40.6, 39.0, 29.6, 29.1, 25.8, 23.0, 22.8 ppm.

7 (JHS190D)

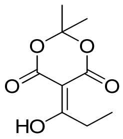

5-(1-hydroxypropylidene)-2,2-dimethyl-1,3-dioxane-4,6-dione

The title compound was prepared from propionyl chloride according to the general procedure **A**:

Yield: 93%, orange crystals. ^{1}H NMR (300 MHz, $CDCl_3$): δ = 15.35 (s_{br}, 1 H), 3.10 (q, *J* = 7.4 Hz, 2 H), 1.72 (s, 6 H), 1.25 (t, *J* = 7.4 Hz, 3 H) ppm.^{13}C NMR ((75 MHz, $CDCl_3$): δ = 198.9, 170.2, 160.2, 104.8, 90.9, 29.4, 26.7, 9.6 ppm.

8 (MAGR35)

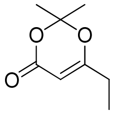

6-Ethyl-2,2-dimethyl-4*H*-1,3-dioxin-4-one

The title compound was prepared from **7** according to the general procedure **B**:

Yield: 80%, yellow oil. ^{1}H NMR (300 MHz, $CDCl_3$): δ = 5.22 (t, *J* = 1.0 Hz, 1 H), 2.24 (dq, *J* = 7.6, 1.0 Hz, 2 H), 1.67 (s, 6 H), 1.12 (t, *J* = 7.6 Hz, 3 H) ppm. ^{13}C NMR (75 MHz, $CDCl_3$): δ = 173.1, 161.5, 106.2, 92.2, 26.7, 24.9, 9.9 ppm.

9 (JHS 195)

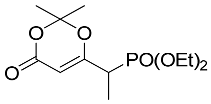

Diethyl 1-(2,2-dimethyl-4-oxo-4H-1,3-dioxin-6-yl)ethylphosphonate

The title compound was prepared from **8** according to the general procedure C:

Yield: 75%. Light yellow oil. ^{1}H NMR (300 MHz, $CDCl_3$): δ = 5.37 (d, J = 3.5 Hz, 1 H), 4.08−4.18 (m, 4 H), 2.77 (dq, J = 23.9, 7.3 Hz, 1 H), 1.69 (s, 6 H), 1.30−1.45 (m, 9 H) ppm.^{13}C NMR (75 MHz, $CDCl_3$): δ = 168.1 (d, J = 8.2 Hz), 160.8 (d, J = 2.2 Hz), 106.8, 94.9 (d, J = 6.7 Hz), 62.1 (dd, J = 6.7, 4.5 Hz), 36.9 (d, J = 137.1 Hz), 24.8 (d, J = 70.8 Hz), 16.4 (d, J = 6.0 Hz), 12.1 (d, J = 6.0 Hz) ppm.

10 (JHS485)

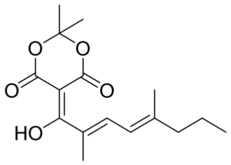

5-((2E,4E)-1-hydroxy-2,5-dimethylocta-2,4-dien-1-ylidene)-2,2-dimethyl-1,3-dioxane-4,6-dione

The title compound was prepared from **9** using **3** as aldehyde according to the general procedure D:

Yield: 60 %, yellow oil. ^{1}H NMR (300 MHz, $CDCl_3$) δ 7.09 (d, J = 11.8 Hz, 1H), 6.14 (dd, J = 1.2, 11.8 Hz, 1H), 5.41 (s, 1H), 2.12 (t, J = 7.5 Hz, 2H), 1.85 (s, 3H), 1.83 (s, 3H), 1.68 (s, 6H), 1.47 (sxt, J = 7.4 Hz, 2H), 0.87 (t, J = 7.4 Hz, 3H) ppm. ^{13}C NMR (75 MHz, $CDCl_3$) δ 166.0, 162.3, 148.5, 130.0, 124.0, 120.6, 105.7, 91.2, 42.7, 24.8, 20.9, 17.0, 13.6, 11.9 ppm.

11 (JHS486) = SNAC Substrate **6**

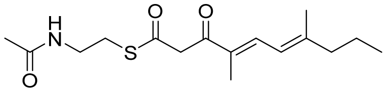

S-(2-acetamidoethyl) (4E,6E)-4,7-dimethyl-3-oxodeca-4,6-dienethioate

The title compound was from **10** prepared according to the general procedure E:

Yield 65 %. Keto/enol = 4:1, 4*E*6*E*/4*Z*6*E* = 2:1. ^{1}H NMR (300 MHz, acetone-d6) 4*E*6*E*-*i*somer keto only: δ = 7.49 (dd, J = 1.2, 11.5 Hz, 1H), 7.29 (br. s, 1H), 6.27−6.37 (m, 1H), 4.12 (s, 2H), 3.32 (q, J = 6.8 Hz, 2H), 3.02 (t, J = 6.8 Hz, 2H), 2.15−2.25 (m, 2H), 1.95 (s, 3H), 1.85 (s, 3H), 1.84 (s, 3H), 1.53 (sxt, J = 7.4 Hz, 2H), 0.91 (t, J = 7.4 Hz, 3H) ppm. ^{13}C NMR (75 MHz,

acetone-d6) δ 194.0, 193.5, 170.2, 151.7, 138.2, 133.9, 122.1, 53.9, 43.5, 39.6, 29.8, 22.9, 21.7, 17.5, 14.1, 11.4 ppm.

S1-S4 Substrates Synthesis Procedure

The substrates were synthesized as previously described.[14]

Scheme 3:

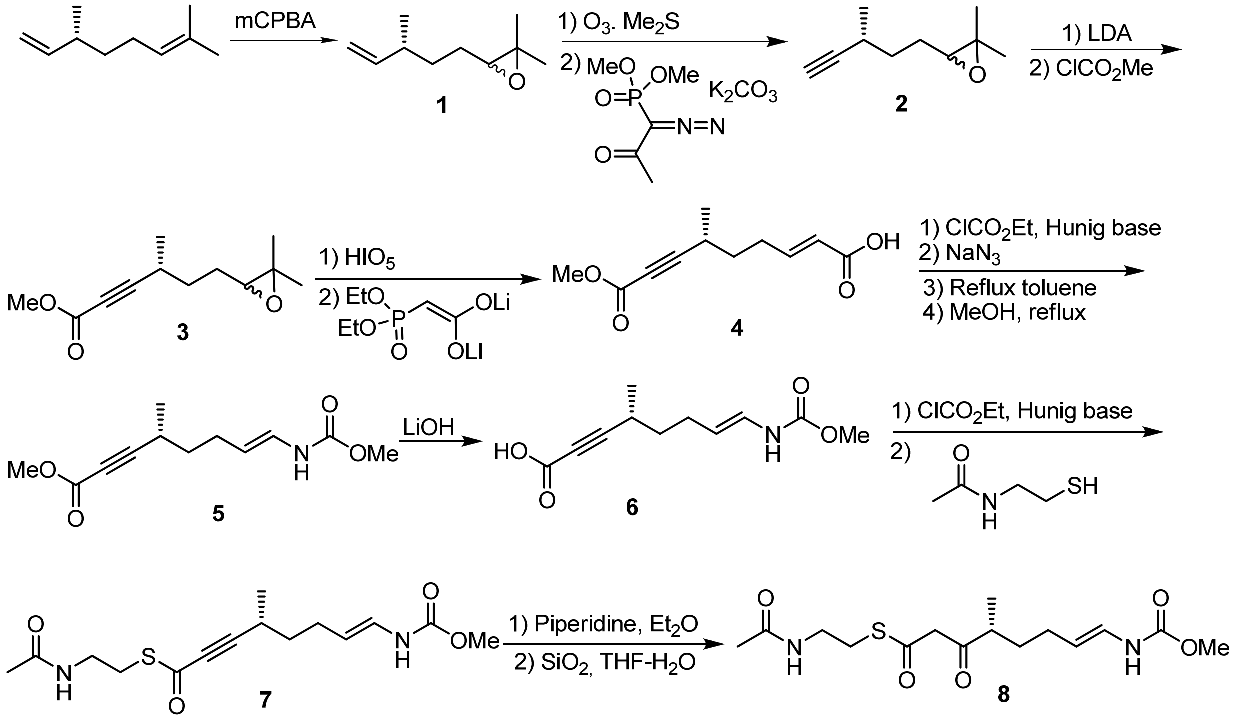

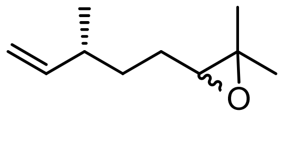

3-Chloroperoxybenzoic acid (75%, 27.8 g, 120 mmol) was added in small portions over a period of 50 min to a solution of ß-(-)-citronellene (13.6 g, 14.5 ml, 100 mmol) and sodium acetate (9.84 g, 120 mmol) in CH_2Cl_2 (250 mL) at -20 °C and stirring was continued at this temperature for 20 min. The suspension was warmed to 0 °C, stirred for 20 min and quenched by the

addition of saturated aqueous $NaHCO_3$ (150 mL). The layers were separated, the aqueous phase extracted with CH_2Cl_2 (3 × 50 mL) and the combined organic fractions were washed with aqueous NaOH (1M, 100 mL), dried over Na_2SO_4 and concentrated in vacuo to final volume of approx. 100 mL. The crude product **1** was used further without isolation and purification.

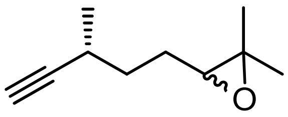

(R)-2,2-dimethyl-3-(3-methylpent-4-ynyl)oxirane. A stream of ozone was passed through an aliquot (30 mL, corresponds to approx. 30 mmol of the epoxide **1**) of the above solution at -78 °C until the blue color persisted at which time the ozonator was turned off and the reaction mixture was purged with oxygen stream for 5 min. Dimethyl sulfide (4.4 mL, 60 mmol) was added in one portion and the reaction was stirred for 1 h at -78 °C and then allowed to warm to rt over 1 h whereupon it was diluted with 30 ml of methanol. Dimethyl-1-diazo-2-oxopropylphosphonate (6.34 g, 33 mmol) and finely powdered K_2CO_3 (8.4 g, 60 mmol) were added sequentially. The mixture was stirred for 4 h, diluted with water (500 mL) and extracted with pentane (3 × 50 mL). The combined organic extracts were washed with water, brine, dried over Na_2SO_4 and concentrated in vacuo (650 mbar, bath temp. 40 °C). The residue was purified by column chromatography (petroleum ether/Et_2O = 10:1) to give alkyne **2** (1.52 g, 35% over two steps) as a mixture of diastereomers. Care should be taken during evaporation of solutions of this volatile compound, complete removal of residual diethyl ether is not essential for the next step.

^{1}H-NMR (500 MHz, $CDCl_3$): δ = 2.72-2.75 (m, 1H), 2.45-2.54 (m, 1H), 2.06 (d, J = 2.4 Hz, 1H), 1.50-1.80 (m, 4H), 1.32 (d, J = 1.5 Hz, 3H), 1.29 (s, 3H), 1.20-1.23 (m, 3H) ppm; ^{13}C-NMR (125 MHz, $CDCl_3$): δ = 88.4, 88.4, 68.7, 68.6, 64.2, 63.9, 58.4, 58.3, 33.7, 33.2, 27.0, 26.3, 25.8, 25.3, 24.8, 21.1, 20.8, 18.7, 18.7 ppm.

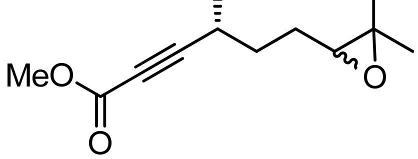

To a solution of diisopropylamine (3.3 mL, 25 mmol) in 100 mL of THF at -78°C was added n-BuLi (10 mL, 2.5 M solution in hexanes, 25 mmol) via syringe. The solution was warmed to 0 °C and stirred for 10 min. The reaction was recooled to-78°C and a solution of alkyne **2** (3.4 g, assumed 25 mmol) in THF (4 mL) was added dropwise over 5 min via cannula. The solution was stirred for 20 min, after which, methyl chloroformate (3.8 mL, 50 mmol) was rapidly added via syringe in one portion. After stirring for 15 min at -78°C, the reaction was quenched by the addition of saturated aqueous NH_4Cl (10 mL) and extracted with Et_2O (3 ×10 mL). The combined organic layers were dried over $MgSO_4$, filtered, and concentrated in vacuo. Purification via flash chromatography (Et_2O/petrol ether = 1:4) afforded (3.15 g, 60%) of **3** as a colorless oil.

^{1}H-NMR (500 MHz, $CDCl_3$): δ = 1.25-1.28 (m, 3H), 1.29 (s, 3H), 1.32 (d, J = 1.5 Hz, 3H), 1.51-1.81 (m, 4H), 2.58-2.76 (m, 2H), 3.76 (m, 3H) ppm; ^{13}C-NMR (125 MHz, $CDCl_3$): δ = 154.2, 92.8, 92.7, 73.4, 63.9, 63.5, 58.4, 58.3, 52.6, 33.0, 32.5, 27.0, 26.2, 26.1, 25.5, 24.8, 20.0, 19.7, 18.7, 18.7 ppm.

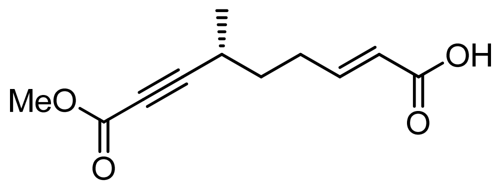

The ester **3** (3 g, 14 mmol) was dissolved in Et_2O/THF (25/25 mL) and cooled to 0 °C. Periodic acid (3.2 g, 16 mmol) was added to this solution over a period of 30 min and stirring was continued for 1 h with slow warming to rt. The solution was diluted with Et_2O (50 mL) and H_2O (100 mL), the organic layer was separated and the water phase was extracted with Et_2O (2 × 25 mL). Combined organic phases were washed with brine (2 × 100 mL), dried over Na_2SO_4 and evaporated. The crude aldehyde was used in the next step without purification.

2-(diethoxyphosphoryl)acetic acid (2.75 g, 14 mmol) in THF (5 mL) was added to a solution of n-butyllithium (2.5 M in Hexan, 11 mL, 28 mmol) in THF (100 mL) at -60 °C. The reaction mixture was stirred for 30 min and aldehyde 14 (41 mg, 120 µmol) in THF (1.5 mL) was added over a period of 5 min. Stirring was continued for 1 h at -60 °C and for 1 h at room temperature. Water (20 mL), EtOAc (20 mL) and HCl (0.1 M, 1.2 mL) were added. The organic phase was separated, dried over MgSO4 and concentrated in vacuo. The obtained crude acid **4** (1.3 g, 45%) was used further as is.

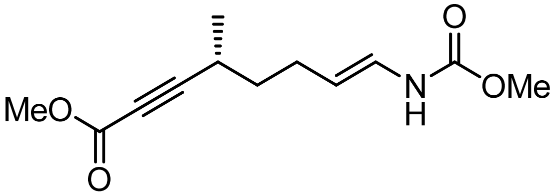

Diisopropylethylamine (2.4 mL, 14 mmol) and ethyl chloroformate (570 µL, 6 mmol) were added successively to a solution of acid **4** (1.05 g, 5 mmol) in acetone (40 mL) at 0 °C. The reaction mixture was stirred for 1 h, NaN_3 (780 mg, 12 mmol) in water (20 mL) was added and stirring was continued for 1 h. The solution was poured on ice water (200 mL) and extracted with ethyl acetate (3 × 25 mL). The combined organic phases were dried over Na_2SO_4 and concentrated in vacuo. The crude product was dissolved in toluene (40 mL) and refluxed for 1 h. Methanol (10 mL) was added, the heating bath temperature lowered to 70 °C and stirring was continued for 16 h. The solution was cooled to room temperature and concentrated in vacuo. Purification by flash column chromatography (petroleum ether/EtOAc = 3:1 then 2:1) gave vinyl carbamate **5** (537 mg, 45%) as a colorless oil.

^{1}H-NMR (500 MHz, CD_3OD): δ = 6.43 (d, J = 14.2 Hz, 1H), 5.93-5.09 (dt, J = 14.2, 7.2 Hz, 1H, 1H), 3.73 (s, 3H), 3.67 (s, 3H), 2.59-2.66 (m, 1H), 2.07-2.20 (m, 2H), 1.50-1.58 (m, 2H), 1.22 (d, J = 7.1 Hz, 3H) ppm; ^{13}C-NMR (125 MHz, CD_3OD): δ = 156.8, 155.7, 126.0, 110.4, 94.0, 74.3, 53.0, 52.7, 37.5, 28.6, 26.2, 20.2 ppm.

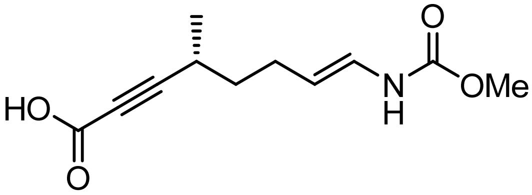

To a solution of **5** (500 mg, 2.1 mmol) in THF (5 mL) and water (1 mL) at room temperature was added LiOH (100 mg, 4 mmol). After stirring for 3 h, reaction mixture was added to a vigorously stirred mixture of 10% sodium dihydrophosphate solution (10 mL) and ethyl acetate (10 mL). The organic phase was separated and water phase extracted with ethyl acetate (2 × 5 mL). Combined organic extracts were washed with brine, dried over Na_2SO_4, and concentrated in vacuo. The residue (acid **6,** 270 mg, 60%) was used further without purification.

ether/EtOAc = 3:1 then 2:1) gave vinyl carbamate **5** (537 mg, 45%) as a colorless oil.

^{1}H-NMR (500 MHz, CD_3OD): δ = 6.48 (d, J = 14.2 Hz, 1H), 5.11 (dt, J = 14.2, 7.2 Hz, 1H, 1H), 3.73 (s, 3H), 2.61-2.71 (m, 1H), 2.10-2.25 (m, 2H), 1.53-1.65 (m, 2H), 1.26 (d, J = 6.9 Hz, 3H) ppm; ^{13}C-NMR (125 MHz, CD_3OD): δ = 156.8, 156.7, 125.9, 110.6, 103.4, 93.5, 52.7, 37.6, 28.6, 26.2, 20.3 ppm.

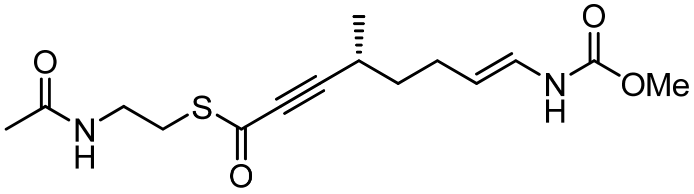

Diisopropylethylamine (49 µL, 0.3 mmol) and ethyl chloroformate (25 µL, 0.26 mmol) were added successively to a solution of acid **6** (45 mg, 0.2 mmol) in acetone (2 mL) at 0 °C. The reaction mixture was stirred for 1 h, potassium carbonate solution (5%, 0.7 mL) and N-acetylcysteamin (30 mg, 27 µL, 0.25 mmol) were added and stirring was continued for 1 h. The mixture was diluted with water (10 mL) and extracted with EtOAc (2 × 5 mL). The combined organic extracts were washed with brine, dried over Na_2SO_4, and concentrated in vacuo. The residue was purified by column chromatography (CH_2Cl_2/MeOH = 4:1) to give the thioester **7** (24 mg, 37%).

^{1}H-NMR (500 MHz, CD_3OD): δ = 6.43 (d, J = 14.2 Hz, 1H), 5.10 (dt, J = 14.2, 7.2 Hz, 1H, 1H), 3.71 (s, 3H), 3.37-3.42 (m, 3H), 3.11-3.15 (m, 2H), 2.10-2.26 (m, 2H), 1.97 (s, 3H), 1.60 (q, J = 7.3 Hz, 2H), 1.27 (d, J = 6.9 Hz, 3H) ppm; ^{13}C-NMR (125 MHz, CD_3OD): δ = 177.2, 173.5, 156.8, 126.1, 110.4, 100.4, 79.9, 52.7, 39.8, 37.4, 30.2, 28.6, 26.6, 22.5, 20.1 ppm.

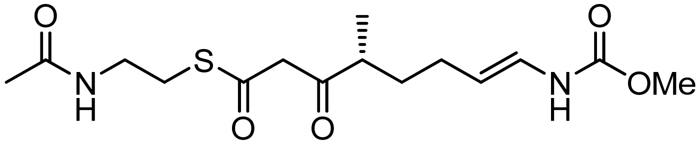

A solution of akylnyl ester 7 (10 mg, 0.03 mmol) and piperidine (3 μL) in ether (0.5 ml) was stirred overnight at rt. After evaporation of the solvent under reduced pressure the residue was dissolved in 1 mL of THF/H_2O = 3:1 and 20 mg of SiO_2 were added. After 3 hours, SiO_2 was filtered off, the filtrate was evaporated and the residue was subjected to column chromatography on silica (CH_2Cl_2/EtOAc = 4:1 followed by CH_2Cl_2/MeOH = 4:1). The product containing fractions were identified by HPLC-MS and evaporated to give β-ketothioester **8.** [15]

LC/MS Traces of SNAC Substrate 6 (JHS486)

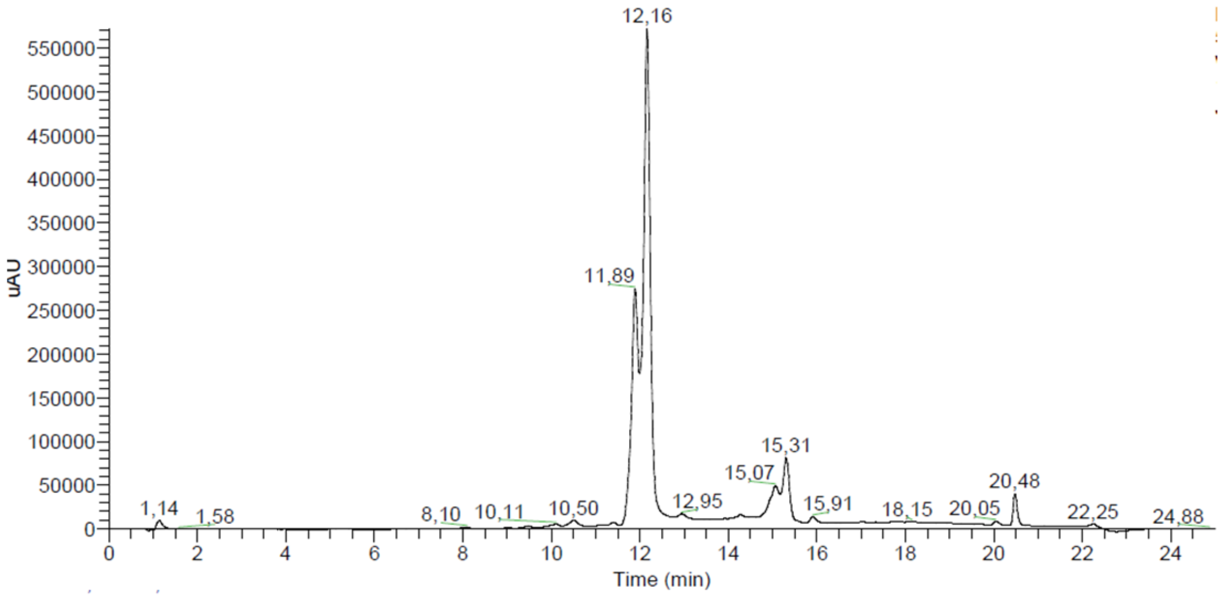

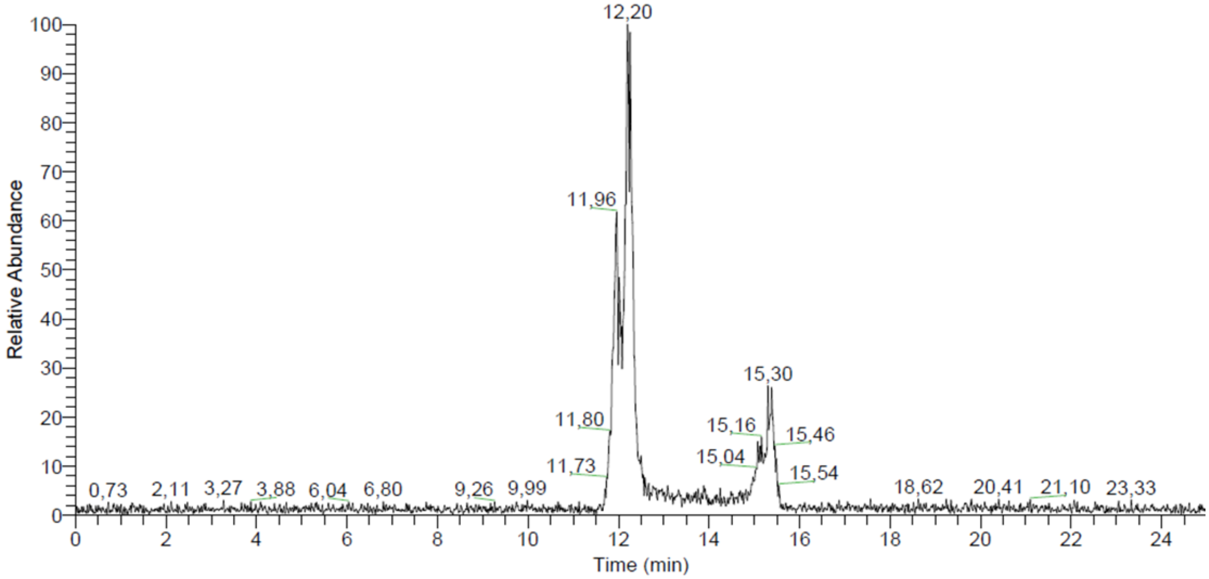

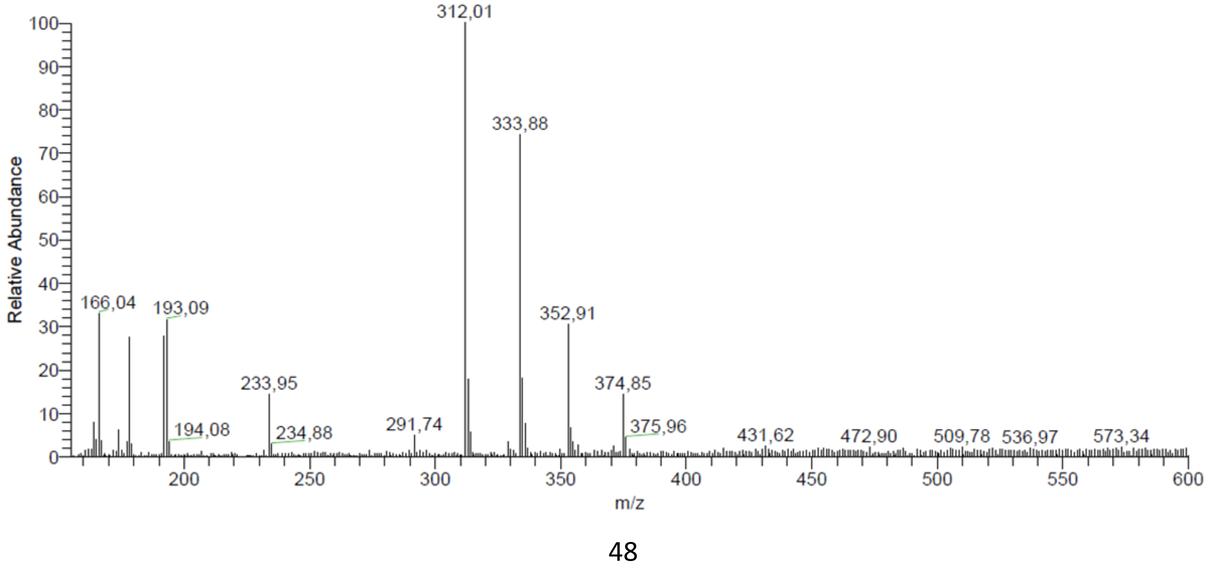

^{1}H NMR Spectrum of Substrate 6 (JHS486)

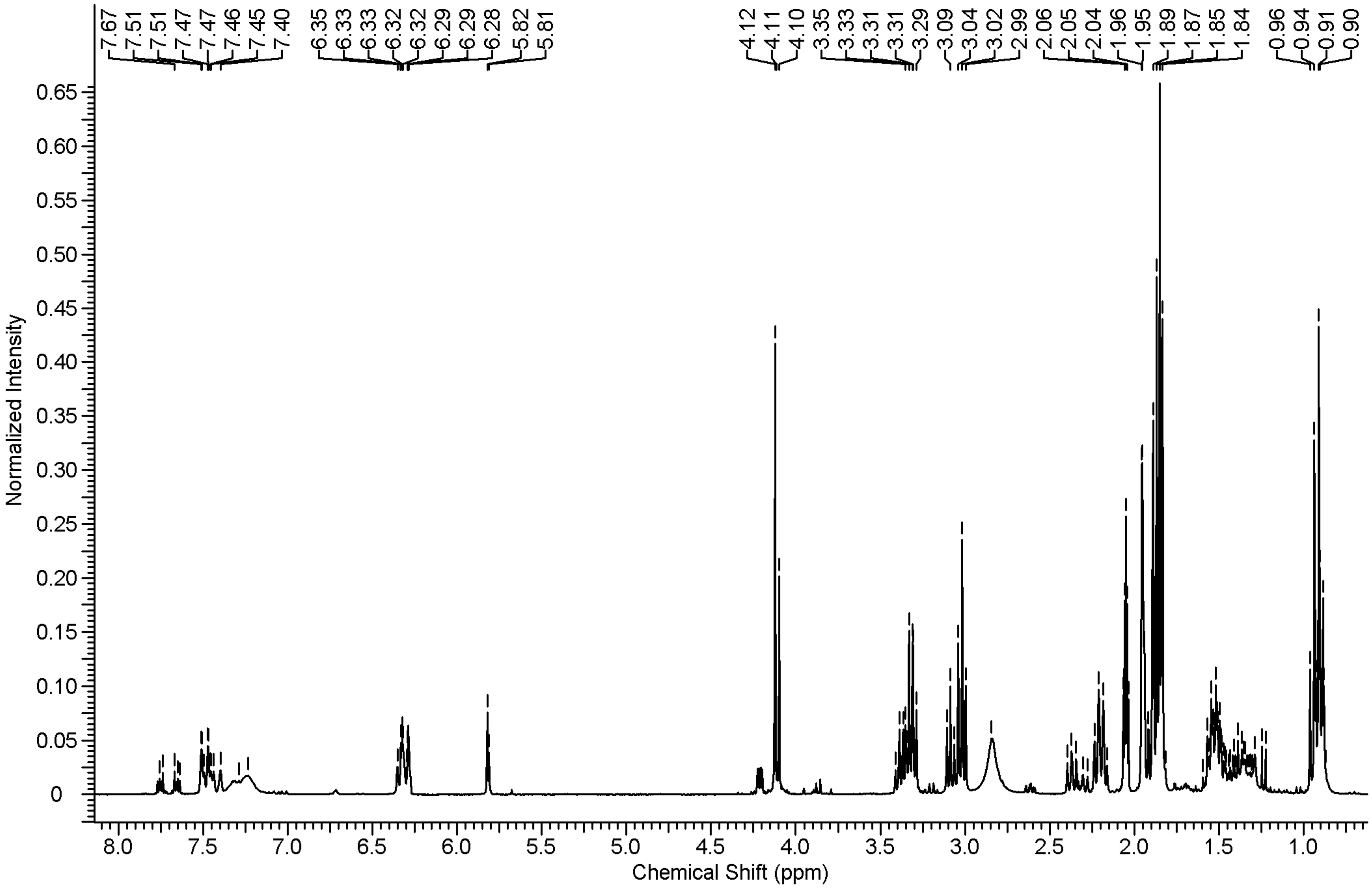

^{13}C NMR Spectrum of Substrate 6 (JHS486)

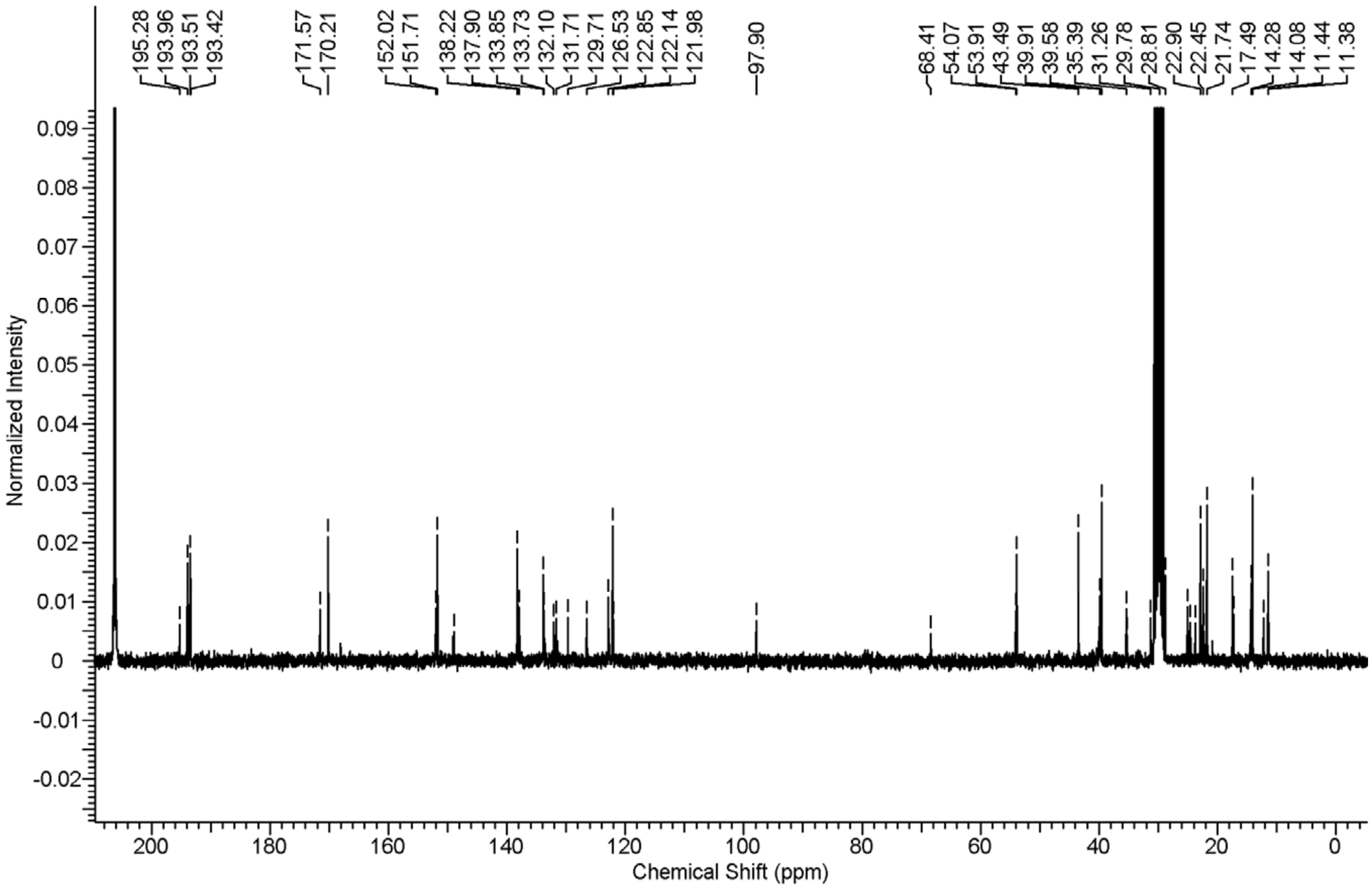

LC/MS Traces of SNAC Substrate 8 (JHS274)

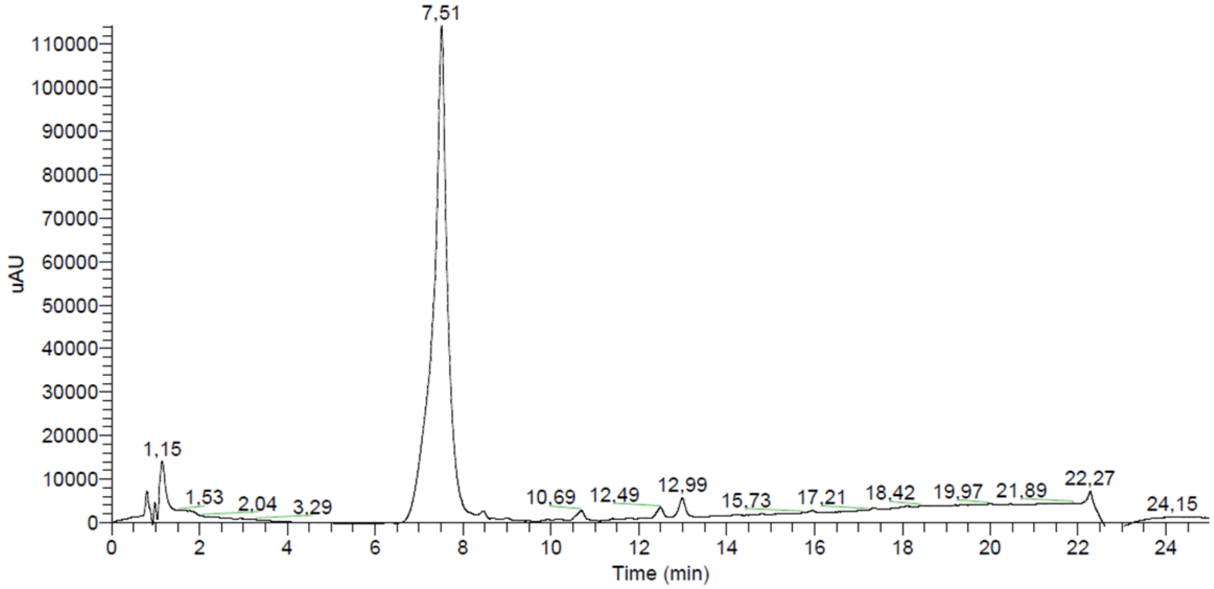

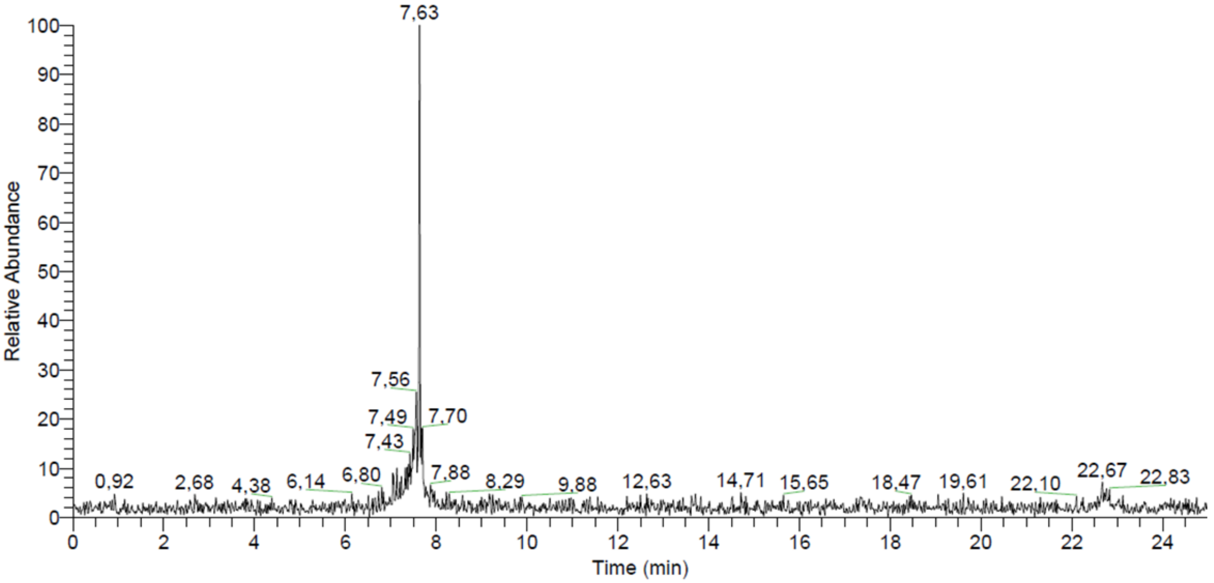

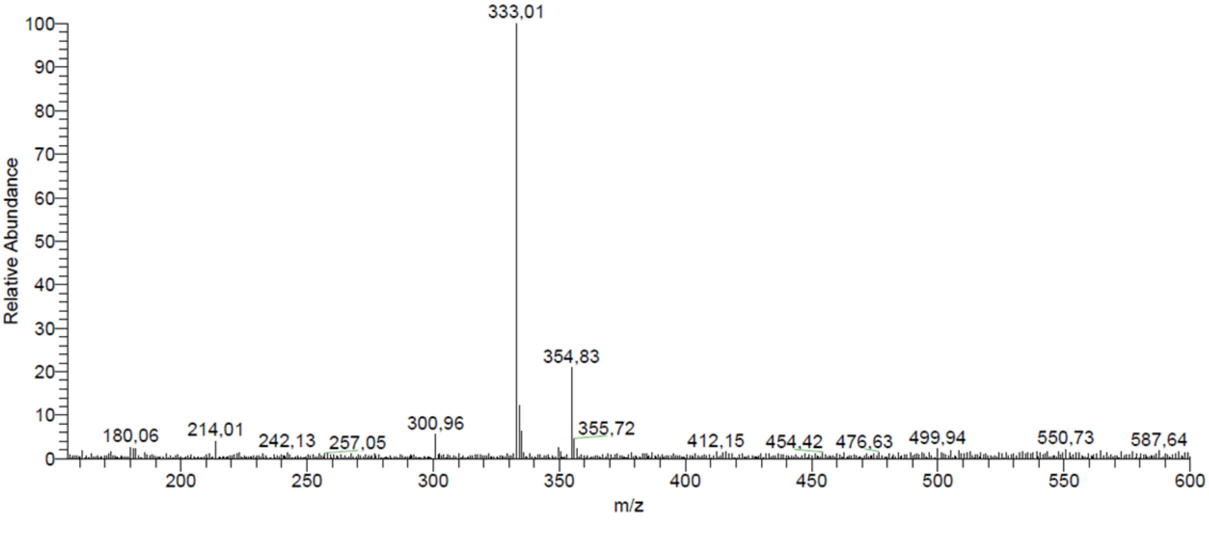

¹H NMR Spectrum of Substrate 8 (JHS274)

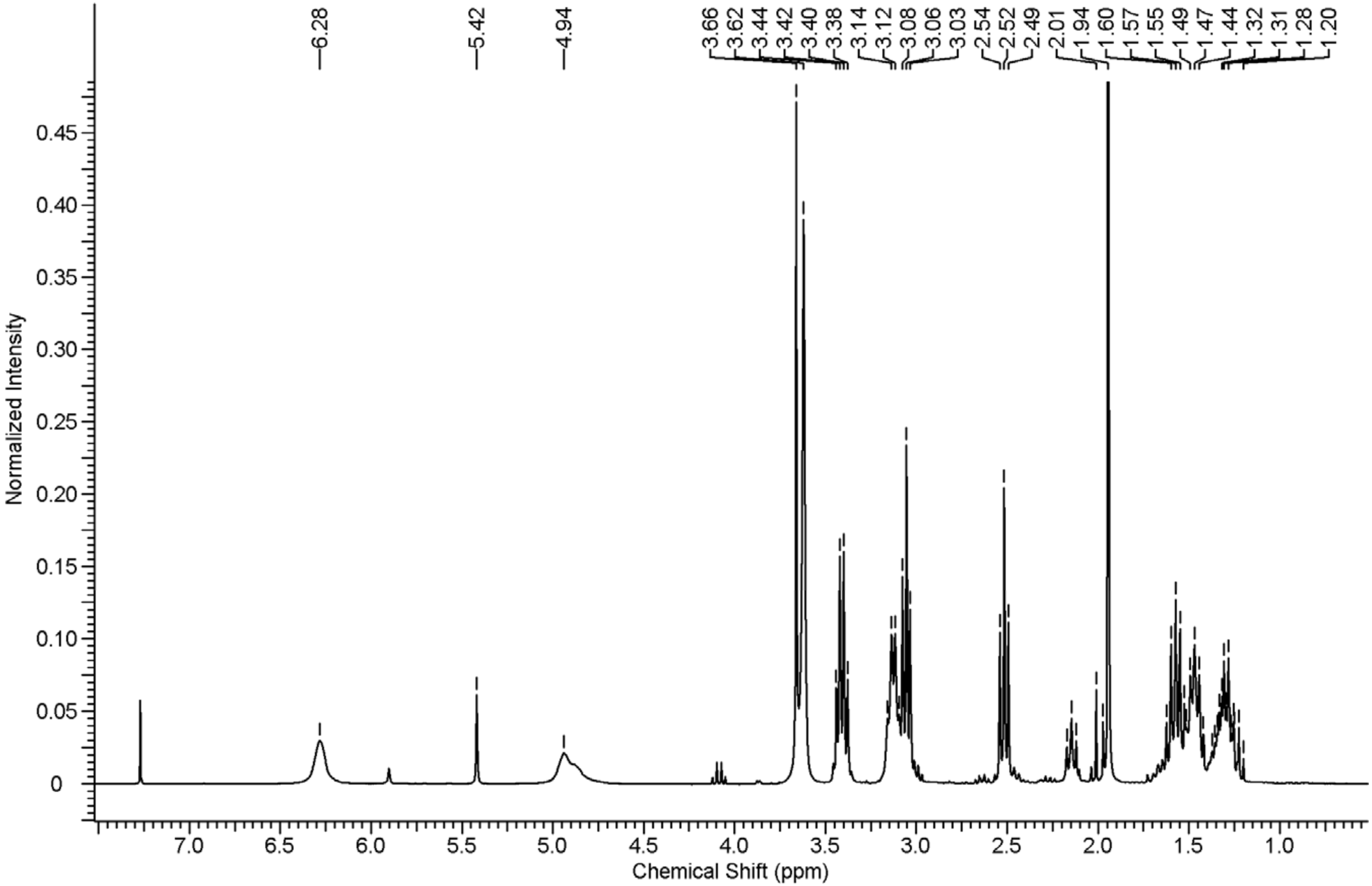

^{13}C NMR Spectrum of Substrate 8 (JHS274)

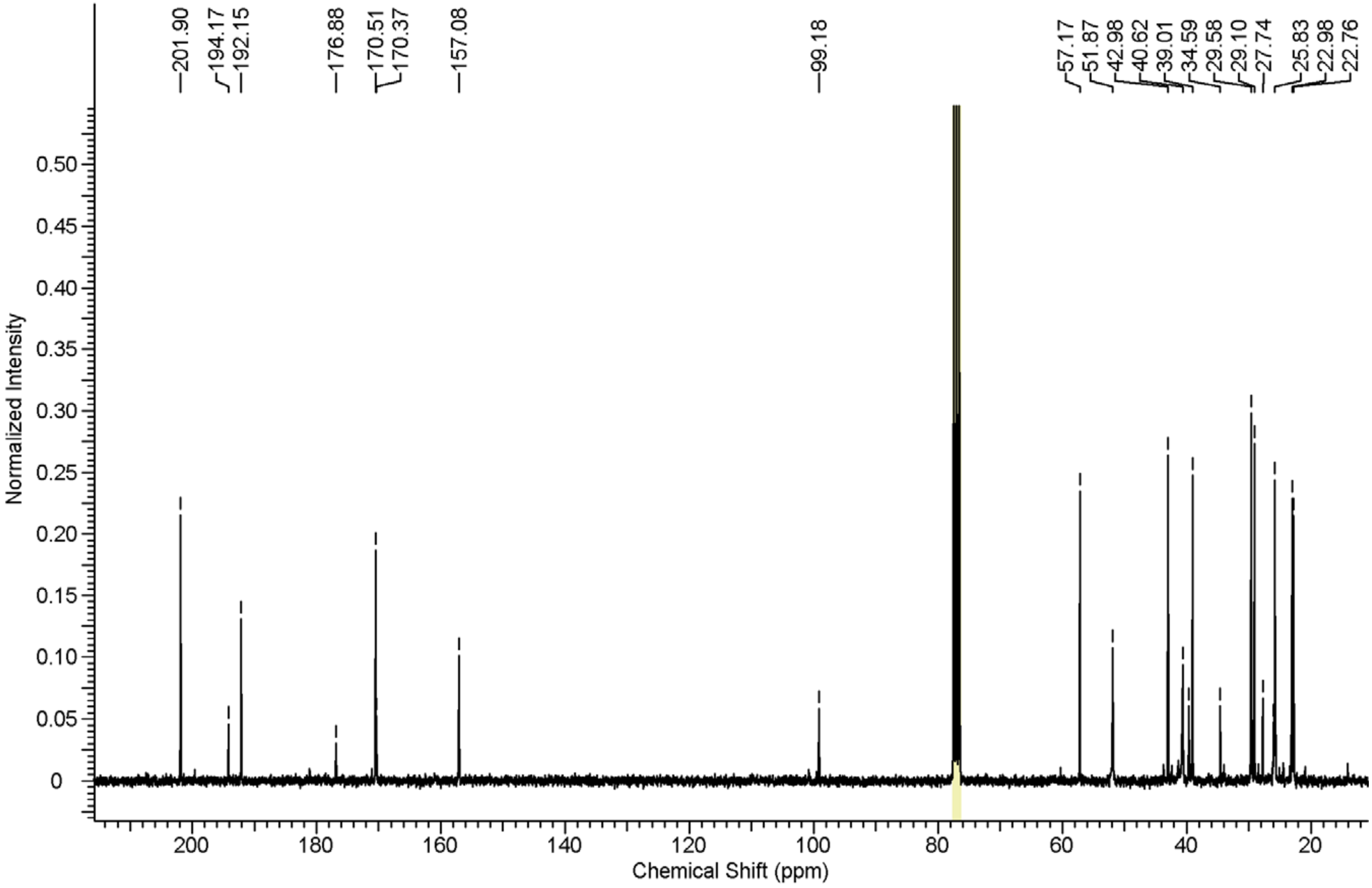

^{1}H NMR Spectrum of Compound 7 from Scheme 3

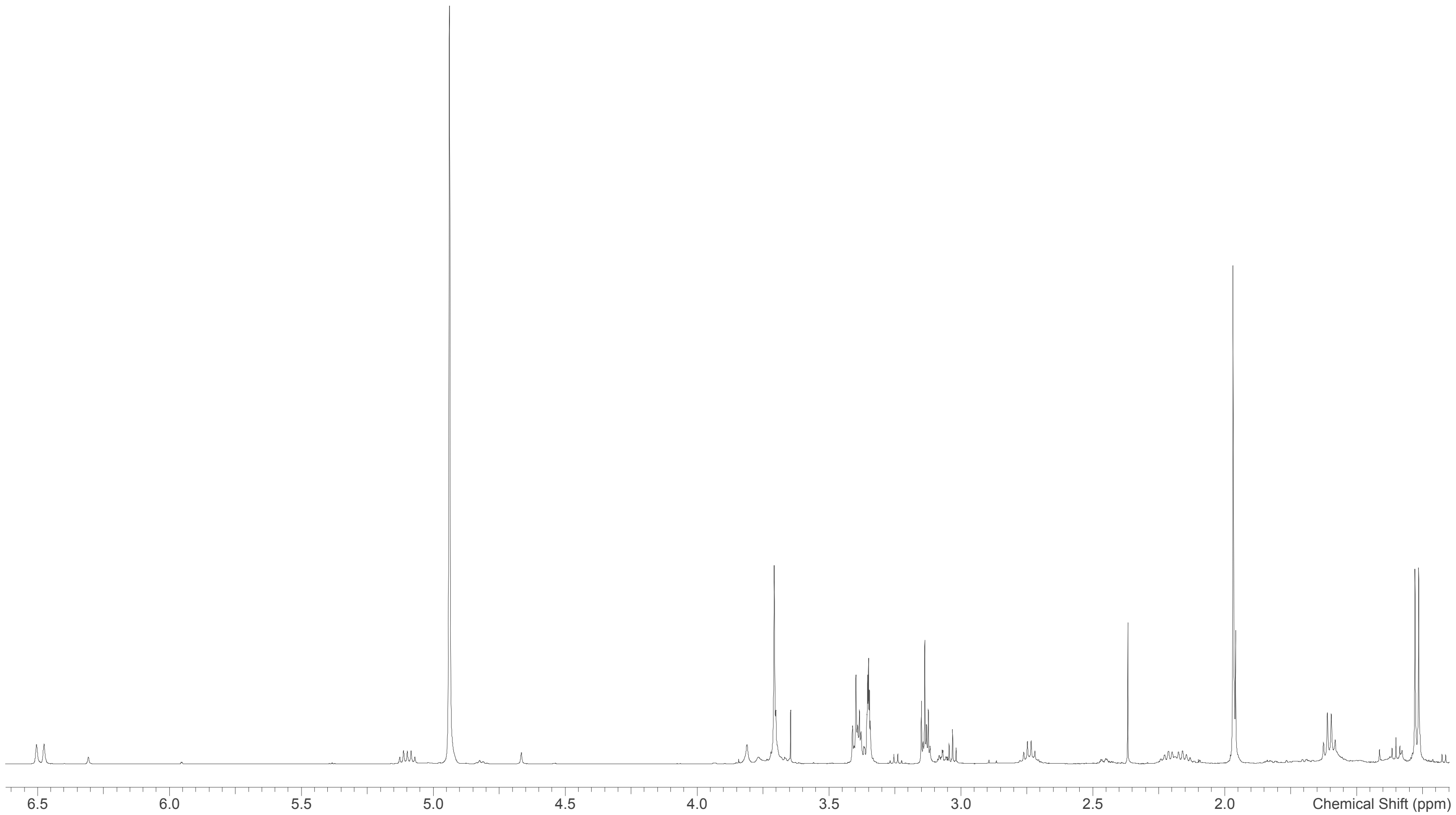

^{13}C NMR Spectrum of Compound 7 in Scheme 3

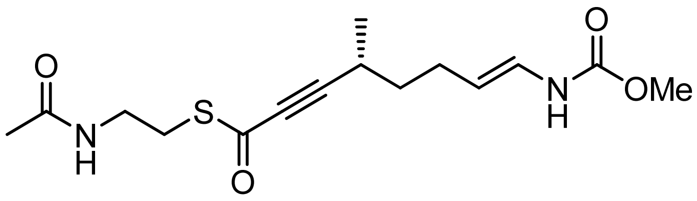

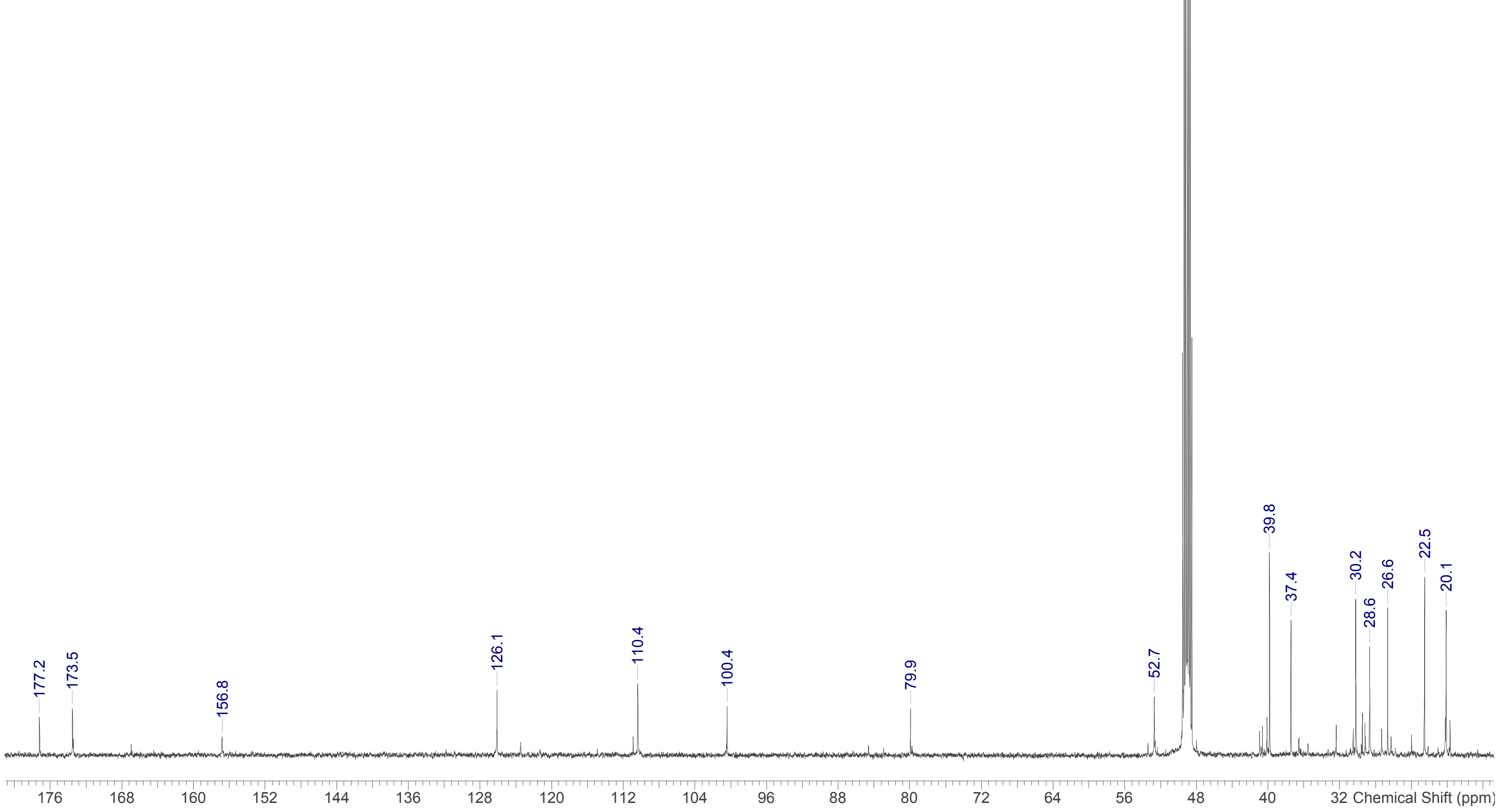

LC/MS Traces of SNAC Substrate 7 (Compound 8 in Scheme 3)

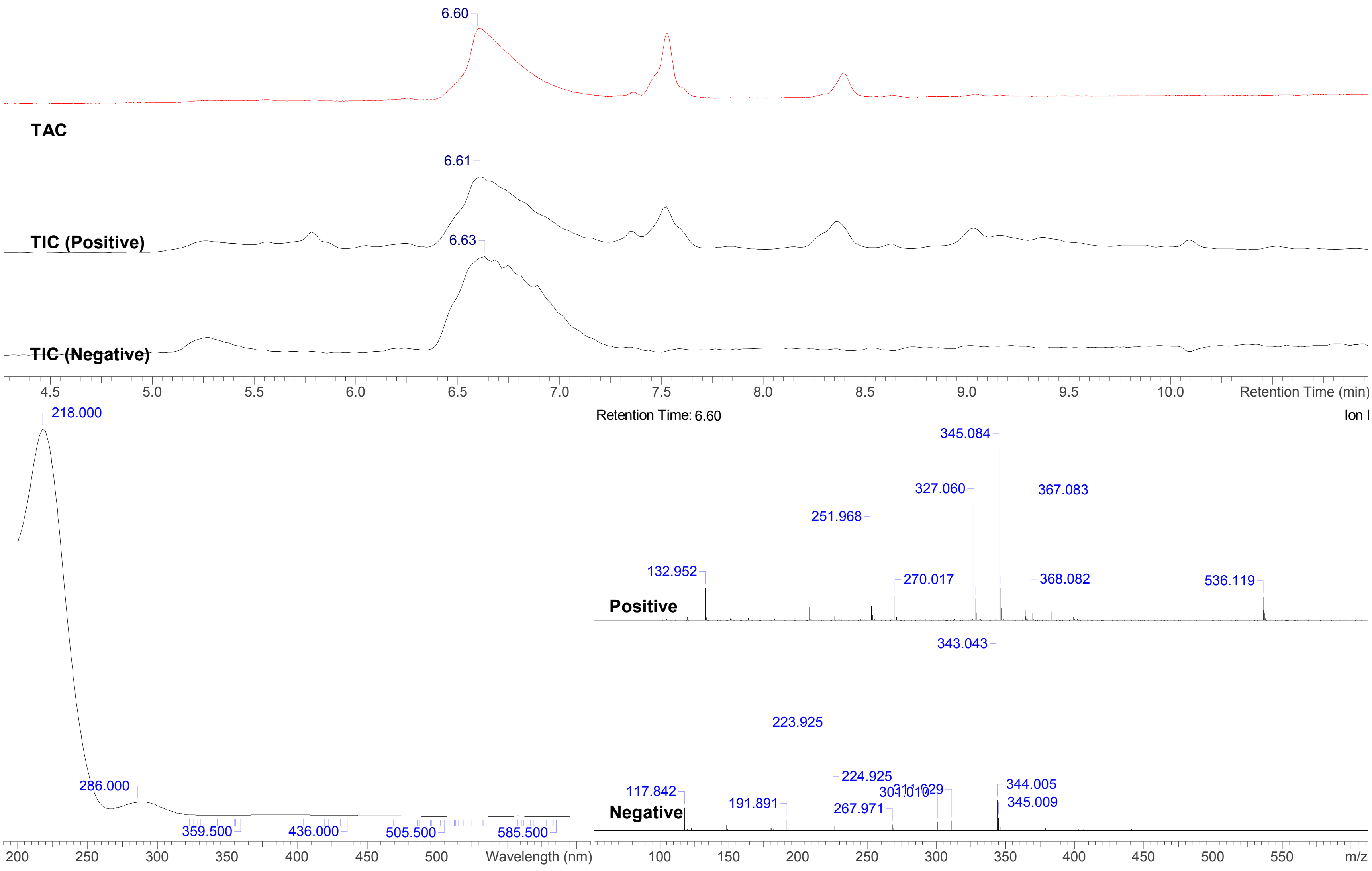

Reference

1 M. M. Bradford, *Anal. Biochem.*, 1976, **72**, 248–254.

2 A. J. McCoy, R. W. Grosse-Kunstleve, L. C. Storoni and R. J. Read, *Acta Crystallogr. D Biol. Crystallogr.*, 2005, **61**, 458–464.

3 L. C. Storoni, A. J. McCoy and R. J. Read, *Acta Crystallogr. D Biol. Crystallogr.*, 2004, **60**, 432–438.

4 P. Emsley and K. Cowtan, *Acta Crystallogr. D Biol. Crystallogr.*, 2004, **60**, 2126–2132.

5 G. N. Murshudov, P. Skubák, A. A. Lebedev, N. S. Pannu, R. A. Steiner, R. A. Nicholls, M. D. Winn, F. Long and A. A. Vagin, *Acta Crystallogr. D Biol. Crystallogr.*, 2011, **67**, 355–367.

6 P. D. Adams, K. Gopal, R. W. Grosse-Kunstleve, L.-W. Hung, T. R. Ioerger, A. J. McCoy, N. W. Moriarty, R. K. Pai, R. J. Read, T. D. Romo, J. C. Sacchettini, N. K. Sauter, L. C. Storoni and T. C. Terwilliger, *J. Synchrotron Radiat.*, 2004, **11**, 53–55.

7 H. Irschik, K. Gerth, G. Höfle, W. Kohl and H. Reichenbach, *J. Antibiot.*, 1983, **36**, 1651–1658.

8 1 N. Gaitatzis, B. Kunze and R. Müller, *Proc. Natl. Acad. Sci. U. S. A.*, 2001, **98**, 11136–11141.

9 1 B. M. Trost, J. P. N. Papillon and T. Nussbaumer, *J. Am. Chem. Soc.*, 2005, **127**, 17921–17937.

10 R. K. Boeckman, T. M. Kamenecka, S. G. Nelson, J. R. Pruitt and T. E. Barta, *Tetrahedron Lett.*, 1991, **32**, 2581–2584.

11 T. Yoshinari, K. Ohmori, M. G. Schrems, A. Pfaltz and K. Suzuki, *Angew. Chem. Int. Ed. Engl.*, 2010, **49**, 881—885.

12 I. H. Gilbert, M. Ginty, J. A. O'Neill, T. J. Simpson, J. Staunton and C. L. Willis, *Bioorg. Med. Chem. Lett.*, 1995, **5**, 1587–1590..

13 S. P. Raillard, W. Chen, E. Sullivan, W. Bajjalieh, A. Bhandari and T. A. Baer, *J. Comb. Chem.*, 2002, **4**, 470–474.

14 J. H. Sahner, H. Sucipto, S. C. Wenzel, M. Groh, R. W. Hartmann, and R. Müller, Epub ahead of print, DOI: 10.1002/cbic.201402666

15 (*a*) A. Rentsch and M. Kalesse, *Angew. Chem. Int. Ed. Engl.*, 2012, **51**, 11381—11384.; (*b*) C. Lu, B. Kirsch, C. Zimmer, de Jong, Johannes C, C. Henn, C. K. Maurer, M. Müsken, S. Häussler, A. Steinbach and R. W. Hartmann, *Chem. Biol.*, 2012, **19**, 381–390;
